# Synthesis and systematic evaluation of four stereoisomers of BMS compound A enabled by a diastereoselective cyclopropanation on a chiral ketal intermediate

**DOI:** 10.1039/d6ra02434c

**Published:** 2026-07-12

**Authors:** Feijun Wang, John N. Hanson, Snezana T. Dimova, Jayachandra Rayadurgam, Camryn J. Fulton, Amy E. Moritz, Ashley N. Nilson, William A. Hearne, Chun-Hsing Chen, Ryan H. Gumpper, David R. Sibley, Kevin J. Frankowski

**Affiliations:** a Chemical Biology and Medicinal Chemistry Division, UNC Eshelman School of Pharmacy, University of North Carolina at Chapel Hill Chapel Hill North Carolina USA kevinf@unc.edu; b Molecular Neuropharmacology Section, National Institute of Neurological Disorders and Stroke, National Institutes of Health Bethesda Maryland USA sibleyd@ninds.nih.gov; c Department of Pharmacology, UNC School of Medicine, University of North Carolina at Chapel Hill Chapel Hill North Carolina USA; d Department of Chemistry X-ray Crystallography Core, University of North Carolina at Chapel Hill Chapel Hill North Carolina USA

## Abstract

The D_1_ dopamine receptor (D_1_R) ligand BMS Compound A is a positive allosteric modulator (PAM) of D_1_R signaling. BMS Compound A binds specifically to one of three known allosteric sites on the D_1_R and is a useful chemical tool for the investigation of D_1_R pharmacology. Despite the utility of BMS Compound A, even small quantities of the molecule are not commercially available. Here we report two variations on a convergent synthetic approach to construct this tool molecule. The first route is a four-step sequence to afford the four stereoisomers of BMS Compound A. The second route leverages a diastereoselective cyclopropanation on a chiral ketal substrate. Carrying this single enantiomer intermediate through our previous convergent reaction sequence affords two separable diastereomers of BMS Compound A—each a single enantiomer. We characterized the four stereoisomers of BMS Compound A, assigned their absolute configuration, specific rotation, ^13^C nuclear magnetic resonance (NMR) spectra. We further evaluated their ability to activate D_2_R signaling or potentiate dopamine-mediated D_1_R signaling. We found the greatest difference in D_1_R PAM activity for *syn vs. anti* configurations and less drastic differences between enantiomers. We also determined that the four stereoisomers exhibited agonist activity at the D_2_R.

## Abstract 2

The D_1_ dopamine receptor (D_1_R) ligand BMS Compound A is a positive allosteric modulator (PAM) of D_1_R signaling.

## Graphical abstract

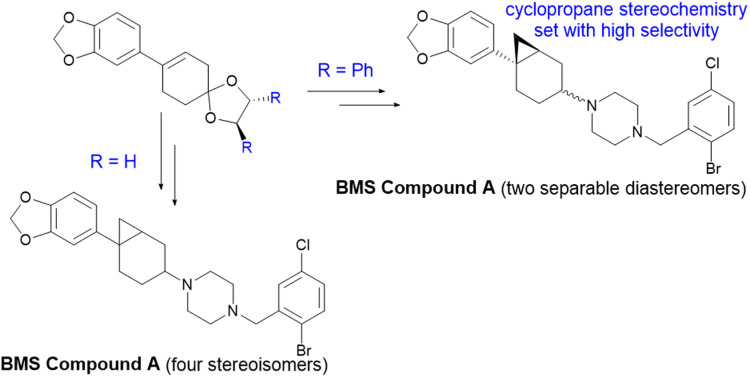

## Introduction

Allosteric modulators of G protein-coupled receptors (GPCRs) can produce three functional effects, which are not mutually exclusive: (1) affect the affinity of ligands, either positively or negatively, for binding to the receptor's orthosteric site; (2) affect the efficacy of agonists, either positively or negatively, for producing a functional response; (3) elicit activation of the receptor in the absence of agonists.^1–3^ Positive allosteric modulators (PAMs) enhance the effects of orthosteric ligands, while negative allosteric modulators (NAMs) reduce the effects of orthosteric ligands. Allosteric modulators that elicit an active receptor response are called allosteric agonists. Allosteric modulators that function as both PAMs and agonists are sometimes referred to as ago-PAMs while allosteric ligands that bind to the receptor without producing an effect on orthosteric ligand binding or receptor signaling are referred to as either silent allosteric modulators or neutral allosteric ligands.^1–3^ In the context of the D_1_ dopamine receptor (D_1_R), PAMs have emerged as a potential therapeutic approach for treating the cognitive dysfunction of neuropsychiatric disorders, such as schizophrenia, Parkinson's disease and age-related cognitive decline.^4,5^ Multiple D_1_R PAMs have been reported with three distinct allosteric binding sites on the receptor identified thus far.^6,7^ Simultaneously targeting of these three allosteric sites of the D_1_R has demonstrated a synergistic potentiation of dopamine (DA)-mediated D_1_R signaling of up to 1000-fold.^8,9^ Such multisite targeting may offer unique opportunities for fine-tuning receptor function over a wide range of activities and enable precise control of receptor signaling providing a unique therapeutic advantage over single-site modulation.

**Fig. 1:**
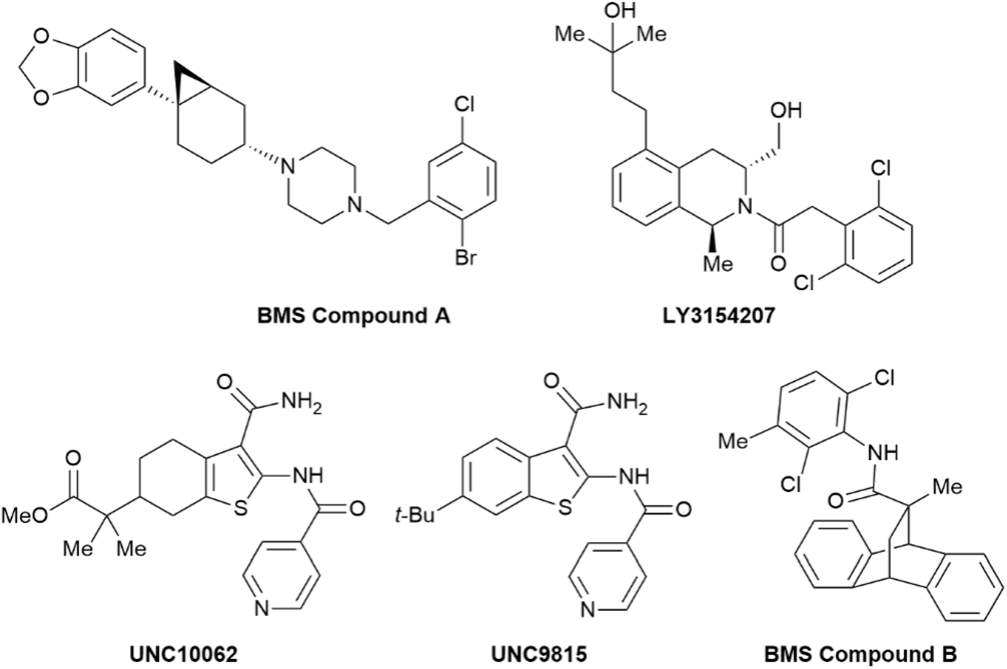
Structures of D1R PAM molecules discussed in this article.

We recently leveraged three complementary D_1_R PAMs: LY3154207, BMS Compound A, and UNC10062/UNC9815 and cryo-EM technologies to characterize the location and receptor conformational changes resulting from binding to three distinct allosteric sites on the D_1_R (Fig. 1). This structural biology effort identified the precise location of all three allosteric sites and revealed an unprecedented mechanism of cooperative allosteric modulation in the D_1_R.^10^ Of note, BMS Compound A is the only compound currently known to bind to its unique allosteric site on the D_1_R thus rendering it an important tool compound to investigate D_1_R allosteric pharmacology.

BMS Compound A is a piperazine-based D1R PAM that was initially discovered from a high-throughput screening effort that aimed to identify potential therapeutic leads for treating schizophrenia.^11^ The anthracene-based BMS Compound B was also identified from the same screen and identified as a D_1_R PAM. Following preliminary characterization of the screening hits, interest favored the development of Compound B due to the D_2_R agonism (EC_50_ = 330 nM) of Compound A, which could exacerbate the positive symptoms of schizophrenia. While Bristol Myers Squibb subsequently discontinued the development of Compound B, Eli Lilly had identified a D_1_R PAM that was developed into the drug lead LY3154207 and further determined that it binds to the same allosteric site as BMS Compound B.^8^ The Lilly group also provided evidence that BMS Compound A binds to a distinct allosteric site when compared to other D_1_R PAMs (*e.g.*, BMS Compound B, LY3154207, and thiophene PAMs such as UNC10062/UNC9815).^8^ Comprehensive potentiation studies showed that BMS Compound A engendered a 24-fold potentiation of D_1_R signaling and that in combination with representatives of the other two PAM classes, D_1_R potentiation of nearly 1000-fold could be attained. The initial report provided a single stereo-defined structure for BMS Compound A, however it did not disclose absolute stereochemistry or other stereoisomers.^10^ The Eli Lilly study separated all four stereoisomers and identified a single *anti* enantiomer (the second eluting stereoisomer) as the most potent, naming it “BMS-A1”, and not determining absolute configuration.^8^ We will use the term BMS Compound A from the initial publication to refer to all four stereoisomers and differentiate stereoisomers by their relative (*syn* or *anti*) and absolute (*R* or *S*) configurations or by their order of elution from a chiral high-performance liquid chromatography (HPLC) column.

The exclusive allosteric binding site for BMS Compound A, applicability to rodent models, and contributions to synergistic D_1_R signaling make this molecule a valuable chemical tool for studying D_1_R signaling. Investigations using BMS Compound A have been limited as even small quantities of the molecule are not commercially available. Here we report a convergent synthetic route to this tool molecule and a diastereoselective variation that provides access, following flash chromatography, to any desired stereoisomer without the need for chiral HPLC purification. We leveraged our access to BMS Compound A to systematically characterize the physical and pharmacological properties of the four stereoisomers.

## Results and discussion

**Scheme 1:**
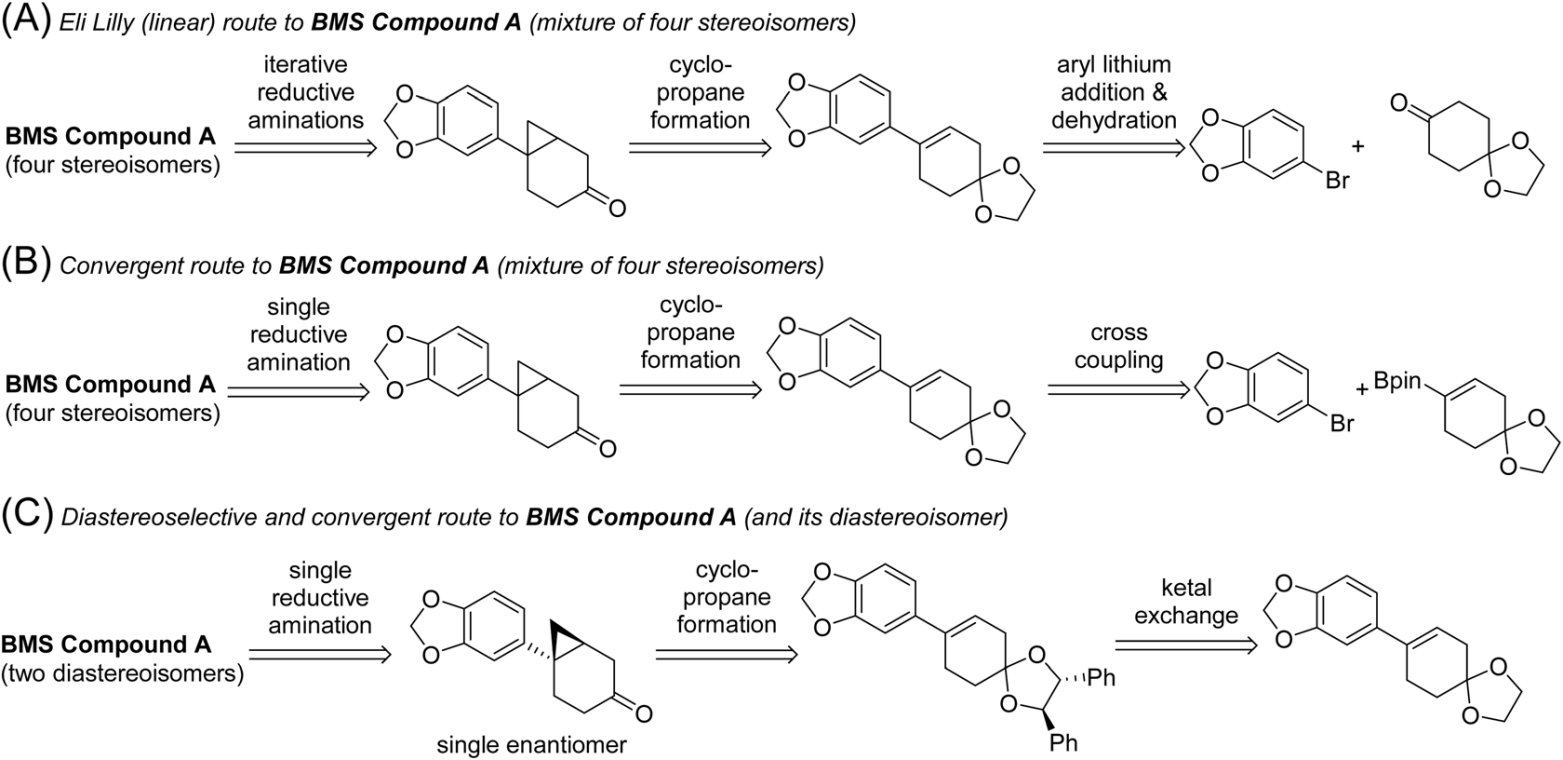
Retrosynthetic comparison of the published linear route to BMS Compound A and our convergent routes.

While the initial discovery of BMS Compound A from a high-throughput screen did not report its synthesis or assign absolute stereochemistry,^10^ researchers at Eli Lilly reported a synthetic route to BMS Compound A (Scheme 1A).^8^ This seven-step, linear route affords a mixture of four stereoisomers (all possible stereoisomers comprising a *syn* configuration of the cyclopropane ring) that must be separated using preparative chiral HPLC to obtain the most potent stereoisomer. The lengthy synthesis and requirement for specialized chromatography resources limit the laboratories capable of reproducing this synthesis and, thus, access to BMS Compound A remains a challenge for the research community. Our research program developing new D_1_R PAMs required significant quantities of previously reported D_1_R PAMs, and BMS Compound A is the only molecule known to bind its specific allosteric site. While reproducing the reported synthesis of BMS Compound A,^8^ we found the optimization of several synthetic steps to be more challenging than anticipated, leading to lower-than-expected yields of the target molecule. To improve the overall yield of BMS Compound A from our synthetic efforts, we pursued a convergent approach that reduced the synthesis to a four-step linear sequence and optimized the aryl olefin construction (Scheme 1B). However, our first-generation approach still afforded the same mixture of four stereoisomers and required preparative chiral HPLC separation. In a second-generation convergent approach, we swapped the ethylene ketal for a chiral ketal group to afford a single enantiomer in the cyclopropanation step, which set the relevant stereocenters with high selectivity (Scheme 1C).^12^ Carrying this intermediate forward to the final reductive amination afforded only two diastereomeric isomers of BMS Compound A, which were readily separated using flash chromatography. The chiral ketal route allows selective access to any of the four stereoisomers of BMS Compound A without the use of chiral preparative HPLC purification. In this report, we detail the optimized synthetic sequence for both convergent routes and characterize all four stereoisomers using X-ray crystallography to determine absolute configuration, polarimetry properties, ^13^C nuclear magnetic resonance (NMR) spectroscopy, their ability to potentiate dopamine-mediated D_1_R signaling using both β-arrestin recruitment and cAMP accumulation assays, and also their activation of D_2_R signaling.

**Scheme 2:**
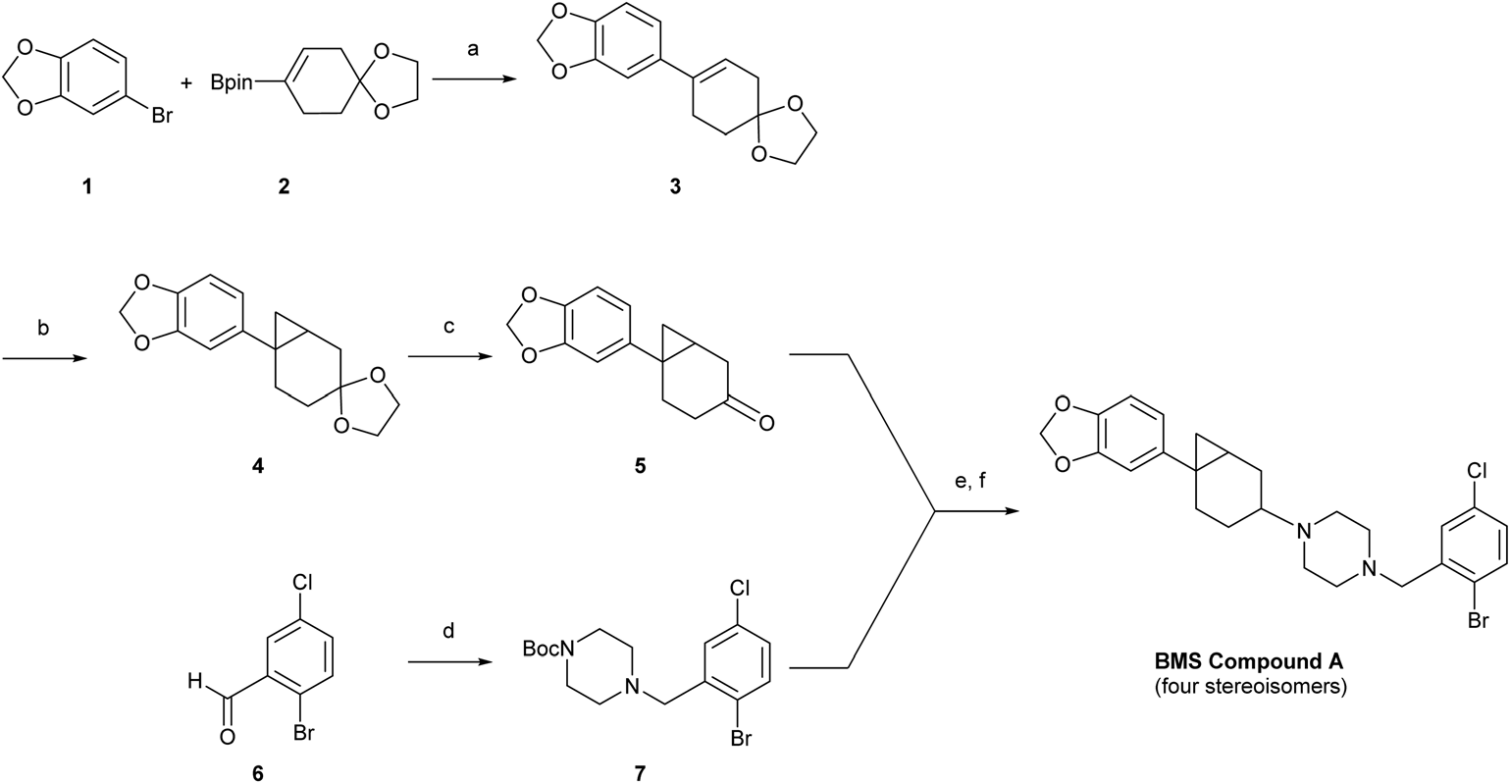
Convergent route to all four stereoisomers of BMS Compound A. Reagents and conditions: (a) Pd(PPh_3_)_4_ (5 mol%), Na_2_CO_3_ (2.0 equiv.), dioxane/water, 80 °C, 16 h, 80% yield; (b) CH_2_I_2_ (5.0 equiv.), ZnEt_2_ (3.0 equiv.), TFA (0.5 equiv.), DCM, 0 °C, 5 h; (c) HCl_aq_ (2.0 N), acetone, 40 °C, 13 h, 75% yield over two steps; (d) *N*-Boc-piperazine (1.5 equiv.), NaBH(OAc)_3_ (3.0 equiv.), AcOH (3 drops), DCM, 40 °C, 16 h, 83% yield; (e) Et_3_SiH (2.0 equiv.), TFA, DCM, rt, 5 h; (f) 5 (1.5 equiv.), NaBH(OAc)_3_ (3.5 equiv.), CHCl_3_, reflux, 16 h, 69% yield over two steps.

In our convergent approach to BMS Compound A, we coupled the benzaldehyde 6 and *N*-Boc-piperazine *via* reductive amination to afford the piperazine component 7 in a separate synthetic sequence (Scheme 2). Synthesis of the bicyclic component 5 began with an alternative formation of the aryl-substituted cyclohexene 3 (Scheme 2). We found the reported two-step aryl lithium formation/addition to cyclohexanone and dehydration sequence to often leave unreacted starting substrate and afford partial ketal deprotection. We replaced this sequence with a single-step Suzuki–Miyaura coupling where palladium-catalyzed cross-coupling of aryl bromide 1 and boronic ester 2 afforded the substituted cyclohexene 3. The olefin was homologated to cyclopropane under modified Simmons–Smith conditions and the crude ketal product subsequently hydrolyzed to give the bicyclic ketone 5. When we initially repeated the published synthetic route, we also found the reported final reductive amination challenging to optimize and observed significant reduction of the benzaldehyde over desired reductive amination. As noted above, we opted instead to separately synthesize the piperazine component 7, which avoided the challenging final reductive amination step and reduced the length of the longest linear sequence. The *N*-Boc piperzine 7 was deprotected with trifluoroacetic acid and the crude piperazine then coupled with ketone component 5 under reductive amination conditions to afford a mixture of the four previously reported stereoisomers of BMS Compound A (in accordance with the previous report by Eli Lilly, *syn* and *anti* refer to the stereochemical relationship between the cyclopropane ring and piperazine group).^8^ While the product ratio was identical to the reported synthesis, the longest linear sequence was reduced from seven to five steps with over 25% improvement in overall yield (33% to 42% overall yields).

While our convergent approach provided a more efficient synthesis to BMS Compound A (as a mixture of four stereoisomers) and avoided protocols that required exacting reaction optimization, isolation of individual stereoisomers still required preparative, chiral HPLC purification. The reliance on chiral HPLC was a synthetic bottleneck and limited our capacity to produce the desired stereoisomer of BMS Compound A on scale. In addition, the four stereoisomers of BMS Compound A were produced in approximately equal proportions and, thus, generated three undesired side-product molecules for every molecule of the desired stereoisomer —another major obstacle to increased production of the desired stereoisomer. We had observed that the *anti* and *syn* isomers of BMS Compound A were separable by flash chromatography and envisioned that if we could bring a single enantiomer of ketone 5 into the reductive amination coupling, the resulting *anti*/*syn* diastereomers could be readily separated to afford single enantiomers of BMS Compound A. We further hypothesized that a stereoselective cyclopropanation would allow us to adapt our convergent route with minimal new reaction development. We chose to investigate chiral ketal substrates, which we hoped would direct a diastereoselective cyclopropanation^12^ and, in the event of only partial diastereoselectivity, would allow for separation of any of the undesired diasterereomer prior to the final reductive amination.

**Scheme 3:**
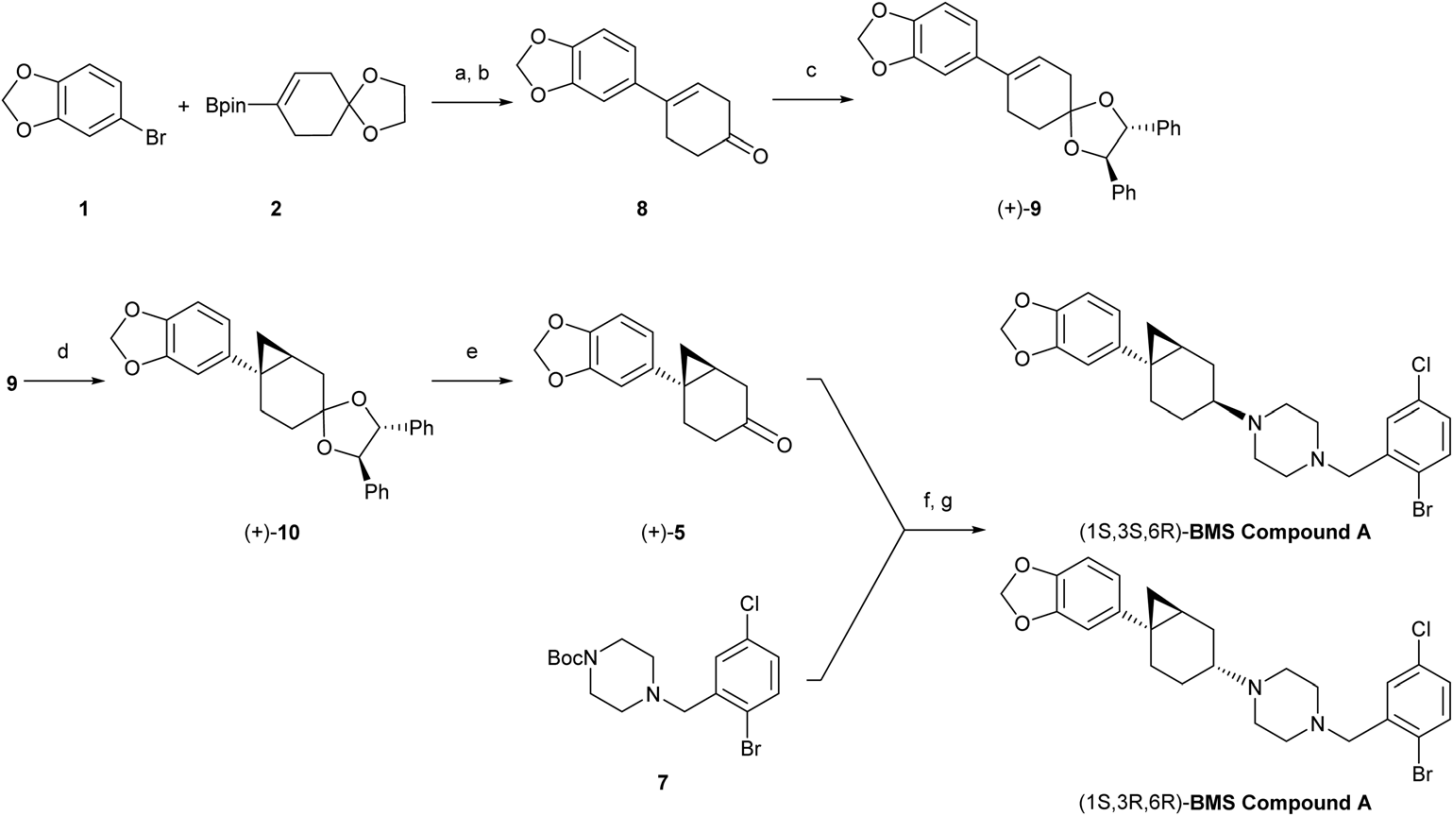
Diastereoselective and convergent route to single stereoisomers of BMS Compound A *via* flash chromatography separable diastereoisomers. Reagents and conditions: (a) Pd(PPh_3_)_4_ (5 mol%), Na_2_CO_3_ (2.0 equiv.), dioxane/water, 100 °C, 24 h; (b) HCl_aq_ (2.0 N), acetone, 40 °C, 13 h, 79% yield over two steps; (c) (1*R*,2*R*)-hydrobenzoin (1.1 equiv., 99% ee), pyridinium *p*-toluenesulfonate (0.2 equiv.), toluene, reflux, 12 h, 78% yield, 99% ee; (d) CH_2_I_2_ (5.0 equiv.), ZnEt_2_ (3.0 equiv.), TFA (0.5 equiv.), DCM, −15 °C, 10 h, 71% yield, 99% ee; (e) HCl_aq_ (2.0 N), acetone, 40 °C, 13 h, 95% yield; (f) Et_3_SiH (2.0 equiv.), TFA, DCM, rt, 5 h; (g) 5 (1.5 equiv.), NaBH(OAc)_3_ (3.5 equiv.), CHCl_3_, reflux, 16 h, 66% yield over two steps.

We began with the same cross-coupling of bromide 1 and boronic ester 2 from the convergent approach above and directly funneled the crude reaction product into a ketal deprotection step to afford the ketone 8 in 79% yield over both steps (Scheme 3). Reprotection of the ketone with the chiral diol (1*R*,2*R*)-hydrobenzoin (enantiopurity = 99% ee) afforded the enantiopure ketal 9. While this ketal could be directly prepared *via* cross-coupling with a chiral ketal boronate, the need to synthesize such a boronate starting material led us to favor the deprotection/reprotection route. We were pleased to find that the previous cyclopropanation protocol on chiral ketal substrate 9 afforded the bicyclic ketal 10 as a single isolated diastereomer and high enantiopurity (e.r. = 99.5 : 0.5). Removal of the chiral ketal then provided ketone (+)-5 in high enantiopurity. The commercial availability of either enantiomer of the hydrobenzoin diol allows access to either enantiomer of ketone 5 desired. As expected, the reductive amination coupling of enantiopure ketone (+)-5 and piperazine 7 afforded a separable diastereomeric mixture of two BMS Compound A stereoisomers, which were isolated individually following flash chromatography in roughly equal proportion (31% and 35% yields, respectively). This synthesis demonstrates a practical route to any of the four previously reported stereoisomers of BMS Compound A without the need for chiral, preparative HPLC purification.

**Fig. 2:**
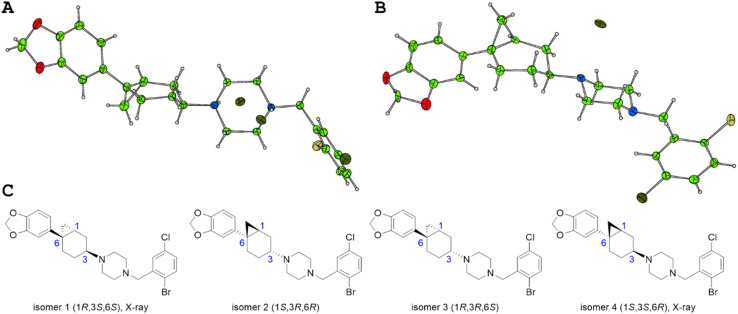
Molecular structures of (+)-(1*R*,3*S*,6*S*)-BMS Compound A (isomer 1) (A) and (−)-(1*S*,3*S*,6*R*)-BMS Compound A (isomer 4) (B) with labels on the asymmetric unit. Structures of all four assigned stereoisomers of BMS Compound A (C).

We separated the mixture of four stereoisomers from our convergent route using chiral preparative HPLC purification (Chiralpak ADH 30 × 250, 98 : 2 hexanes:ethanol with 0.1% diethylamine additive). The separation used the previously reported column and slightly modified eluents and conditions, which afforded the individual enantiomers of BMS Compound A in the same order of elution as previously reported.^8^ Eli Lilly determined that the second eluting stereoisomer was the most potent, naming it “BMS-A1”. The ^1^H NMR spectra of stereoisomers matched those previously reported and we acquired the ^13^C NMR spectra to complement existing characterization data.^9^ To establish the absolute configuration of the four isomers, we took advantage of the chlorine and bromine atoms present in BMS Compound A to facilitate anomalous diffraction.^13^ Isomer 1 (representative *syn* isomer) and isomer 4 (representative *anti* isomer) were converted to their hydrochloride salt forms and recrystallized (isomer 1 from methanol/ethanol/water and isomer 4 from ethanol/ethyl acetate) to afford single crystals suitable for X-ray diffraction. The X-ray diffraction results revealed the absolute configuration of isomer 1 (CCDC deposition number 2540023) and isomer 4 (CCDC deposition number 2540093) and, by extension, the absolute configuration of their antipodes (isomers 2 and 3, respectively). The ORTEP diagram representations for isomers 1 and 4 are provided in Fig. 2A and B respectively, and the absolute configuration of all four stereoisomers is summarized in Fig. 2C. We then characterized the individual enantiomers by polarimetry (specific rotation) to allow subsequent enantiomer identification without chiral HPLC equipped the specific (ADH) column previously used. As shown in Table 1, the (+)-enantiomer was the earlier eluting enantiomer in both cases. The direction of rotation for each isomer (*i.e.*, (+) or (−) rotation) in combination with NMR spectroscopy can now be used to assign the identity of any individual stereoisomer.

**Table 1:** Specific rotation correlation with each stereoisomer of BMS Compound A

Chiral HPLC stereoisomer elution order	Relative cyclohexane stereochemistry (cyclopropyl *vs.* piperzine groups)	Absolute stereochemical assignment	Specific rotation[*α*]^20^_D_ (*c* = 0.5 g per 100 mL, CHCl_3_)
1	*anti*	1*R*,3*S*,6*S*	+27.0
2 (BMS-A1)	*anti*	1*S*,3*R*,6*R*	−27.2
3	*syn*	1*R*,3*R*,6*S*	+25.0
4	*syn*	1*S*,3*S*,6*R*	−25.8

**Fig. 3:**
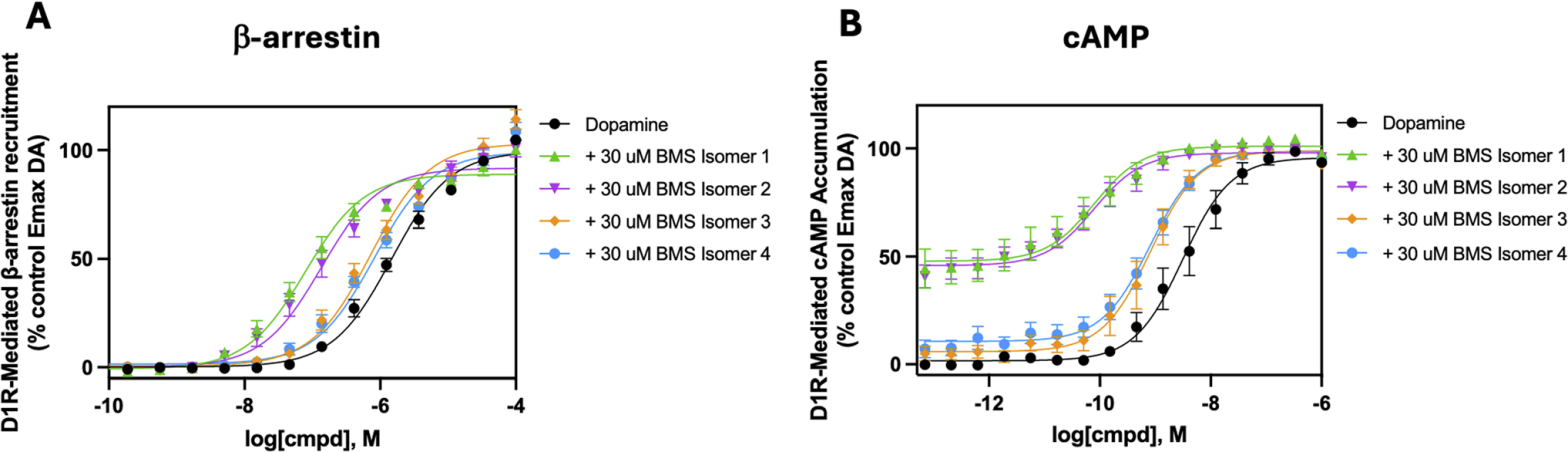
Effect of BMS Compound A isomers on dopamine potency at the D_1_R in both the β-arrestin recruitment (A) and cAMP accumulation assays (B). The DiscoveRx Pathhunter β-arrestin recruitment and LANCE cAMP assays were conducted as described in the Experimental Section. Dopamine concentration-response curves (CRCs) were performed in the absence or presence of 30 µM of each isomer, which was determined to be a maximally effective concentration in preliminary concentration-response experiments (Fig. S1). Note that the lowest concentration (open circle symbol) in each CRC was performed in the absence of DA and used to define the baseline response (0%) in each experiment. The DA *E*_max_ in the absence of PAMs was used to define the maximal response (100%) in each experiment. The data are representative of >3 independent experiments. See Tables 2 and 3 for average curve parameters and statistical analyses.

We next characterized all four BMS Compound A stereoisomers for their D_1_R PAM activity using β-arrestin recruitment and cAMP accumulation assays (Fig. 3). Our data reinforce previously reported results and provide systematic pharmacological characterization of all four stereoisomers.^8^ All BMS Compound A stereoisomers exhibit D_1_R PAM activity in both the β-arrestin recruitment and cAMP accumulation assays with the *anti* stereoisomers (isomers 1 and 2) providing more effective potentiation than the *syn* configuration (isomers 3 and 4). In the β-arrestin recruitment assay, isomers 1 and 2 produced a statistically significant ∼15-fold increase in DA potency (decrease in EC_50_) whereas isomers 3 and 4 increased DA potency by about ∼2.5-fold, which did not achieve statistical significance (Fig. 3A, Table 2). Similar results were seen in the cAMP assay where isomers 1 and 2 increased DA potency by a significant 23-25-fold whereas isomers 3 and 4 were less efficacious, increasing DA potency by 7–11-fold (Fig. 3B, Table 3). Interestingly, in the cAMP assay, isomers 1 and 2 also acted as allosteric agonists (or ago-PAMs) as determined from a partial, but significant 35–40% increase in the baseline cAMP response in the absence of DA (Fig. 3B). This is likely due to high amplification of the G protein-coupled cAMP assay and/or the existence of spare receptors thus rendering this assay more sensitive to low efficacy ligands. In contrast, there is no amplification in the β-arrestin recruitment assay, which does not involve second messenger generation and reflects 1 : 1 complementation of the receptor and β-arrestin. Notably, isomers 3 and 4 were ineffective as agonists in the cAMP assay, which is probably due to their lower efficacy for stabilizing the active state of the receptor in line with their lower efficacy as DA PAMs. While isomer 2 had previously been reported to possess the greatest potentiation among the isomers,^8^ our rigorous stereoisomer characterization shows that isomers 1 and 2 are equally effective PAMs of D_1_R signaling. The *syn* and *anti* isomers are separable by flash chromatography and future researchers may find it pragmatic to simply use racemic *syn* BMS Compound A (mixture of isomers 1 and 2), which is readily accessed using our initial convergent route (Scheme 2) to selectively target the BMS Compound A-specific allosteric site on the D_1_R.

**Table 2:** Potentiation of dopamine-stimulated β-arrestin recruitment by the BMS stereoisomers. Curve parameters were determined as described in Fig. 3A. Data represent the mean ± SEM values from at least 3 experiments. The EC_50_ fold-shifts were derived by dividing the EC_50_ of the DA control curve by the EC_50_ of the DA curve in the presence of the isomers in each experiment. The % *E*_max_ values represent the percent of the control DA *E*_max_ values in each experiment^a^

Condition	EC_50_ (µM)	EC_50_ fold-shift	*E*_max_ (% DA)
DA	1.62 ± 0.26	1.0	100%
+ BMS isomer 1	0.12 ± 0.03***	16.3 ± 2.5**	89.3 ± 1.5%
+ BMS isomer 2	0.20 ± 0.08***	15.1 ± 3.3*	92.9 ± 3.6%
+ BMS isomer 3	0.92 ± 0.18	2.66 ± 0.86	105 ± 3.7%
+ BMS isomer 4	0.94 ± 0.18	2.47 ± 0.70	99.9 ± 3.6%

a Statistical comparisons were made between the control DA EC_50_ values *versus* the DA + BMS isomer EC_50_ values, as well as the EC_50_ fold-shifts, using a one-way ANOVA with Šídák's multiple comparisons test: *p < 0.05, **p < 0.01, ***p < 0.001.

**Table 3:** Potentiation of dopamine-stimulated cAMP accumulation by the BMS stereoisomers. Curve parameters were determined as described in Fig. 3B. Data represent the mean ± SEM values from at least three experiments. The EC_50_ fold-shifts were derived by dividing the EC_50_ of the DA control curve by the EC_50_ of the DA curve in the presence of the isomers in each experiment. The baseline agonism is the percent stimulation of cAMP accumulation in response to 30 µM of each isomer in the absence of DA^a^

Condition	EC_50_ (nM)	EC_50_ fold-shift	Baseline agonism
DA	8.10 ± 3.9	1.0	0%
+ BMS isomer 1	0.25 ± 0.15***	22.8 ± 5.0*	40.2 ± 5.7%*
+ BMS isomer 2	0.18 ± 0.11***	25.8 ± 3.8**	33.8 ± 5.0%*
+ BMS isomer 3	0.86 ± 0.34	11.5 ± 2.4*	4.5 ± 2.8%
+ BMS isomer 4	1.14 ± 0.45*	6.8 ± 1.6*	8.9 ± 5.0%

a Statistical comparisons were made between the control DA EC_50_ values *versus* the DA + BMS isomer EC_50_ values, as well as the EC_50_ fold-shifts, using a one-way ANOVA with Šídák's multiple comparisons test: *p < 0.05, **p < 0.01, ***p < 0.001. Statistical significance of the baseline isomer agonist activities was also evaluated using a one-way ANOVA with Šídák's multiple comparisons test.

**Fig. 4:**
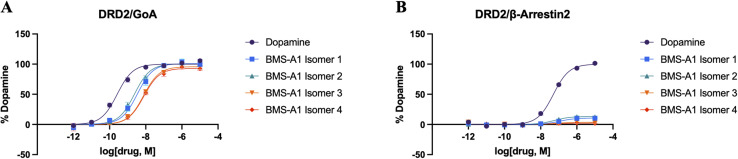
Activation of D_2_R signaling by BMS Compound A isomers using assays for (A) G protein signaling or (B) β-arrestin2 recruitment. The GoA and β-arrestin BRET assays were performed as described in the Experimental Procedures. The data represent the mean of *N* = 3 independent biological replicates with ± SEM. Curve-fit parameters are presented in Tables 4 and 5.

The initial disclosure of BMS Compound A reported that the compound possessed D_2_R agonist activity and we therefore characterized the four stereoisomers for direct activation of D_2_R signaling using TRUPATH,^14^ a bioluminescence resonance energy transfer (BRET)-based assay which measures G protein heterotrimer dissociation (we used GoA, the most abundantly expressed G_i/o_ protein in the brain),^15^ and β-arrestin recruitment (Fig. 4, Tables 4–6). As shown in Fig. 4A, the four stereoisomers possessed similar activity to one another with isomers 3 and 4 being marginally less potent, but statistically significant. This contrasts with their activity as D_1_R PAMs where isomers 1 and 2 were significantly more efficacious than isomers 3 and 4 for increasing the potency of DA signaling. The activities of the four stereoisomers are consistent with D_2_R agonism and suggest that differing structure–activity relationships exist for these compounds at the D_1_ and D_2_ receptors. Interestingly, these results also suggest that the BMS A ligands may be G protein-biased at the D_2_R with statistically insignificant β-arrestin recruitment activity (Fig. 4B). Should these compounds be of interest in studying D_2_R signaling, more detailed investigations will be required to establish their pharmacology and mechanism of action at the D_2_R. Based on the results here, any stereoisomer (or mixture of the four) could serve as a chemical tool in these studies.

**Table 4:** The curve fit parameters for D_2_R signaling by BMS Compound A isomers, as measured by the GoA BRET assays shown in Fig. 4A

	*E*_max_ (% DA) ± SEM	EC_50_ (nM)
DA	100.00 ± 2.81	0.26 ± 0.02
BMS isomer 1	100.59 ± 2.97	3.5 ± 0.35
BMS isomer 2	100.34 ± 2.47	2.4 ± 0.20
BMS isomer 3	95.69 ± 2.25	7.6 ± 0.58
BMS isomer 4	93.08 ± 3.06	7.8 ± 0.81

**Table 5:** The curve fit parameters for D_2_R signaling by BMS Compound A isomers using β-arrestin2 recruitment, as measured by the BRET assays shown in Fig. 4B

	*E*_max_ (% DA) ± SEM	EC_50_ (nM)
DA	100.00 ± 1.89	51 ± 1.80
BMS isomer 1	10.65 ± 1.28	116 ± 30.8
BMS isomer 2	13.23 ± 0.70	57.1 ± 6.18
BMS isomer 3	N.D.	N.D.
BMS isomer 4	N.D.	N.D

**Table 6:** Statistical comparisons of the four stereoisomers in the GoA assay. Comparison of *p*-values from the F-test for the four stereoisomers

DRD2/GoA-pEC50 *p*-value					
	DA	BMS-1	BMS-2	BMS-3	BMS-4
DA		<0.0001	<0.0001	<0.0001	<0.0001
BMS-1			0.139	0.0014	0.0040
BMS-2				<0.0001	<0.0001
BMS-3					0.9130
BMS-4					

## Conclusions

The studies detailed here illustrate the development of two additional efficient synthetic routes to the D_1_R PAM molecule BMS Compound A, a useful chemical tool and benchmark molecule that defines a specific allosteric site on the D_1_R. We have described a short, convergent route to the four previously reported stereoisomers and a modified diastereoselective route to an approximately 1 : 1 mixture of enantiopure diastereomers that are separable using flash chromatography. These routes can be utilized to access any of these four stereoisomers of BMS Compound A without the need for preparative chiral HPLC purification. Our detailed characterization of the four stereoisomers allows for the identification of any individual stereoisomer using only NMR spectroscopy and optical rotation, obviating even chiral HPLC analysis.

Pharmacological evaluation of the four BMS Compound A stereoisomers confirmed that they all exhibit D_1_R PAM activity in both the β-arrestin recruitment and cAMP accumulation assays with the *anti* stereoisomers (isomers 1 and 2) providing more effective potentiation than the *syn* configuration (isomers 3 and 4). In the β-arrestin recruitment assay, isomers 1 and 2 produced a statistically significant ∼15-fold increase in DA potency (decrease in EC_50_) whereas isomers 3 and 4 increased DA potency by about ∼2.5-fold, which did not achieve statistical significance. Similar results were seen in the cAMP assay where isomers 1 and 2 increased DA potency by a significant 23–25-fold whereas isomers 3 and 4 were less efficacious, increasing DA potency by 7–11-fold. Isomers 1 and 2 are equally effective PAMs of D_1_R signaling and researchers may find it pragmatic to simply use racemic *syn* BMS Compound A (mixture of isomers 1 and 2), which is readily accessed using our initial convergent route to selectively target the BMS Compound A-specific allosteric site on the D_1_R. We further characterized the four stereoisomers for direct activation of D_2_R signaling using BRET-based assays to measure G protein heterotrimer dissociation and β-arrestin recruitment. The four stereoisomers possessed similar G protein (GoA) activity to one another with isomers 3 and 4 being marginally less potent, but statistically significant. The results also suggest that the four BMS A ligands may be G protein-biased at the D_2_R with statistically insignificant β-arrestin recruitment activity.

These protocols expand the range of laboratories with the instrumentation to synthesize BMS Compound A or other stereoisomers. The use of chiral ketal-induced diastereoselective reactions on cyclohexane substrates is underutilized and the high selectivity we observed in our initial conditions suggests that this approach should be applicable well beyond the BMS Compound A scaffold or even cyclopropanations. Efforts to harness this diastereoselectivity for the synthesis of other molecules of pharmacological interest or natural product targets is currently under investigation in our labs.

## Experimental

### General experimental information

All reagents and solvents were purchased from commercial suppliers and used without further purification. No unanticipated safety hazards were encountered during any of these chemical syntheses. All reactions should be performed in a well-ventilated fume hood and personal protective equipment worn when performing reactions or handling compounds. Temperatures are reported in degrees Celsius (°C). Where not noted, reactions were run at room temperature (25 °C). Unless otherwise noted, reactions were carried out under an argon atmosphere. Upon completion, reactions were worked up, where necessary, and solvent was removed *via* rotary evaporator under reduced pressure. Flash column chromatography separations were performed on the Teledyne Isco CombiFlash Rf automated liquid flash chromatography system using pre-loaded silica cartridges. The following abbreviations are used in schemes and/or experimental procedures: acetic acid (AcOH), dichloromethane (DCM), dimethylethylamine (DMEA) dimethylsulfoxide (DMSO), equiv. (equivalent(s)), ethyl acetate (EtOAc), isopropanol (*i*-PrOH), hours (h), micromoles (µmol), milligrams (mg), millimoles (mmol), minutes (min), room temperature (rt), supercritical fluid chromatography (SFC) and tetrahydrofuran (THF). ^1^H NMR data were recorded on a 400 MHz Bruker Avance spectrometer equipped with a broadband observe probe and a 500 MHz Bruker AVIII spectrometer equipped with a dual cryoprobe, respectively. ^1^H and ^13^C NMR spectra were obtained in CDCl_3_. The spectrometer magnet strength is indicated for each experiment. Chemical shifts, which have been calibrated *versus* the shift of the deuterated solvent, are reported in parts per million (ppm). Coupling constants (*J* values) are reported in hertz (Hz) and spin multiplicities are listed as follows: singlet (s), doublet (d), doublet of doublets/triplets/quartets (dd/dt/dq), triplet (t), triplet of doublets (td), quartet (q), and multiplet (m). HRMS samples were analyzed by the UNC Department of Chemistry Mass Spectrometry Core Laboratory using a Q Exactive HF-X mass spectrometer. Optical rotation was measured using a 1 mL cell with 2.0 dm path length on an Autopol® IV automatic polarimeter. Preparative chiral HPLC purification was carried out by Chiral Technologies Inc. at rt on a Chiralpak ADH column (30 × 250 mm) eluting with a 98 : 2 hexanes:ethanol solvent system with 0.1% diethylamine at a flow rate of 40 mL min^−1^. Analytical chiral HPLC was carried out either by Chiral Technologies Inc. using a Chiralpak ADH column (4.6 × 250 mm) under analogous conditions or in our laboratory on an Agilent 1260 Infinity II SFC–HPLC at rt using a Chiralpak IC column (4.6 × 250 mm, 5 micron) eluting with a 60 : 40 hexanes : methanol solvent system at a flowrate of 1 mL min^−1^.

### β-Arrestin recruitment assay

Agonist-mediated recruitment of β-arrestin-2 to all five dopamine receptor subtypes was determined using the DiscoverX PathHunter complementation assay (DiscoverX Inc., Fremont, CA), as previously described by our laboratory.^7^ Briefly, CHO-K1 cells stably expressing the human D_1_R (obtained from DiscoverX Inc.) were seeded in cell plating (CP) media (DiscoverX) at a density of 2625 cells per well and 7.5 µL per well in 384-well black, clear-bottom plates. Following 18–24 h of incubation, the cells were treated with the indicated concentrations of dopamine +/− PAMs in PBS buffer containing <2% DMSO and 0.2 mM sodium metabisulfite and incubated at 37 °C for 90 min. DiscoverX reagent was added to cells according to the manufacturer's protocol, followed by a 60 min incubation in the dark at room temperature. Luminescence was measured on a Hamamatsu FDSS µ-Cell reader (Hamamatsu, Bridgewater, NJ) and data were collected using the FDSS software. Data were collected as relative luminescence units (RLUs) and normalized to a percentage of the control luminescence seen with a maximum concentration of dopamine with zero percent (baseline) being RLUs produced in the absence of any compound.

### Lance assay for cAMP

cAMP accumulation was measured by using the time-resolved fluorescence energy transfer-based LANCE cAMP assay (PerkinElmer) and HEK293 cells (obtained from ATCC, CRL number 1573). Briefly, 4 × 10^6^ HEK293 cells per plate were seeded on 100 mm dishes and incubated overnight. Cells were then transfected with 5 µg of plasmid encoding human D_1_R using the PEI method. 16 h later the media was replaced for fresh media. 48 hours after transfection cells were harvested, washed and resuspended in HBSS containing 200 µM sodium metabisulfite and 20 µM HEPES, and were plated in 384-well white plates at 1 × 10^6^ cells per mL and 5 µL per well. Immediately after plating, cells were treated with 5 µL of varying concentrations of dopamine +/− PAMs and incubated for 30 min at rt. Following incubation, 5 µL of Tracer and 5 µL of a-cAMP were added to each well according to the manufacturer's protocol and cells were incubated in the dark for 2 hours at rt. Plate was then read on PheraSTAR plate reader (BMG Labtech, Cary, NC) with excitation at 337 nm and emission at 620 nm and 665 nm. Data are obtained as the ratio between A (excitation at 337 nm/emission at 665 nm) and B (excitation at 337 nm/emission at 620 nm).

### D_2_R TRUPATH and β-arrestin 2 BRET assays

TRUPATH and β-Arrestin 2 BRET assays were carried out similar to prior work.^14,16^ In brief, HEK293T cells (obtained from ATCC *via* the UNC Tissue Culture Facility, CRL number 11268) were transfected with a 1 : 1 : 1 : 1 ratio of receptor : Gα: Gβ : Gγ or a 1 : 1 : 1 ratio of receptor-RLuc8 : GRK2 : GFP2-β-Arrestin2 using Transit 2020 (Mirus Biosciences). The following day, complete media (DMEM, 10% FBS, 1× penicillin-streptomycin) was replaced with dialyzed media (DMEM, 1% dialyzed FBS, 1× penicillin-streptomycin). After at least 3 hours, transfected cells were detached with Versene (1× PBS, 0.5 mM EDTA, pH 7.4) and plated in 96-well plates at a density of 30 000–50,000 cells/well in dialyzed media. Following an overnight incubation, dialyzed media was replaced with 60 µL of assay buffer (1× HBSS, 20 mM HEPES, pH 7.4). After 10 min, 30 µL of drug dissolved in buffer (assay buffer supplemented with 0.6% BSA, 3 mM ascorbate) was added to each well and incubated at 37 °C for 10 minutes. Finally, 10 µL of 50 µM coelenterazine 400a was added to each well and incubated for 10 minutes at room temperature. Plates were read using the PHERAstar FSX detection system for a total of 5 reads. BRET ratios were calculated using 395 nm/510 nm emission from the final read. CRCs were plotted and analyzed using GraphPad Prism nonlinear regression: log(agonist) *vs.* response (three parameters).

### Synthesis protocols and characterization

#### 8-(Benzo[*d*][1,3]dioxol-5-yl)-1,4-dioxaspiro[4.5]dec-7-ene 3

4,4,5,5-Tetramethyl-2-(1,4-dioxaspiro[4.5]dec-7-en-8-yl)-1,3,2-dioxaborolane 2 (3.998 g, 1.5 equiv., 15.02 mmol), 5-bromobenzo[*d*][1,3]dioxole 1 (2.013 g, 1.0 equiv., 10.02 mmol), sodium carbonate (2.123 g, 2.0 equiv., 20.03 mmol) and tetrakis (triphenylphosphino)palladium (578.6 mg, 0.05 equiv., 500.8 µmol) were combined in a round-bottom flask (50 mL). The atmosphere was removed under vacuum and replaced with argon. The solvents 1,4-dioxane (30 mL) and water (3 mL) were added and the reaction bubbled with argon for 5 minutes. The reaction mixture was heated to 80 °C under argon for 16 h. After cooling to rt, the reaction was concentrated to remove 1,4-dioxane, diluted with water (30 mL), and extracted with ethyl acetate (2 × 50 mL). The combined organic layers were washed with brine, dried over Na_2_SO_4_, filtered, and concentrated under vacuum. The crude compound was purified by flash chromatography on silica gel (EtOAc/hexane: 0 to 15%) to give 8-(benzo[*d*][1,3]dioxol-5-yl)-1,4-dioxaspiro[4.5]dec-7-ene 3 (2.089 g, 8.026 mmol, 80% yield). ^1^H NMR (400 MHz, CDCl_3_) *δ* 1.90 (t, *J* = 6.5 Hz, 2H), 2.44 (d, *J* = 3.7 Hz, 2H), 2.60 (ddq, *J* = 6.4, 3.8, 1.9 Hz, 2H), 4.02 (s, 4H), 5.87 (td, *J* = 4.0, 2.0 Hz, 1H), 5.94 (s, 2H), 6.74 (d, *J* = 8.1 Hz, 1H), 6.86 (dd, *J* = 8.1, 1.8 Hz, 1H), 6.90 (d, *J* = 1.8 Hz, 1H). These data are in agreement with those previously reported.^17^

#### 6-(Benzo[*d*][1,3]dioxol-5-yl)bicyclo[4.1.0]heptan-3-one 5

A solution of trifluoroacetic acid (440.9 mg, 0.5 equiv., 3.867 mmol) was slowly added to the solution of diethyl zinc (24 mL, 1 M in toluene, 3.0 equiv., 24.0 mmol—caution, diethyl zinc is potentially pyrophoric and may ignite upon contact with air; use inert atmosphere transfer techniques when handling) in dry DCM (50 mL) at 0 °C, and stirred at 0 °C for 30 minutes. Diiodomethane (10.3560 g, 5.0 equiv., 38.665 mmol) was slowly added, and a solution of 8-(benzo[*d*][1,3]dioxol-5-yl)-1,4-dioxaspiro[4.5]dec-7-ene 3 (2.013 g, 1.0 equiv., 7.733 mmol) in dry DCM (6 mL) was slowly added over 5 min. The reaction mixture was warmed to rt, and stirred for 5 h. An aqueous HCl solution (1 *N*) was slowly added to quench the reaction, and the reaction concentrated under reduced pressure to afford the crude cyclopropane 4, which was used without further purification. Acetone (50 mL) and aqueous HCl solution (2 N, 40 mL) were added to the resulting residue, which was then heated at 40 °C for 13 h. The solvent was removed under reduced pressure and the residue purified by flash chromatography (EtOAc/hexane: 0 to 40%) to give 6-(benzo[*d*][1,3]dioxol-5-yl)bicyclo[4.1.0]heptan-3-one 5 (1.349 g, 5.807 mmol, 75% yield). ^1^H NMR (400 MHz, CDCl_3_) *δ* 0.94 (t, *J* = 5.5 Hz, 1H), 1.02 (dd, *J* = 9.0, 5.7 Hz, 1H), 1.43 (ddd, *J* = 13.8, 5.1, 2.5 Hz, 1H), 2.14–2.27 (m, 2H), 2.31–2.47 (m, 2H), 2.66 (dd, *J* = 18.5, 2.5 Hz, 1H), 2.85 (dd, *J* = 18.5, 5.0 Hz, 1H), 5.93 (s, 2H), 6.73–6.82 (m, 3H). ^13^C NMR (126 MHz, CDCl_3_) *δ* 15.0, 17.1, 24.1, 28.7, 36.7, 39.0, 100.9, 108.1, 108.4, 120.6, 140.1, 145.9, 147.6, 210.9.

#### *tert*-Butyl 4-(2-bromo-5-chlorobenzyl)piperazine-1-carboxylate 7

A solution of 2-bromo-5-chlorobenzaldehyde 6 (2.191 g, 1.0 equiv., 9.983 mmol), *tert*-butyl piperazine-1-carboxylate (5.578 g, 3.0 equiv., 29.95 mmol), NaBH(OAc)_3_ (6.347 g, 3.0 equiv., 29.95 mmol) and AcOH (3 drops) in DCM (50 mL) was refluxed for 16 h. The reaction solution was cooled to rt, aqueous Na_2_CO_3_ (sat.) was added, and the reaction extracted with DCM. The combined organic layers were dried with Na_2_SO_4_ and concentrated under reduced pressure. The residue was purified by flash chromatography (EtOAc/hexane: 0 to 20%) to give *tert*-butyl 4-(2-bromo-5-chlorobenzyl)piperazine-1-carboxylate 7 (3.224 g, 8.273 mmol, 83% yield). ^1^H NMR (400 MHz, CDCl_3_) *δ* 1.46 (s, 9H), 2.46 (br s, 4H), 3.39–3.51 (m, 4H), 3.56 (s, 2H), 7.10 (dd, *J* = 8.5, 2.4 Hz, 1H), 7.46 (d, *J* = 8.5 Hz, 1H), 7.49 (s, 1H). ^13^C NMR (126 MHz, CDCl_3_) *δ* 28.4, 44.1, 52.9, 61.4, 79.7, 122.2, 128.6, 130.4, 133.5, 133.8, 139.3, 154.7. HRMS (ESI) *m*/*z*: calcd for C_16_H_23_BrClN_2_O_2_: 389.0631 [M + H]^+^; found: 389.0631.

#### 1-(6-(Benzo[*d*][1,3]dioxol-5-yl)bicyclo[4.1.0]heptan-3-yl)-4-(2-bromo-5-chlorobenzyl)piperazine BMS Compound A (four stereoisomers)

A solution of trifluoroacetic acid (8 mL) in DCM (8 mL) was slowed added to a solution of *tert*-butyl 4-(2-bromo-5-chlorobenzyl)piperazine-1-carboxylate (3.104 g, 1.5 equiv., 7.964 mmol) and triethylsilane (1.235 g, 2.0 equiv., 10.62 mmol) in DCM (4 mL) and stirred for 2 h at rt. The reaction mixture was concentrated under reduced pressure, the residue dissolved in CHCl_3_ (20 mL), and 6-(benzo[*d*][1,3]dioxol-5-yl)bicyclo[4.1.0]heptan-3-one 5 (1.223 g, 1.0 equiv., 5.310 mmol) was added. To this reaction solution, NaBH(OAc)_3_ (3.376 g, 3.0 equiv., 15.93 mmol) was added and the reaction was refluxed for 16 h. The reaction solution was cooled to rt, aqueous Na_2_CO_3_ (sat.) was added, and the reaction mixture extracted with DCM. The combined organic layers were dried with Na_2_SO_4_ and concentrated under reduced pressure. The residue was purified by flash chromatography (EtOAc/hexane: 0 to 20%) to give *syn*-BMS Compound A (0.8811 g, 1.749 mmol, 33% yield) and *anti*-BMS Compound A (0.9638 g, 1.913 mmol, 36% yield).

#### (±)-*syn*-BMS Compound A

^1^H NMR (400 MHz, CDCl_3_) *δ* 0.63 (t, *J* = 5.0 Hz, 1H), 0.88 (dd, *J* = 9.3, 4.5 Hz, 1H), 1.01–1.30 (m, 2H), 1.48–1.60 (m, 1H), 1.70–1.81 (m, 1H), 1.85–2.07 (m, 1H), 2.23 (dt, *J* = 13.3, 3.3 Hz, 1H), 2.34 (td, *J* = 10.9, 6.3 Hz, 2H), 2.51–2.62 (m, 8H), 3.55 (s, 2H), 5.90 (s, 2H), 6.66–6.79 (m, 4H), 7.08 (dd, *J* = 8.5, 2.6 Hz, 1H), 7.44 (d, *J* = 8.5 Hz, 1H), 7.48 (d, *J* = 2.6 Hz, 1H). These data are in agreement with those previously reported.^9^

#### (±)-*anti*-BMS Compound A

^1^H NMR (400 MHz, CDCl_3_) *δ* 0.44–0.57 (m, 1H), 0.95 (dd, *J* = 9.4, 4.5 Hz, 1H), 1.19–1.32 (m, 1H), 1.33–1.43 (m, 1H), 1.54–1.67 (m, 1H), 1.79–2.07 (m, 3H), 2.15–2.23 (m, 2H), 2.59 (s, 8H), 3.56 (s, 2H), 5.90 (s, 2H), 6.65–6.77 (m, 2H), 6.80 (d, *J* = 1.5 Hz, 1H), 7.08 (dd, *J* = 8.5, 2.6 Hz, 1H), 7.45 (d, *J* = 8.5 Hz, 1H), 7.49 (d, *J* = 2.6 Hz, 1H). These data are in agreement with those previously reported.^9^

A mixture of (±)-*syn*-BMS Compound A and (±)-*anti*-BMS Compound A (490.7 mg) was separated by preparative, chiral HPLC as described in the general experimental section to afford the four stereoisomers of BMS Compound A: isomer 1 (44.5 mg, 97.8% purity); [*α*]^20^_D_ = +27.0 (*c* 0.50, CHCl_3_), isomer 2 (40.9 mg, 91.2% purity); isomer 3 (67.4 mg, 98.6% purity); [*α*]^20^_D_ = +25.0 (*c* 0.50, CHCl_3_) and isomer 4 (68.6 mg, 93.9% purity);

### X-ray crystallography of BMS Compound A: isomer 1

Isomer 1 (25 mg) was dissolved in THF (4 mL) and HCl (1 M in ethyl acetate, 1 mL) was added precipitating a fluffy hydrochloride salt. The solvents were removed under reduced pressure and the residue recrystallized from methanol/ethanol/water to afford crystals suitable for X-ray diffraction. A colorless crystal (approximate dimensions 0.150 × 0.050 × 0.010 mm^3^) was placed onto the tip of MiTeGen and mounted on a Bruker D8 VENTURE diffractometer and measured at 150 K.

### Data collection

A preliminary set of cell constants was calculated from reflections harvested from a set of 180 frames. These initial sets of frames were oriented such half a sphere in the reciprocal space was surveyed. This produced initial orientation matrices determined from 826 reflections. The data collection was carried out using Cu Kα radiation (graphite monochromator) with a theta dependent frame time window of 1.13 (attenuated) to 5 seconds and a detector distance of 3.7 cm. A randomly oriented region of reciprocal space was surveyed to achieve complete data with a redundancy of 6.3. Sections of frames were collected with 1.50° steps in *ω* and *ϕ* scans. Data to a resolution of 0.82 Å were considered in the reduction. Final cell constants were calculated from the xyz centroids of 9820 strong reflections from the actual data collection after integration (SAINT, Bruker Analytical X-ray Systems). The intensity data were corrected for absorption (SADABS).^18^ Please refer to Table S1 for additional crystal and refinement information.

Structure solution and refinement: The space group P 1 21 1 was determined based on intensity statistics and systematic absences. The structure was solved using Superflip^19^ and refined (full-matrix-least squares) using the Oxford University Crystals for Windows system.^20^ The charge-flipping solution provided most non-hydrogen atoms from the E-map. Full-matrix least squares/difference Fourier cycles were performed, which located the remaining non-hydrogen atoms. All non-hydrogen atoms were refined with anisotropic displacement parameters. The hydrogen atoms were placed in ideal positions and refined as riding atoms. The final full matrix least squares refinement converged to *R*1 = 0.0276 and w*R*2 = 0.0713 (F2, all data).

### Structure description

The structure was found as proposed with an additional protonation of a nitrogen, charge balanced by a chloride ion. Flack parameter was calculated to be −0.015(13).

### X-ray crystallography of BMS Compound A: isomer 4

Isomer 4 (25 mg) was dissolved in THF (4 mL) and HCl (1 M in EtOAc, 1 mL) was added precipitating a fluffy hydrochloride salt. The solvents were removed under reduced pressure and the residue recrystallized from from ethanol/ethyl acetate to afford crystals suitable for X-ray diffraction. A colorless crystal (approximate dimensions 0.040 × 0.040 × 0.010 mm^3^) was placed onto the tip of MiTeGen, mounted on a Bruker D8 VENTURE diffractometer, and measured at 150 K.

### Data collection

A preliminary set of cell constants was calculated from reflections harvested from a set of 180 frames. These initial sets of frames were oriented such half a sphere in the reciprocal space was surveyed. This produced initial orientation matrices determined from 1058 reflections. The data collection was carried out using Cu Kα radiation (graphite monochromator) with theta-dependent frame window of 1 to 8 seconds and a detector distance of 3.7 cm. A randomly oriented region of reciprocal space was surveyed to achieve complete data with a redundancy of 11.5. Sections of frames were collected with 0.40° steps in *ω* and *ϕ* scans. Data to a resolution of 0.82 Å were considered in the reduction. Final cell constants were calculated from the xyz centroids of 9079 strong reflections from the actual data collection after integration (SAINT, Bruker Analytical X-ray Systems). The intensity data were corrected for absorption (SADABS).^14^ Please refer to Table S2 for additional crystal and refinement information.

### Structure solution and refinement

Determining the space group for structure 24 201 was challenging due to crystal quality and a centrosymmetric-like packing, despite the compound was confirmed as enantiopure based on chiral HPLC analysis. Initially, the centrosymmetric space group *P*2_1_/*c* was considered, but this led to a 50/50 disorder on the chiral carbon of the bicyclo[4.1.0]heptane. In contrast, the space group *P*2_1_ showed no disorder, suggesting it was the correct choice. In the *P*2_1_ solution, the two independent molecules were largely related by a center of inversion, except for the 1C bridge on the bicycloheptane, which may have contributed to a Flack parameter close to 0.5. The structure was solved using SHELXT^21^ and refined (full-matrix-least squares) using the Oxford University Crystals for Windows system.^16^ The intrinsic solution provided most non-hydrogen atoms from the E-map. Full-matrix least squares/difference Fourier cycles were performed, which located the remaining non-hydrogen atoms. All non-hydrogen atoms were refined with anisotropic displacement parameters. Hydrogen atoms on parent nitrogen atoms were obtained using the difference map and refined with appropriate bond length restraints, while other hydrogen atoms were placed in ideal positions and refined as riding atoms. The final full matrix least squares refinement converged to *R*1 = 0.0737 and w*R*2 = 0.2056 (F2, all data).

### Structure description

The structure was found as proposed. Flack parameter was calculated to be 0.326(12).

### 4-(Benzo[*d*][1,3]dioxol-5-yl)cyclohex-3-en-1-one 8

4,4,5,5-Tetramethyl-2-(1,4-dioxaspiro[4.5]dec-7-en-8-yl)-1,3,2-dioxaborolane 2 (4.000 g, 1.5 equiv., 15.03 mmol), 5-bromobenzo[*d*][1,3]dioxole 1 (2.014 g, 1.0 equiv., 10.02 mmol), sodium carbonate (2.124 g, 2.0 equiv., 20.04 mmol) and tetrakis (triphenylphosphino)palladium (578.9 mg, 0.05 equiv., 501.0 µmol) were combined in a round-bottom flask (50 mL). The atmosphere was removed under vacuum and replaced with argon. The solvents 1,4-dioxane (30 mL) and water (3 mL) were added and the reaction was bubbled with argon for a few minutes. Then, the reaction was heated to 100 °C under argon for 24 h. After cooling to rt, the reaction mixture was filtered through a pad of Celite which was further washed with EtOAc, and the combined filtrates were concentrated under reduced pressure. The residue was dissolved in acetone (50 mL) and aqueous HCl solution (2 N, 40 mL) was added. The reaction mixture was refluxed for 13 h and the solvents removed under reduced pressure. The residue was purified by flash chromatography (EtOAc/hexane: 0 to 20%) to give 4-(benzo[*d*][1,3]dioxol-5-yl)cyclohex-3-en-1-one 8 (1.720 g, 7.955 mmol, 79% yield). ^1^H NMR (400 MHz, CDCl_3_) 2.63 (t, *J* = 6.9 Hz, 2H), 2.84 (dt, *J* = 6.8, 3.4 Hz, 2H), 3.05 (dt, *J* = 3.7, 1.9 Hz, 2H), 5.97 (s, 2H), 5.95–6.00 (m, 1H), 6.79 (d, *J* = 8.0 Hz, 1H), 6.86 (dd, *J* = 8.1, 1.8 Hz, 1H), 6.90 (d, *J* = 1.7 Hz, 1H). ^13^C NMR (126 MHz, CDCl3) *δ* 28.1, 38.7, 39.9, 101.1, 105.9, 108.1, 118.7, 119.9, 135.2, 137.3, 147.0, 147.8, 210.1.

### (2*R*,3*R*)-8-(Benzo[*d*][1,3]dioxol-5-yl)-2,3-diphenyl-1,4-dioxaspiro[4.5]dec-7-ene 9

A mixture of 4-(benzo[*d*][1,3]dioxol-5-yl)cyclohex-3-en-1-one 8 (433.7 mg, 1.0 equiv., 2.006 mmol), pyridinium *p*-toluenesulfonate (100.8 mg, 0.2 equiv., 0.401 mmol) and (1*R*,2*R*)-1,2-diphenylethane-1,2-diol (472.7 mg, 1.1 equiv., 2.206 mmol, 99% ee) in toluene (5 mL) was refluxed for 12 h and the solvent was removed under reduced pressure. The residue was purified by flash chromatography (EtOAc/hexane: 0 to 40%) and recrystallized from DCM/hexane to give (2*R*,3*R*)-8-(benzo[*d*][1,3]dioxol-5-yl)-2,3-diphenyl-1,4-dioxaspiro[4.5]dec-7-ene 9 (1.399 g, 3.392 mmol, 78% yield, 99% ee). [*α*]^20^_D_ = 42.4 (*c* 1.0, CHCl_3_). ^1^H NMR (400 MHz, CDCl_3_) *δ* 2.11–2.34 (m, 2H), 2.65–2.87 (m, 4H), 4.66 (d, *J* = 8.6 Hz, 1H), 4.69 (d, *J* = 8.5 Hz, 1H), 5.81 (d, *J* = 1.5 Hz, 1H), 5.82 (d, *J* = 1.4 Hz, 1H), 5.92–5.99 (m, 1H), 6.75 (d, *J* = 8.1 Hz, 1H), 6.89 (dd, *J* = 8.1, 1.8 Hz, 1H), 6.93 (d, *J* = 1.7 Hz, 1H), 7.20–7.37 (m, 10H). ^13^C NMR (126 MHz, CDCl3) *δ* 27.0, 32.9, 37.5, 85.3, 85.4, 100.9, 105.9, 107.9, 108.6, 118.6, 120.5, 126.7, 126.8, 128.3, 128.3, 128.4, 128.4, 135.8, 136.0, 136.6, 136.9, 146.5, 147.6. HRMS (APCI) *m*/*z*: calcd for C_27_H_25_O_4_: 413.1753 [M + H]^+^; found: 413.1752. SFC-HPLC conditions: Chiralpak IC column, flow rate = 3.0 mL min; 35 °C; CO_2_/*i*-PrOH = 95/5.

### (1*S*,4′*R*,5′*R*,6*R*)-6-(Benzo[*d*][1,3]dioxol-5-yl)-4′,5′-diphenylspiro[bicyclo[4.1.0]heptane-3,2'-[1,3]dioxolane] 10

A solution of trifluoroacetic acid (189.6 mg, 0.5 equiv., 1.663 mmol) was slowly added to the solution of diethyl zinc (10 mL, 1 M in toluene, 3.0 equiv., 10.0 mmol) in dry DCM (50 mL) at −15 °C, and stirred at −15 °C for 30 minutes. Diiodomethane (4.455 g, 5.0 equiv., 16.63 mmol) was slowly added, and a solution of (2*R*,3*R*)-8-(benzo[*d*][1,3]dioxol-5-yl)-2,3-diphenyl-1,4-dioxaspiro[4.5]dec-7-ene (1.372 g, 1.0 equiv., 3.327 mmol) in dry DCM (6 mL) was slowly added over 5 min. The reaction mixture was stirred −15 °C for 10 h, and quenched by saturated aqueous ammonium chloride solution. The reaction solution was filtered through Celite and washed with DCM. The filtrate was concentrated under reduced pressure and purified by C18 flash chromatography (CH_3_CN/water with 0.1% formic acid: 90 to 0%) followed by flash chromatography (EtOAc/hexane: 0 to 15%) to give (1*S*,4′*R*,5′*R*,6*R*)-6-(benzo[*d*][1,3]dioxol-5-yl)-4′,5′-diphenylspiro[bicyclo[4.1.0]heptane-3,2'-[1,3]dioxolane] 10 (101.5 mg, 0.238 mmol, 71% yield, 99% ee). [*α*]^20^_D_ = +35.1 (*c* 0.50, CHCl_3_). ^1^H NMR (400 MHz, CDCl_3_) *δ* 0.74 (t, *J* = 5.2 Hz, 1H), 0.97 (dd, *J* = 9.3, 4.6 Hz, 1H), 1.21–1.37 (m, 2H), 1.70 (ddd, *J* = 13.5, 10.4, 5.2 Hz, 1H), 1.86–1.94 (m, 1H), 2.05 (dd, *J* = 14.4, 1.3 Hz, 1H), 2.17 (dt, *J* = 13.8, 5.4 Hz, 1H), 2.29 (ddd, *J* = 13.9, 10.4, 5.6 Hz, 1H), 2.50 (ddd, *J* = 14.3, 8.1, 1.7 Hz, 1H), 4.66 (d, *J* = 8.6 Hz, 1H), 4.69 (d, *J* = 8.5 Hz, 1H), 5.81 (d, *J* = 1.5 Hz, 1H), 5.82 (d, *J* = 1.4 Hz, 1H), 6.63 (d, *J* = 8.0 Hz, 1H), 6.73 (dd, *J* = 8.0, 1.7 Hz, 1H), 6.78 (d, *J* = 1.7 Hz, 1H), 7.12–7.32 (m, 10H). ^13^C NMR (126 MHz, CDCl_3_) *δ* 17.7, 18.6, 24.4, 30.5, 31.7, 35.5, 84.9, 85.5, 100.7, 107.9, 108.9, 109.1, 120.8, 126.6, 126.7, 128.16, 128.17, 128.37, 128.38, 137.11, 137.14, 142.7, 145.5, 147.3. HRMS (ESI) *m*/*z*: calcd for C_28_H_27_O_4_: 427.1909 [M + H]^+^; found: 427.1910. SFC-HPLC conditions: Chiralpak IC column, flow rate = 3.0 mL min; 35 °C; CO_2_/*i*-PrOH = 95/5.

### (+)-(1*S*,6*R*)-6-(Benzo[*d*][1,3]dioxol-5-yl)bicyclo[4.1.0]heptan-3-one (+)-5

A mixture of (1*S*,4′*R*,5′*R*,6*R*)-6-(benzo[*d*][1,3]dioxol-5-yl)-4′,5′-diphenylspiro[bicyclo[4.1.0]heptane-3,2'-[1,3]dioxolane] 10 (101.5 mg, 1.0 equiv., 0.238 mmol) and aqueous HCl solution (2 N, 3 mL) in acetone (10 mL) was refluxed for 13 h and the solvent was removed under reduced pressure. The residue was purified by flash chromatography (EtOAc/hexane: 0 to 40%) to give (1*S*,6*R*)-6-(benzo[*d*][1,3]dioxol-5-yl)bicyclo[4.1.0]heptan-3-one (+)-5 (52.0 mg, 0.226 mmol, 95% yield) with identical spectroscopic characterization to racemic 5. [*α*]^20^_D_ = +169.3 (*c* 1.00, CHCl_3_).

### 1-(6-((1*S*,3*R*,6*R*)-Benzo[*d*][1,3]dioxol-5-yl)bicyclo[4.1.0]heptan-3-yl)-4-(2-bromo-5-chlorobenzyl)piperazine BMS Compound A (isomer 2) and 1-(6-((1*S*,3*S*,6*R*)-benzo[*d*][1,3]dioxol-5-yl)bicyclo[4.1.0]heptan-3-yl)-4-(2-bromo-5-chlorobenzyl)piperazine BMS Compound A (isomer 4)

A solution of *tert*-butyl 4-(2-bromo-5-chlorobenzyl)piperazine-1-carboxylate (130.8 mg, 1.5 equiv., 0.336 mmol) and triethylsilane (50.8 mg, 2.0 equiv., 0.437 mmol) in DCM (2 mL) was treated with trifluoroacetic acid (1 mL) and DCM (1 mL), and stirred at rt for 5 h. The solvent was removed under reduced pressure, and the residue was dissolved in CHCl_3_ (5 mL). (1*S*,6*R*)-6-(Benzo[*d*][1,3]dioxol-5-yl)bicyclo[4.1.0]heptan-3-one (+)-5 (50.1 mg, 1.0 equiv., 0.218 mmol) and NaBH(OAc)_3_ (138.3 mg, 3.0 equiv., 0.653 mmol) were added, and the reaction was refluxed for 16 h. The reaction solution was cooled to rt, and aqueous Na_2_CO_3_ (sat.) was added. The reaction mixture was extracted with DCM, dried with Na_2_SO_4_, and concentrated under reduced pressure. The residue was purified by flash chromatography (EtOAc/hexane: 0 to 20%) to give (1*S*,3*R*,6*R*)-BMS Compound A (34.1 mg, 67.7 µmol, 31% yield) and (1*S*,3*S*,6*R*)-BMS Compound A (37.9 mg, 75.2 µmol, 35% yield).

### (1*S*,3*R*,6*R*)-BMS Compound A

^1^H NMR (400 MHz, CDCl_3_) *δ* 0.44–0.57 (m, 1H), 0.95 (dd, *J* = 9.4, 4.5 Hz, 1H), 1.19–1.32 (m, 1H), 1.33–1.43 (m, 1H), 1.54–1.67 (m, 1H), 1.79–2.07 (m, 3H), 2.15–2.23 (m, 2H), 2.59 (s, 8H), 3.56 (s, 2H), 5.90 (s, 2H), 6.65–6.77 (m, 2H), 6.80 (d, *J* = 1.5 Hz, 1H), 7.08 (dd, *J* = 8.5, 2.6 Hz, 1H), 7.45 (d, *J* = 8.5 Hz, 1H), 7.49 (d, *J* = 2.6 Hz, 1H). ^13^C NMR (126 MHz, CDCl_3_) *δ* 18.7, 20.5, 24.1, 26.0, 26.1, 32.7, 49.2, 53.5, 58.1, 61.4, 100.7, 107.8, 108.5, 120.2, 122.1, 128.3, 130.3, 133.4, 133.7, 139.6, 143.7, 145.3, 147.3. Enantiomeric ratio (isomer 2:isomer 1) determined by SFC-HPLC to be 99 : 1. SFC-HPLC conditions: Chiralpak IC column, flow rate = 2.0 mL, 35 °C, CO_2_/*i*-PrOH with 0.1% DMEA: 80/20. [*α*]^20^_D_ = −27.2 (*c* 0.50, CHCl_3_).

### (1*S*,3*S*,6*R*)-BMS Compound A

^1^H NMR (400 MHz, CDCl_3_) *δ* 0.63 (t, *J* = 5.0 Hz, 1H), 0.88 (dd, *J* = 9.3, 4.5 Hz, 1H), 1.01–1.30 (m, 2H), 1.48–1.60 (m, 1H), 1.70–1.81 (m, 1H), 1.85–2.07 (m, 1H), 2.23 (dt, *J* = 13.3, 3.3 Hz, 1H), 2.34 (td, *J* = 10.9, 6.3 Hz, 2H), 2.51–2.62 (m, 8H), 3.55 (s, 2H), 5.90 (s, 2H), 6.66–6.79 (m, 4H), 7.08 (dd, *J* = 8.5, 2.6 Hz, 1H), 7.44 (d, *J* = 8.5 Hz, 1H), 7.48 (d, *J* = 2.6 Hz, 1H). ^13^C NMR (126 MHz, CDCl_3_) *δ* 18.09, 18.12, 23.6, 25.2, 26.8, 32.2, 49.0, 53.3, 60.9, 61.4, 100.8, 107.9, 108.5, 120.5, 122.1, 128.4, 130.3, 133.4, 133.7, 139.5, 142.5, 145.4, 147.4. Enantiomeric ratio (isomer 4:isomer 3) determined by SFC-HPLC to be 99 : 1. SFC-HPLC conditions: Chiralpak IG column, flow rate = 2.5 mL, 35 °C, CO_2_/*i*-PrOH with 0.1% DMEA: 80/20. [*α*]^20^_D_ = −25.8 (*c* 0.50, CHCl_3_).

## Author contributions

Feijun Wang: investigation, methodology and formal analysis. John N. Hanson: investigation, formal analysis and writing – review and editing. Snezana T. Dimova: investigation and formal analysis. Jayachandra Rayadurgam: investigation and formal analysis. Camryn J. Fulton: investigation and formal analysis. Amy E. Moritz: investigation and formal analysis. Ashley N. Nilson: investigation and formal analysis. William A. Hearne: investigation and formal analysis. Chun-Hsing Chen: supervision and formal analysis. Ryan H. Gumpper: supervision, formal analysis and writing – review and editing. David R. Sibley: conceptualization, project administration, funding acquisition, supervision, visualization and writing – original draft. Kevin J. Frankowski: conceptualization, project administration, funding acquisition, supervision, visualization and writing – original draft.

## Conflicts of interest

The authors have no conflicts of interest.

## Supplementary Material

RA-016-D6RA02434C-s001

RA-016-D6RA02434C-s002

RA-016-D6RA02434C-s003

## Data Availability

The data supporting this article have been included as part of the supplementary information (SI). Structural data can be accessed at https://www.ccdc.cam.ac.uk. CCDC 2540023 and 2540093 contain the supplementary crystallographic data for this paper.^22^ Supplementary information: concentration-response curves of BMS Compound A isomers for potentiating dopamine-stimulated β-arrestin recruitment to the D_1_R, NMR spectra for all synthesized compounds, chiral HPLC chromatograms and X-ray crystal data and structure refinement details. Raw NMR data files for BMS Compound A isomers 2 and 4 (representative diastereomers) to allow independent analysis of the ^1^H and ^13^C NMR spectra of these molecules. See DOI: https://doi.org/10.1039/d6ra02434c.

## References

[cit1] Conn P. J., Christopoulos A., Lindsley C. W. (2009). Allosteric modulators of GPCRs: A novel approach for the treatment of CNS disorders. Nat. Rev. Drug Discovery.

[cit2] Wold E. A., Chen J., Cunningham K. A., Zhou J. (2019). Allosteric modulation of class A GPCRs: Targets, agents, and emerging concepts. J. Med. Chem..

[cit3] Keov P., Sexton P. M., Christopoulos A. (2011). Allosteric modulation of G protein-coupled receptors: a pharmacological perspective. Neuropharmacology.

[cit4] Svensson K. A., Hao J., Bruns R. F. (2019). Positive allosteric modulators of the dopamine D1 receptor: A new mechanism for the treatment of neuropsychiatric disorders. Adv. Pharmacol..

[cit5] Desai A., Benner L., Wu R., Gertsik L., Maruff P., Light G. A., Uz T., Marek G. J., Zhu T. (2021). Phase 1 randomized study on the safety, tolerability, and pharmacodynamic cognitive and electrophysiological effects of a dopamine D_1_ receptor positive allosteric modulator in patients with schizophrenia. Neuropsychopharmacology.

[cit6] Hall A., Provins L., Valade A. (2019). Novel strategies to activate the dopamine D(1) receptor: Recent advances in orthosteric agonism and positive allosteric modulation. J. Med. Chem..

[cit7] Wang X., Heinz B. A., Qian Y. W., Carter J. H., Gadski R. A., Beavers L. S., Little S. P., Yang C. R., Beck J. P., Hao J., Schaus J. M., Svensson K. A., Bruns R. F. (2018). Intracellular binding site for a positive allosteric modulator of the dopamine D1 receptor. Mol. Pharmacol..

[cit8] Luderman K. D., Conroy J. L., Free R. B., Southall N., Ferrer M., Sanchez-Soto M., Moritz A. E., Willette B. K. A., Fyfe T. J., Jain P., Titus S., Hazelwood L. A., Aubé J., Lane J. R., Frankowski K. J., Sibley D. R. (2018). Identification of positive allosteric modulators of the D_1_ dopamine receptor that act at diverse binding sites. Mol. Pharmacol..

[cit9] Wang X., Hembre E. J., Goldsmith P. J., Beck J. P., Svensson K. A., Willard F. S., Bruns R. F. (2023). Mutual cooperativity of three allosteric sites on the dopamine D1 receptor. Mol. Pharmacol..

[cit10] Wang K., Dimova S. T., Hanson J. N., Thakur A., Xu Y., Liu Y., Moritz A. E., Goldberg A., Xie B., Rayadurgam J., Wang F., Nilson A. N., Luderman K. D., Free R. B., Hu W., Zhang J., Xu H. E., Frankowski K. J., Wang Y., Shi L., Sibley D. R., Zhuang Y. (2025). Multiple allosteric sites allow for synergistic enhancement of GPCR signaling. bioRxiv.

[cit11] Lewis M. A., Hunihan L., Watson J., Gentles R. G., Hu S., Huang Y., Bronson J., Macor J. E., Beno B. R., Ferrante M., Hendricson A., Knox R. J., Molski T. F., Kong Y., Cvijic M. E., Rockwell K. L., Weed M. R., Cacace A. A. M., Westphal R. S., Alt A., Brown J. M. (2015). Discovery of D1 dopamine receptor positive allosteric modulators: Characterization of pharmacology and identification of residues that regulate species selectivity. J. Pharmacol. Exp. Ther..

[cit12] Mash E. A., Nelson K. A. (1985). Homochiral ketals in organic synthesis. Diastereoselective cyclopropanation. J. Am. Chem. Soc..

[cit13] Bijvoet J., Peerdeman A., van Bommel A. (1951). Determination of the absolute configuration of optically active compounds by means of X-rays. Nature.

[cit14] Olsen R. H. J., DiBerto J. F., English J. G., Glaudin A. M., Krumm B. E., Slocum S. T., Che T., Gavin A. C., McCorvy J. D., Roth B. L., Strachan R. T. (2020). TRUPATH, an open-source biosensor platform for interrogating the GPCR transducerome. Nat. Chem. Biol..

[cit15] Jiang M., Spicher K., Boulay G., Wang Y., Birnbaumer L. (2001). Most central nervous system D2 dopamine receptors are coupled to their effectors by Go. Proc. Natl. Acad. Sci. U. S. A..

[cit16] Lewis V., Bonniwell E. M., Lanham J. K., Ghaffari A., Sheshbaradaran H., Cao A. B., Calkins M. M., Bautista-Carro M. A., Arsenault E., Telfer A., Taghavi-Abkuh F. F., Malcolm N. J., El Sayegh F., Abizaid A., Schmid Y., Morton K., Halberstadt A. L., Aguilar-Valles A., McCorvy J. D. (2023). A non-hallucinogenic LSD analog with therapeutic potential for mood disorders. Cell Rep..

[cit17] Yuan D.-Y., Tu Y.-Q., Fan C.-A. (2008). Arylation and vinylation of alkenes based on unusual sequential semipinacol rearrangement/Grob fragmentation of allylic alcohols. J. Org. Chem..

[cit18] Blessing R. (1995). An empirical correction for absorption anisotropy. Acta Crystallogr., Sect. A: Found. Crystallogr..

[cit19] Palatinus L., Chapuis G. (2007). Superflip - a computer program for the solution of crystal structures by charge flipping in arbitrary dimensions. J. Appl. Crystallogr..

[cit20] Betteridge P. W., Carruthers J. R., Cooper R. I., Prout K., Watkin D. J. (2003). Software for guided crystal structure analysis. J. Appl. Crystallogr..

[cit21] Sheldrick G. M. (2015). SHELXT – Integrated space-group and crystal-structure determination. Acta Crystallogr., Sect. A:Found. Adv..

[cit22] (a) CCDC 2540023: Experimental Crystal Structure Determination, 2026, 10.5517/ccdc.csd.cc2r837d

