# Supplementary material for: Synthesis and systematic evaluation of four stereoisomers of BMS compound A enabled by a diastereoselective cyclopropanation on a chiral ketal intermediate
Source: RSC Adv. 2026 Jul 12;16(37):39744–56. doi: 10.1039/d6ra02434c (PMC13356873; doi:10.1039/d6ra02434c)
Supplement: Supplementary file 1 [file RA-016-D6RA02434C-s001.pdf]

Supplementary Information (SI) for RSC Advances.
This journal is © The Royal Society of Chemistry 2026

Synthesis and Systematic Evaluation of Four Stereoisomers of BMS Compound A Enabled by a Diastereoselective Cyclopropanation on a Chiral Ketal Intermediate

(Supplementary Information)

Feijun Wang[1], John N. Hanson[2+], Snezana T. Dimova[2+], Jayachandra Rayadurgam[1], Camryn J. Fulton[3], Amy E. Moritz[2], Ashley N. Nilson[2], William A. Hearne[4], Chun-Hsing Chen[4], Ryan H. Gumpper[1], David R. Sibley[2]*, Kevin J. Frankowski[1]*

[1]Chemical Biology and Medicinal Chemistry Division, UNC Eshelman School of Pharmacy, University of North Carolina at Chapel Hill, Chapel Hill, North Carolina, United States

[2]Molecular Neuropharmacology Section, National Institute of Neurological Disorders and Stroke, National Institutes of Health, Bethesda, Maryland, United States

[3]Department of Pharmacology, UNC School of Medicine, University of North Carolina at Chapel Hill, Chapel Hill, North Carolina, United States

[4]Department of Chemistry X-ray Crystallography Core, University of North Carolina at Chapel Hill, Chapel Hill, North Carolina, United States

*Correspondence to: K.J.F. (kevinf@unc.edu), D.R.S (sibleyd@ninds.nih.gov)

+These authors contributed equally

Contents:

- NMR spectra for synthesized compounds S-2 to S-15
- Chiral SFC analysis for intermediate **9** S-8 to S-9
- Chiral SFC analysis for intermediate **10** S-10 to S-11
- Chiral SFC analysis for synthesized BMS Compound A isomer 2 S-13 to S-14
- Chiral SFC analysis for synthesized BMS Compound A isomer 4 S-16 to S-17
- Chiral HPLC Separation Report (analytical HPLC analysis of isolated isomers) S-18 to S-28
- **Figure S1.** Concentration–response curves of BMS Compound A isomers for potentiating dopamine-stimulated β-arrestin recruitment to the D1R S-28 to S-29
- **Table S1.** Crystal data and structure refinement for BMS Compound A (isomer 1) S-30
- **Table S2.** Crystal data and structure refinement for BMS Compound A (isomer 4) S-31

8-(Benzo[*d*][1,3]dioxol-5-yl)-1,4-dioxaspiro[4.5]dec-7-ene **3**. ^{1}H (400 MHz, $CDCl_3$)

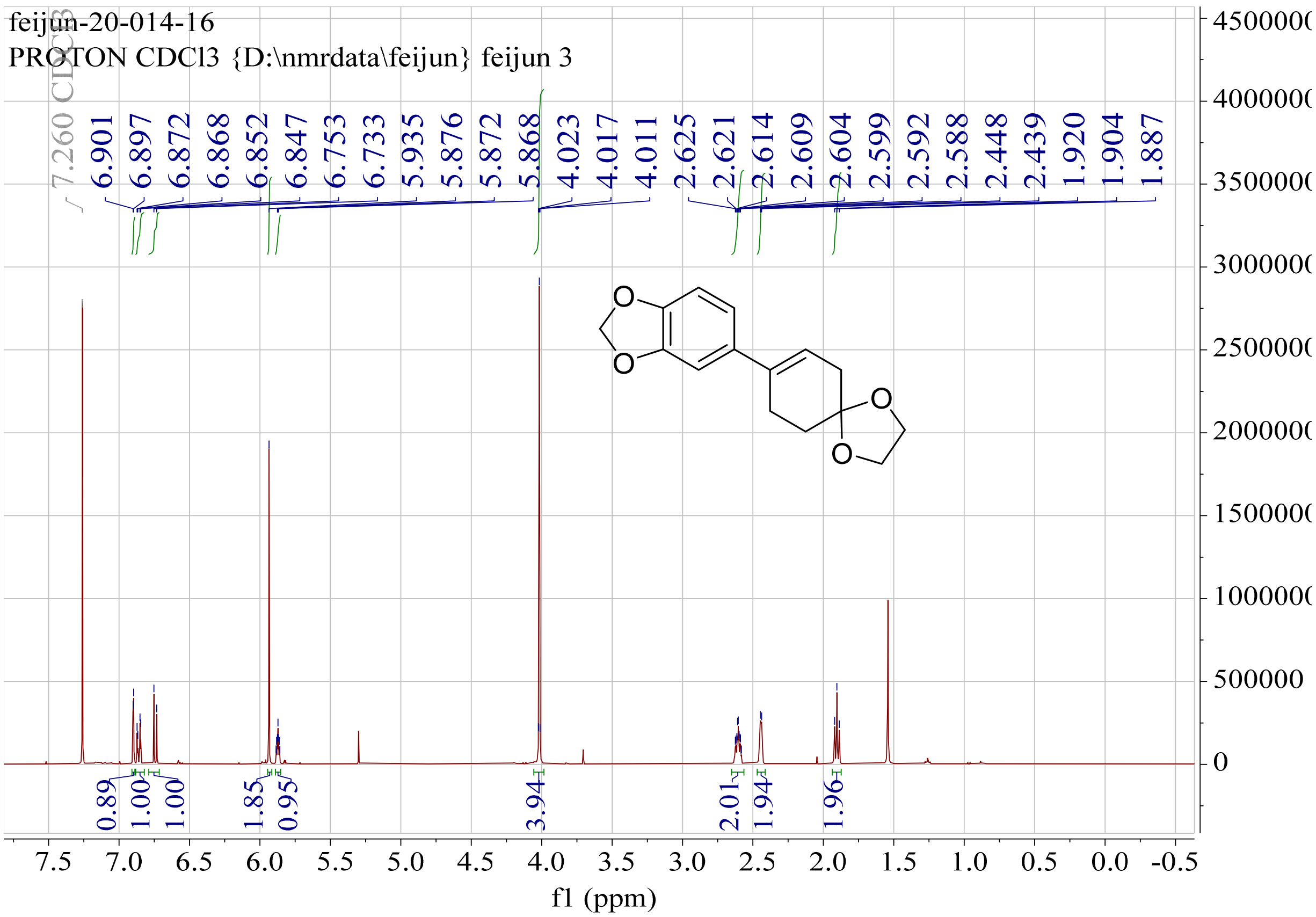

6-(Benzo[*d*][1,3]dioxol-5-yl)bicyclo[4.1.0]heptan-3-one **5**. ^{1}H (400 MHz, $CDCl_3$), ^{13}C (126 MHz, $CDCl_3$)

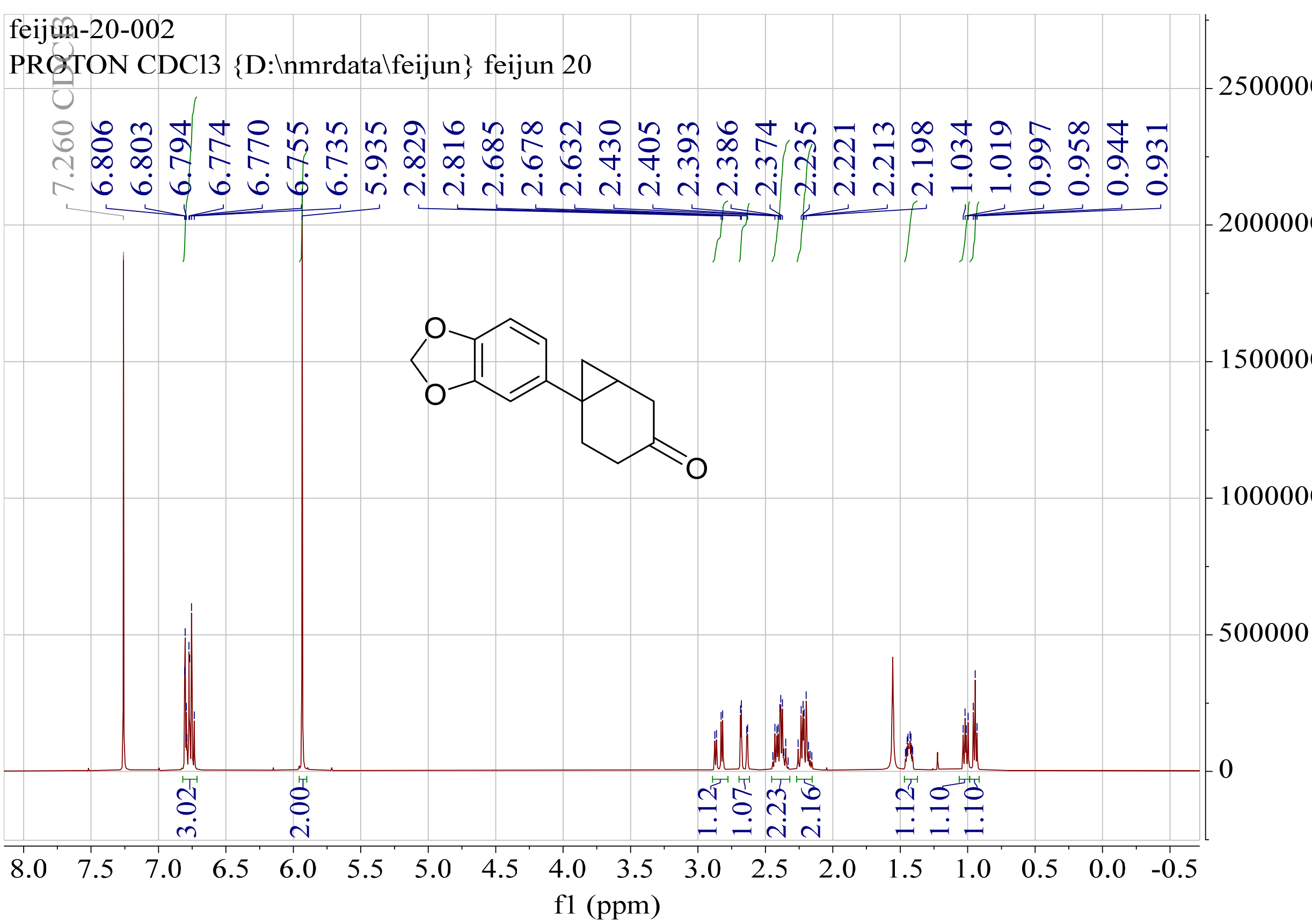

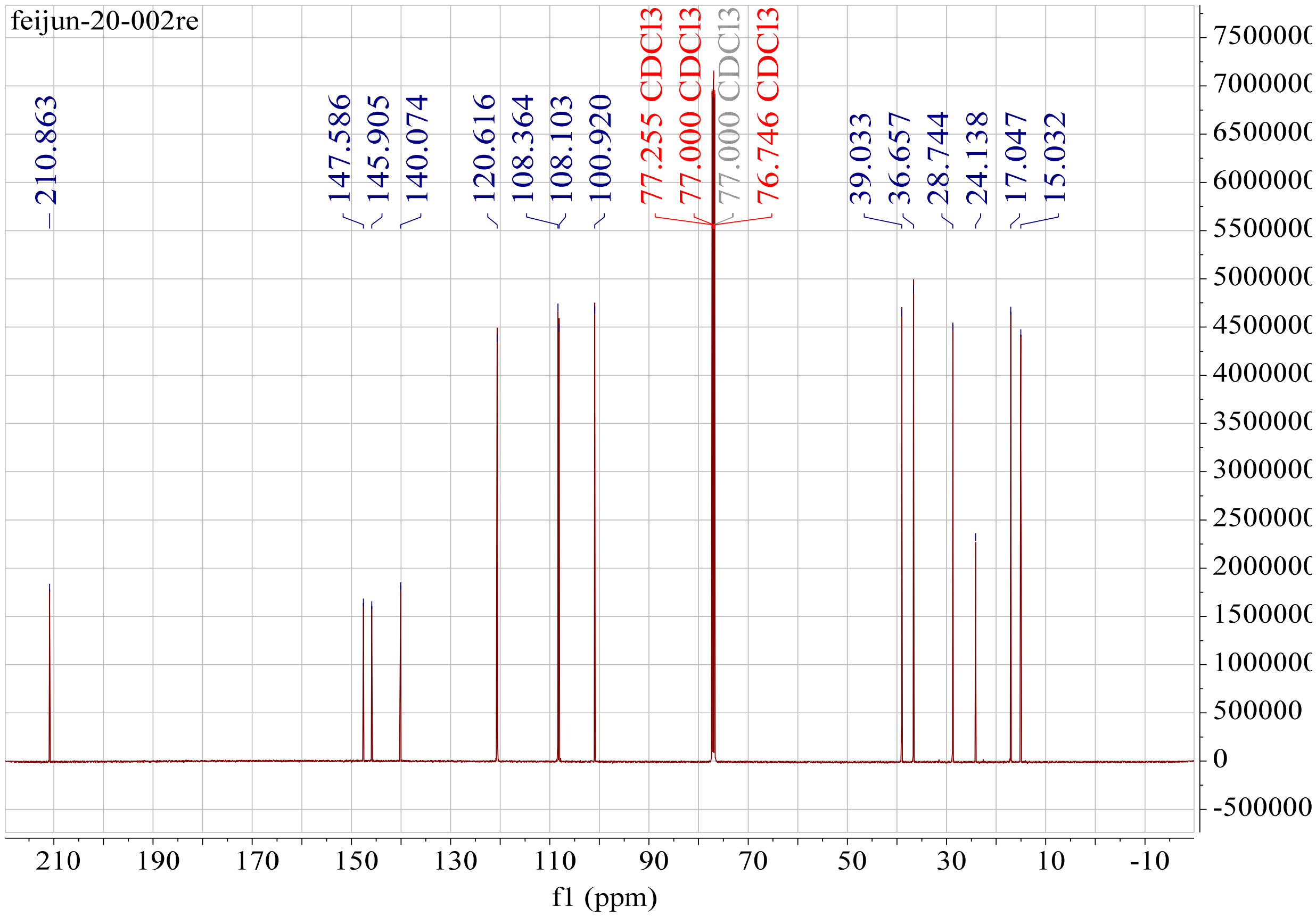

tert-Butyl 4-(2-bromo-5-chlorobenzyl)piperazine-1-carboxylate **7**. ^{1}H (400 MHz, $CDCl_3$), ^{13}C (126 MHz, $CDCl_3$)

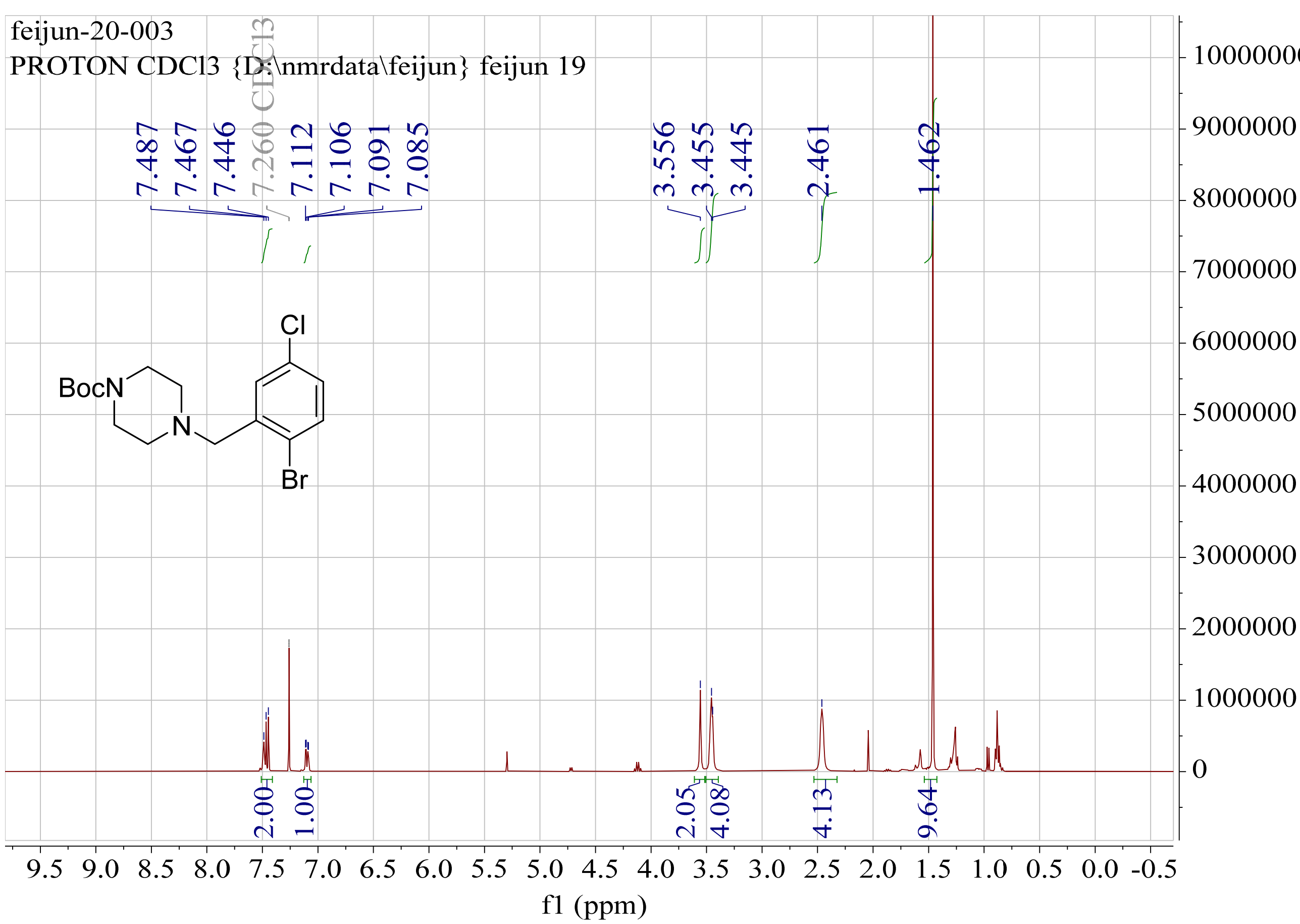

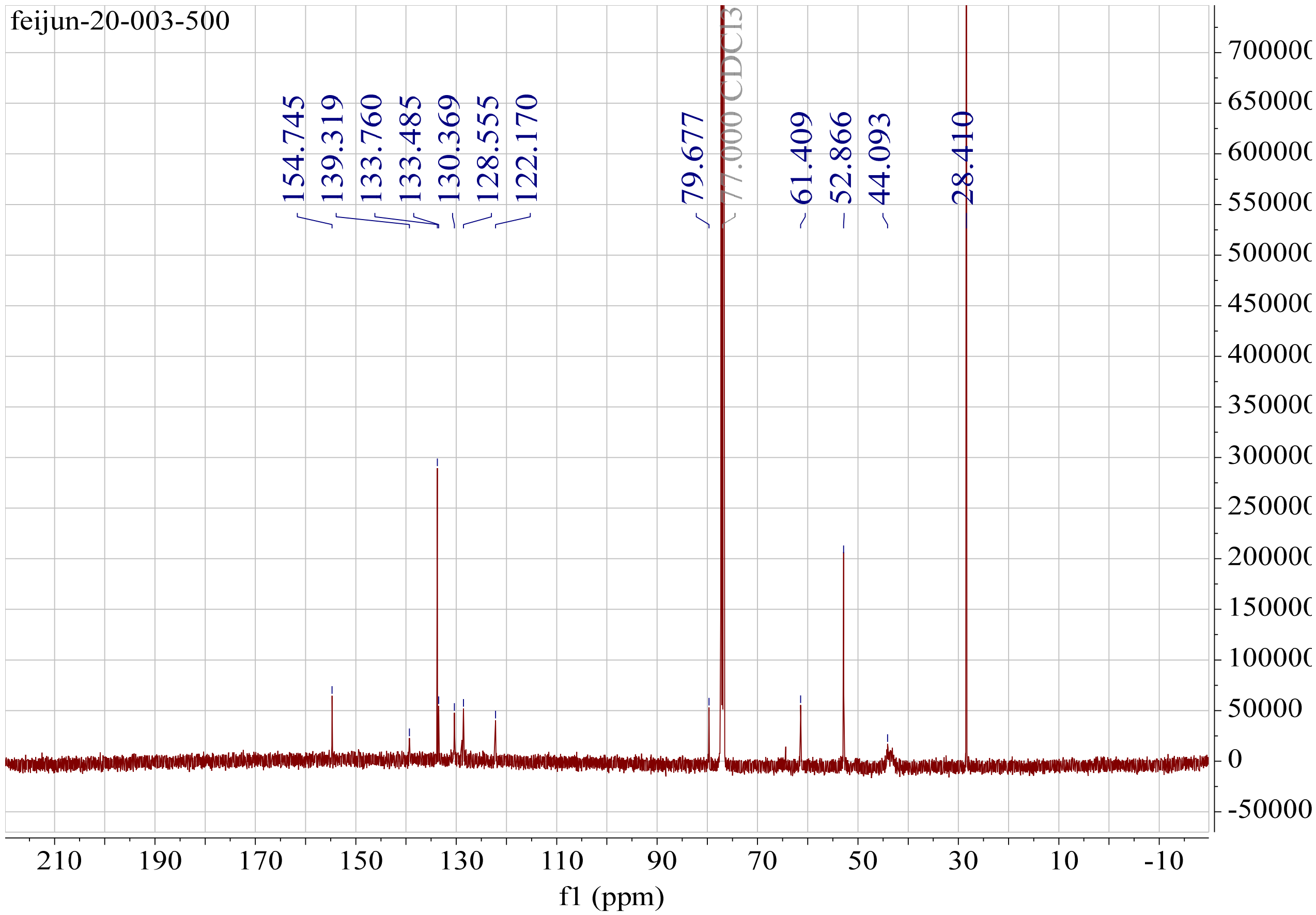

(±)-*syn*-**BMS Compound A.** ^{1}H (400 MHz, $CDCl_3$)

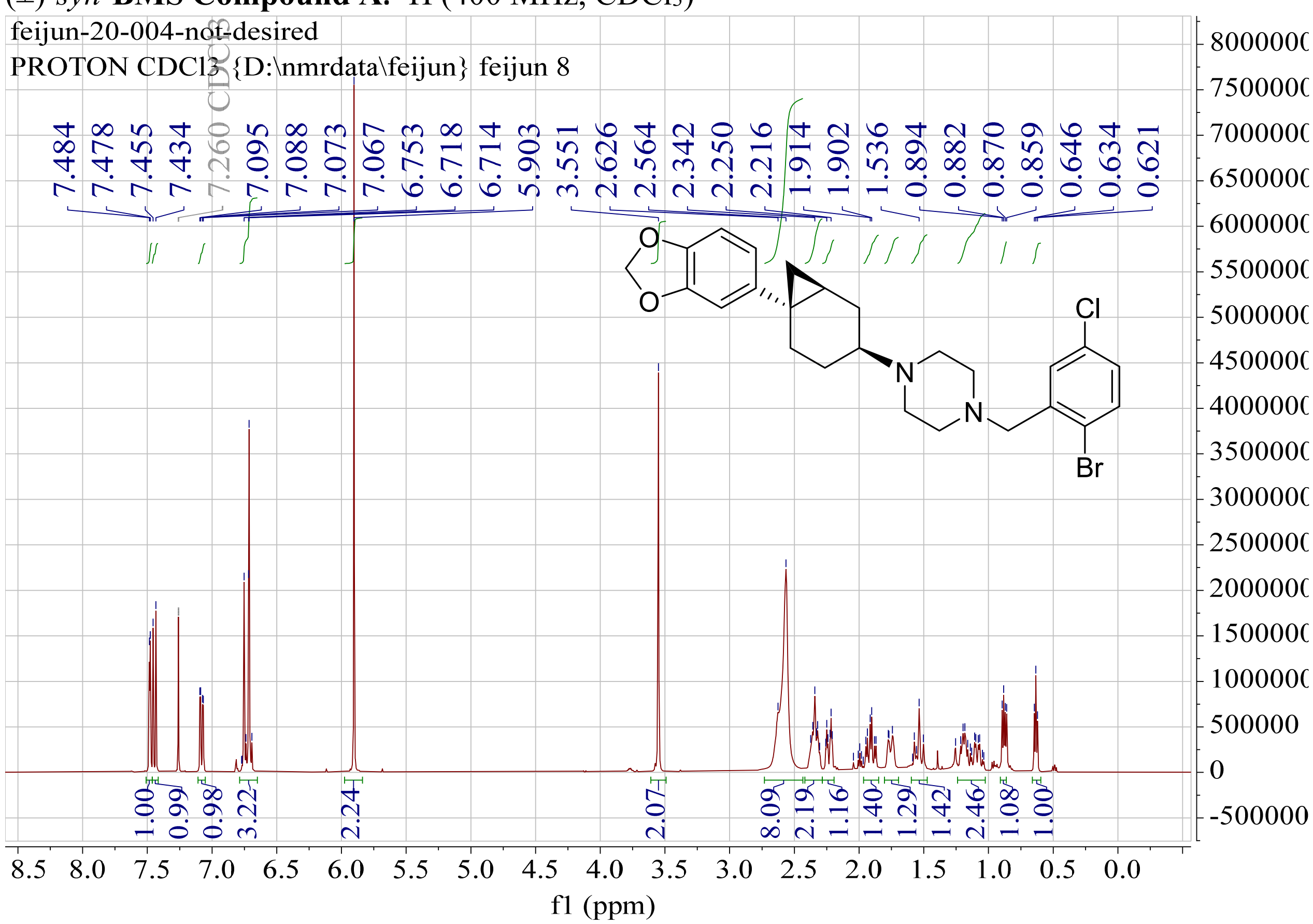

(±)-*anti*-**BMS Compound A.** ^{1}H (400 MHz, $CDCl_3$)

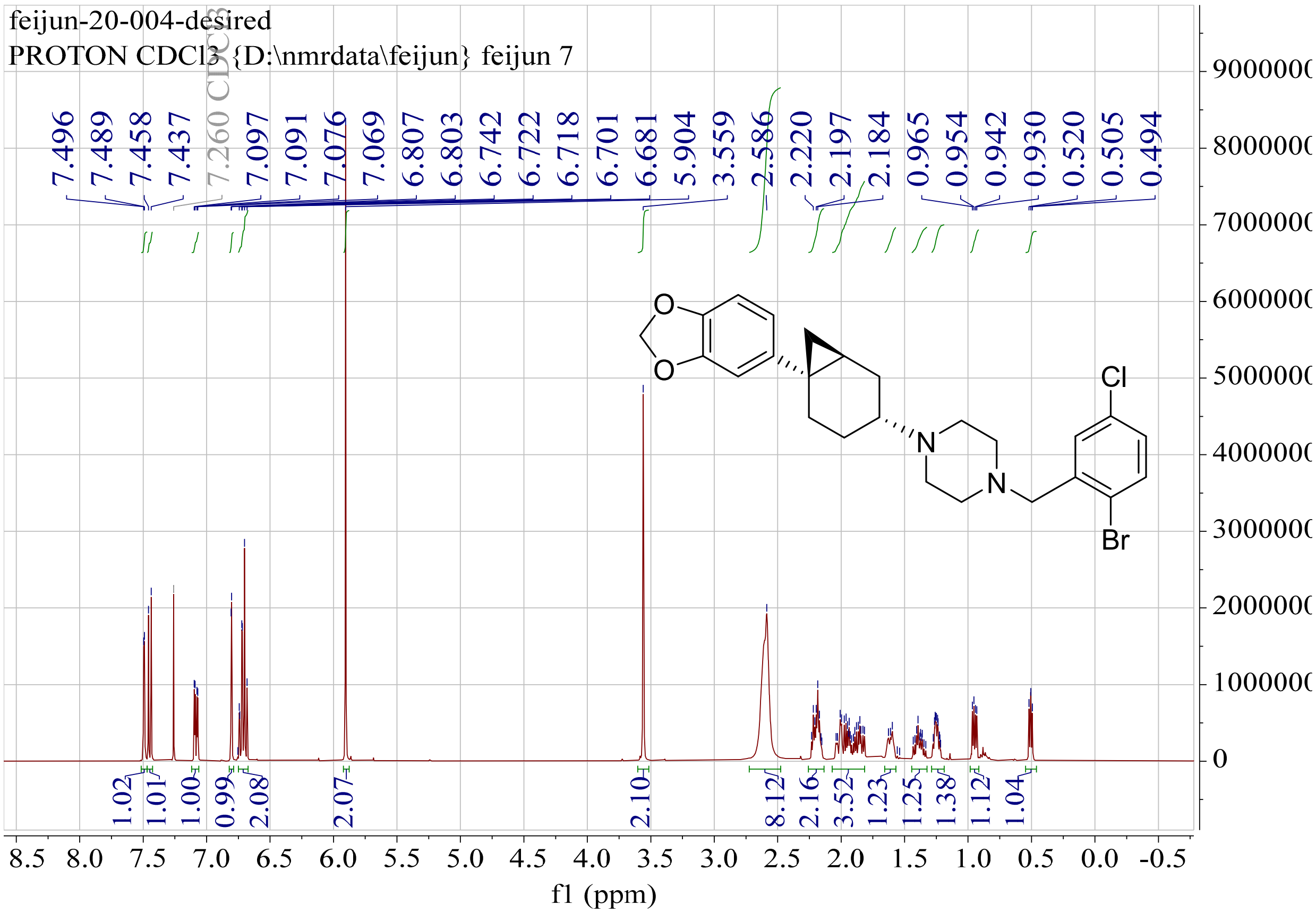

4-(Benzo[*d*][1,3]dioxol-5-yl)cyclohex-3-en-1-one **8**. ^{1}H (400 MHz, $CDCl_3$), ^{13}C (126 MHz, $CDCl_3$)

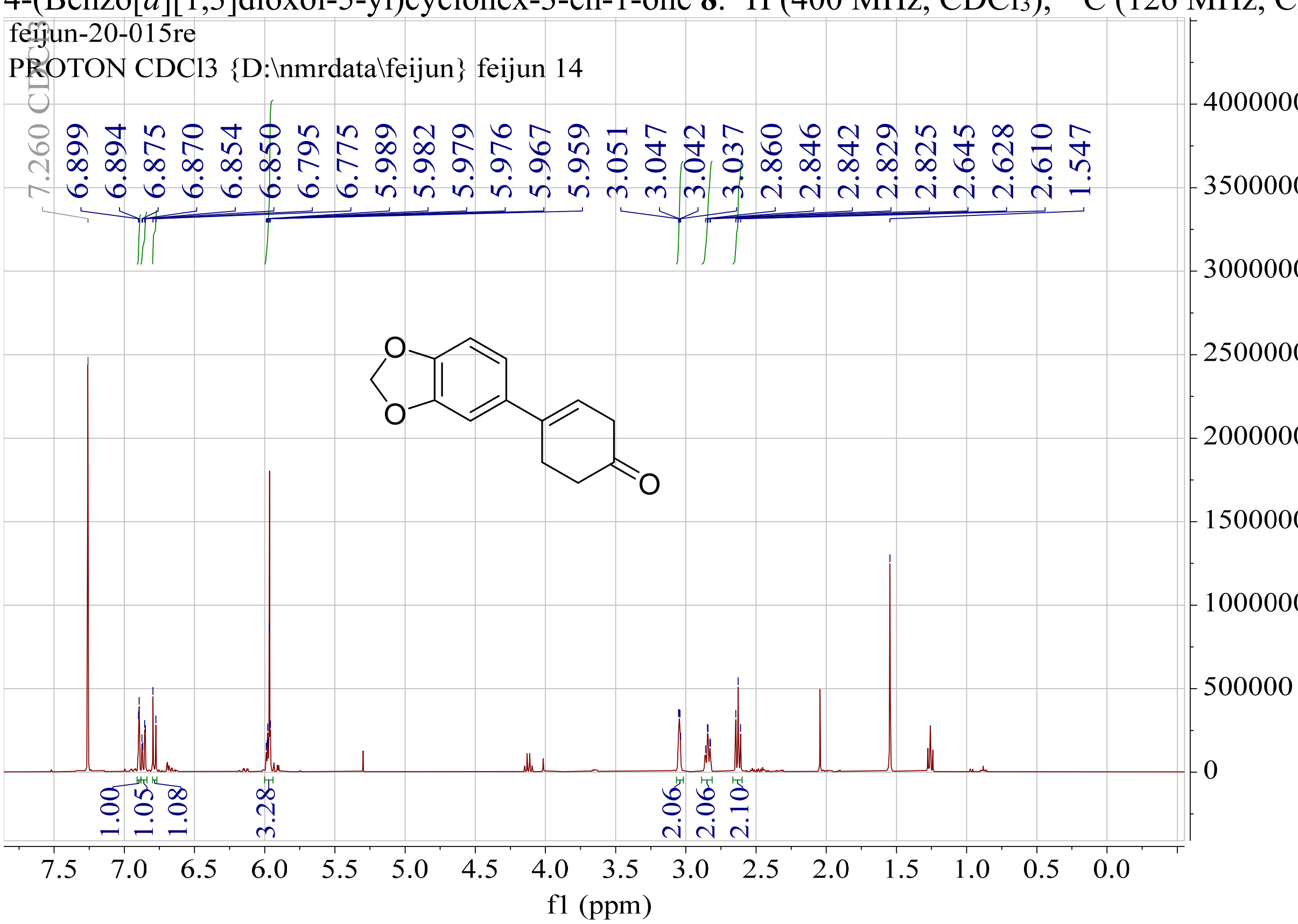

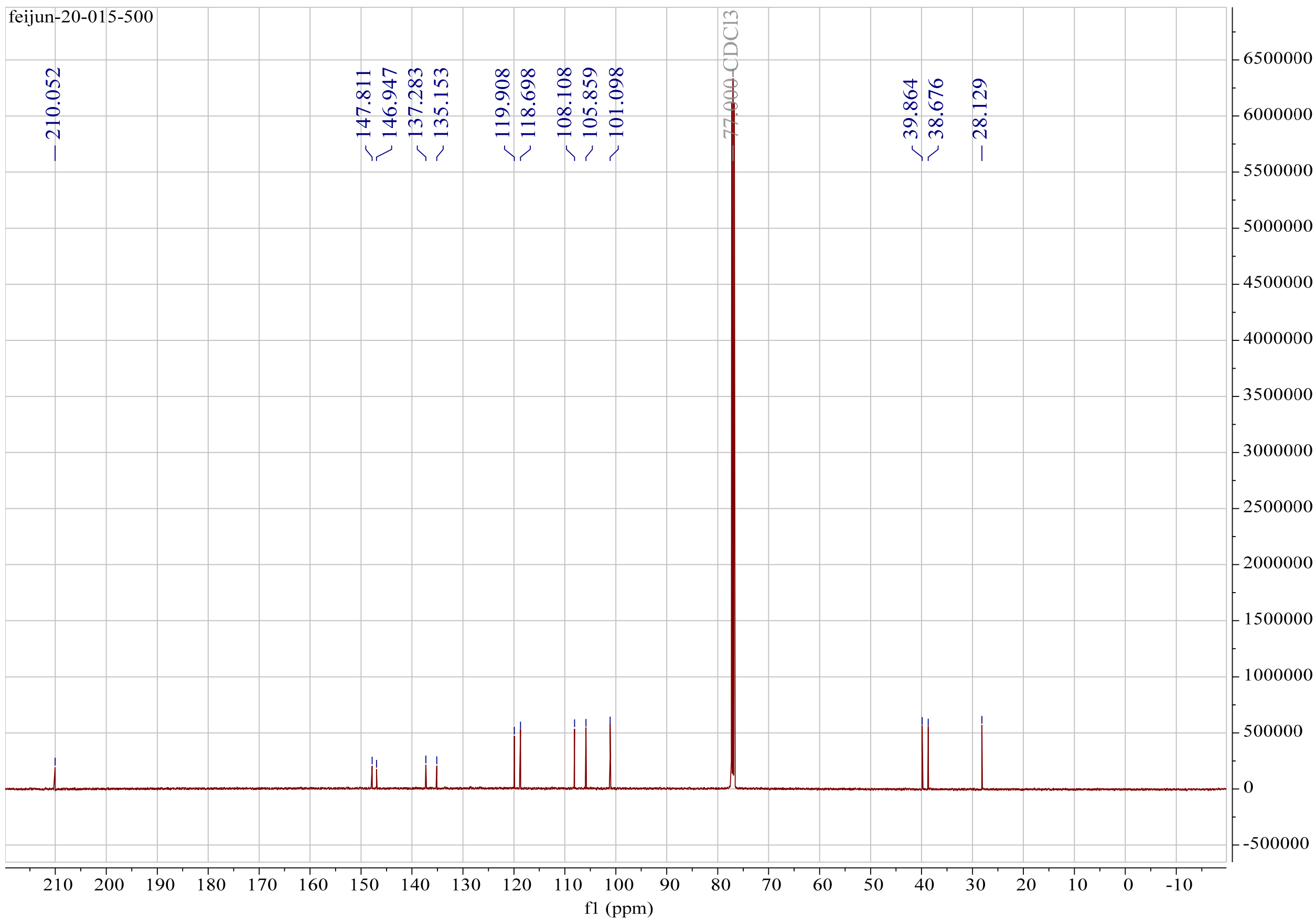

(2*R*,3*R*)-8-(Benzo[*d*][1,3]dioxol-5-yl)-2,3-diphenyl-1,4-dioxaspiro[4.5]dec-7-ene **9**. ^{1}H (400 MHz, $CDCl_3$), ^{13}C (126 MHz, $CDCl_3$)

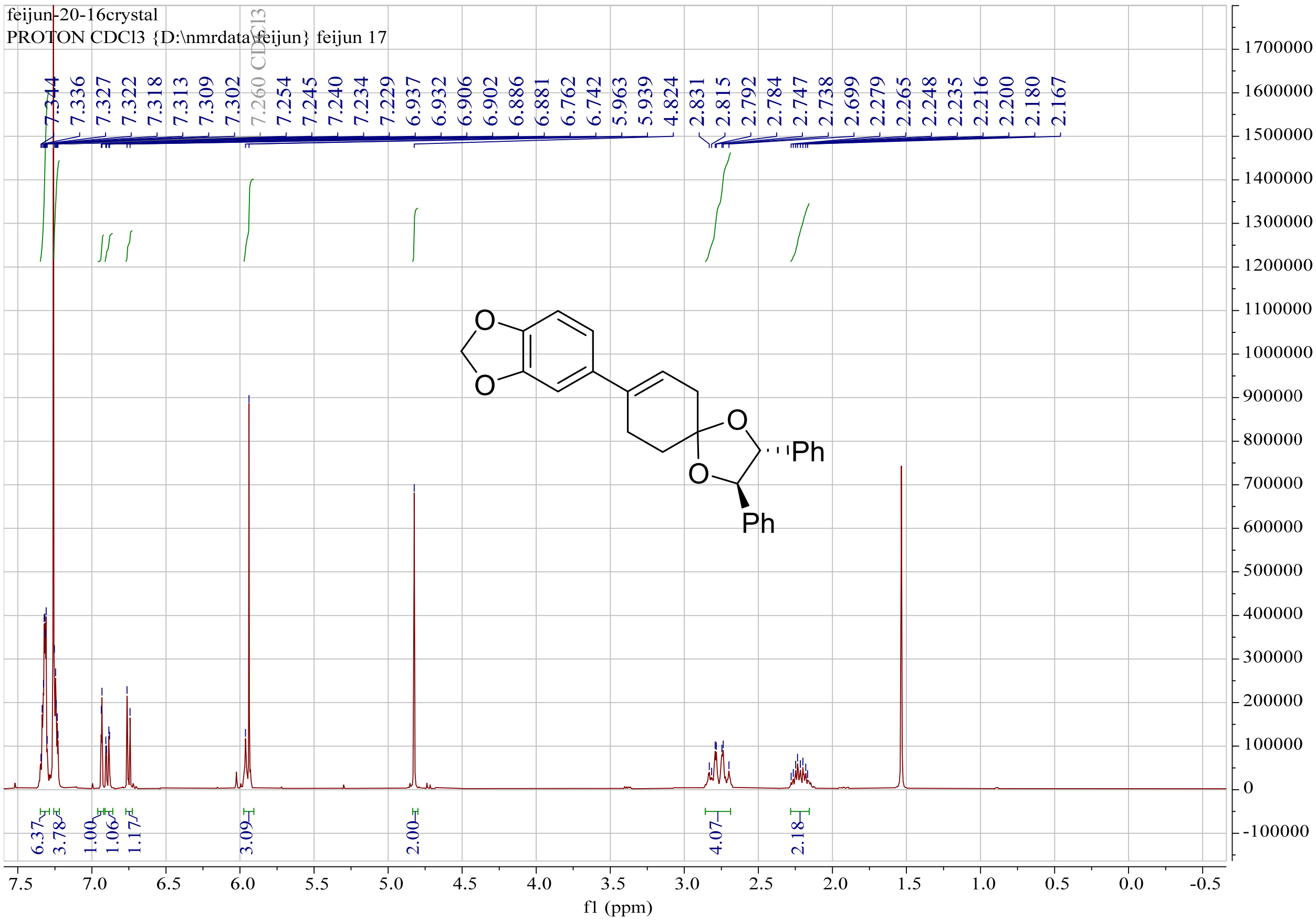

feijun-20-16crystal
PROTON CDCl3 {D:\nmrdata\feijun} feijun 17
7.344 7.336 7.327 7.322 7.318 7.313 7.309 7.302 7.260 CDCl3 7.254 7.245 7.240 7.234 7.229 6.937 6.932 6.906 6.902 6.886 6.881 6.762 6.742 5.963 5.939 4.824 2.831 2.815 2.792 2.784 2.747 2.738 2.699 2.279 2.265 2.248 2.235 2.216 2.200 2.180 2.167
O
O
O
O
Ph
Ph
6.37 3.78 1.00 1.06 1.17 3.09 2.00 4.07 2.18
f1 (ppm)

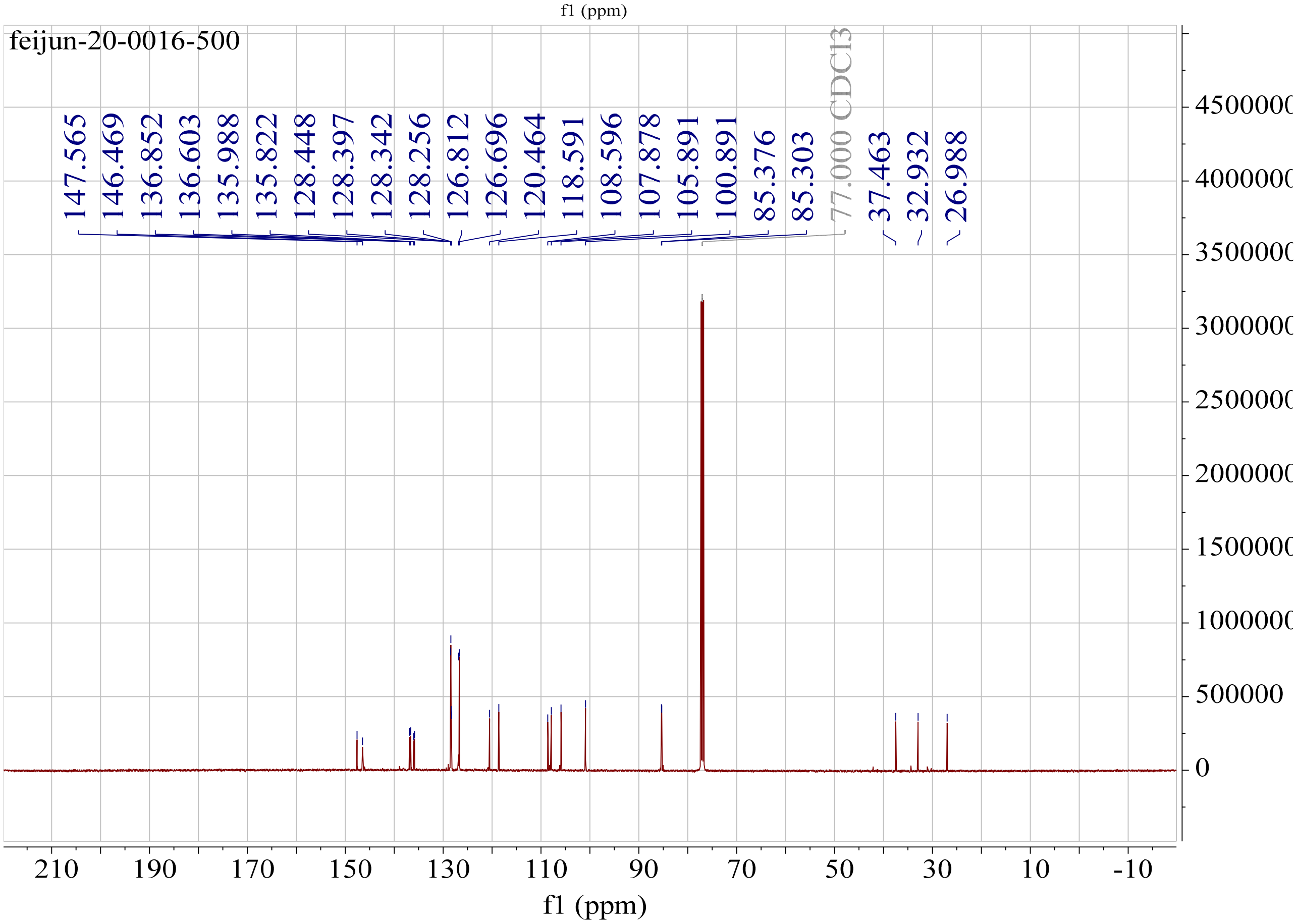

feijun-20-0016-500
147.565 146.469 136.852 136.603 135.988 135.822 128.448 128.397 128.342 128.256 126.812 126.696 120.464 118.591 108.596 107.878 105.891 100.891 85.376 85.303 77.000 CDCl3 37.463 32.932 26.988
f1 (ppm)

SFC separation of (±)-(*rel*-2*R*,3*R*)-8-(Benzo[*d*][1,3]dioxol-5-yl)-2,3-diphenyl-1,4-dioxaspiro[4.5]dec-7-ene **9**. Conditions: Chiralpak IC column, flow rate = 3.0 mL/min; 35 ºC; CO_2/*i*-PrOH = 95/5. Integration of chromatogram shows 1:1 ratio of separated enantiomers (rt = 9.48 and 13.16 minutes).

**Sample name:**	wfj-20-016-rac4		
**Data file:**	wfj-20-016-rac4SFC IC Iso 5% IPA 15min.amx20260429 094902.dx	**Operator:**	SYSTEM (SYSTEM)
**Instrument:**	SFC - HPLC	**Injection date:**	2026-04-29 10:08:05-04:00
**Inj. volume:**	2.000 µL	**Location:**	5
**Acq. method:**	SFC IC Iso 5% IPA 15min.amx	**Type:**	Sample
**Processing method:**	GC_LC Area Percent_DefaultMethod.pmx	**Calib level:**	
		**Sample amount:**	0.00
**Manually modified:**	Manual Integration	**ISTD amounts:**	None

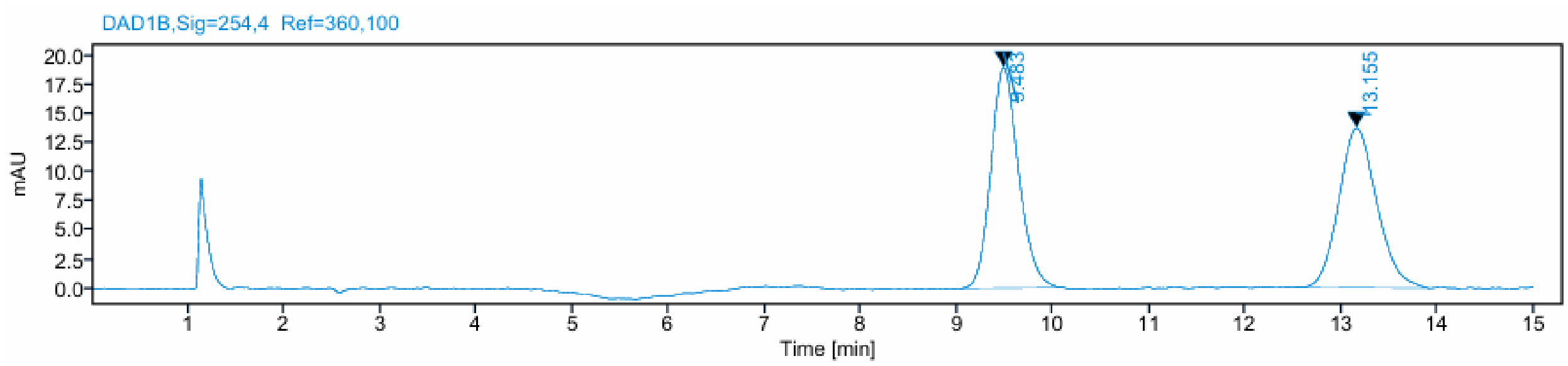

Signal: DAD1B,Sig=254,4 Ref=360,100

RT [min]	Type	Width [min]	Area	Height	Area%	Name
9.483	MM m	1.0621	380.8449	18.9282	50.3112	
13.155	BB	1.3933	376.1330	13.7033	49.6888	
		**Sum**	**756.9779**			

SFC analysis of (+)-(2*R*,3*R*)-8-(Benzo[*d*][1,3]dioxol-5-yl)-2,3-diphenyl-1,4-dioxaspiro[4.5]dec-7-ene **9**. Conditions: Chiralpak IC column, flow rate = 3.0 mL/min; 35 ºC; CO_2/*i*-PrOH = 95/5. Integration of chromatogram shows almost exclusively a single enantiomer (ratio = 99.5:0.5) of separated enantiomers (rt = 9.51 for major peak).

**Sample name:**	wfj-20-016-ee		
**Data file:**	wfj-20-016-eeSFC IC Iso 5% IPA 15min.amx20260429 101022.dx	**Operator:**	SYSTEM (SYSTEM)
**Instrument:**	SFC - HPLC	**Injection date:**	2026-04-29 10:45:41-04:00
**Inj. volume:**	2.000 µL	**Location:**	4
**Acq. method:**	SFC IC Iso 5% IPA 15min.amx	**Type:**	Sample
**Processing method:**	GC_LC Area Percent_DefaultMethod.pmx	**Calib level:**	
		**Sample amount:**	0.00
**Manually modified:**	Manual Integration	**ISTD amounts:**	None

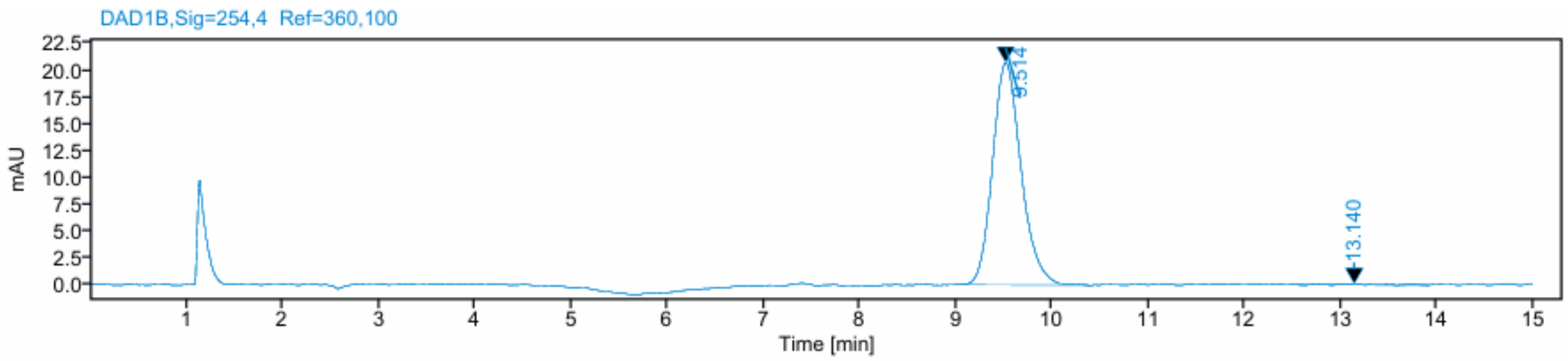

Signal: DAD1B,Sig=254,4 Ref=360,100

RT [min]	Type	Width [min]	Area	Height	Area%	Name
9.514	BB	1.3267	430.2956	20.8036	99.5800	
13.140	MM m	1.3762	1.8150	0.0833	0.4200	
		**Sum**	**432.1106**			

(1*S*,4'*R*,5'*R*,6*R*)-6-(Benzo[*d*][1,3]dioxol-5-yl)-4',5'-diphenylspiro[bicyclo[4.1.0]heptane-3,2'-[1,3]dioxolane] **10**.
^{1}H (400 MHz, $CDCl_3$), ^{13}C (126 MHz, $CDCl_3$)

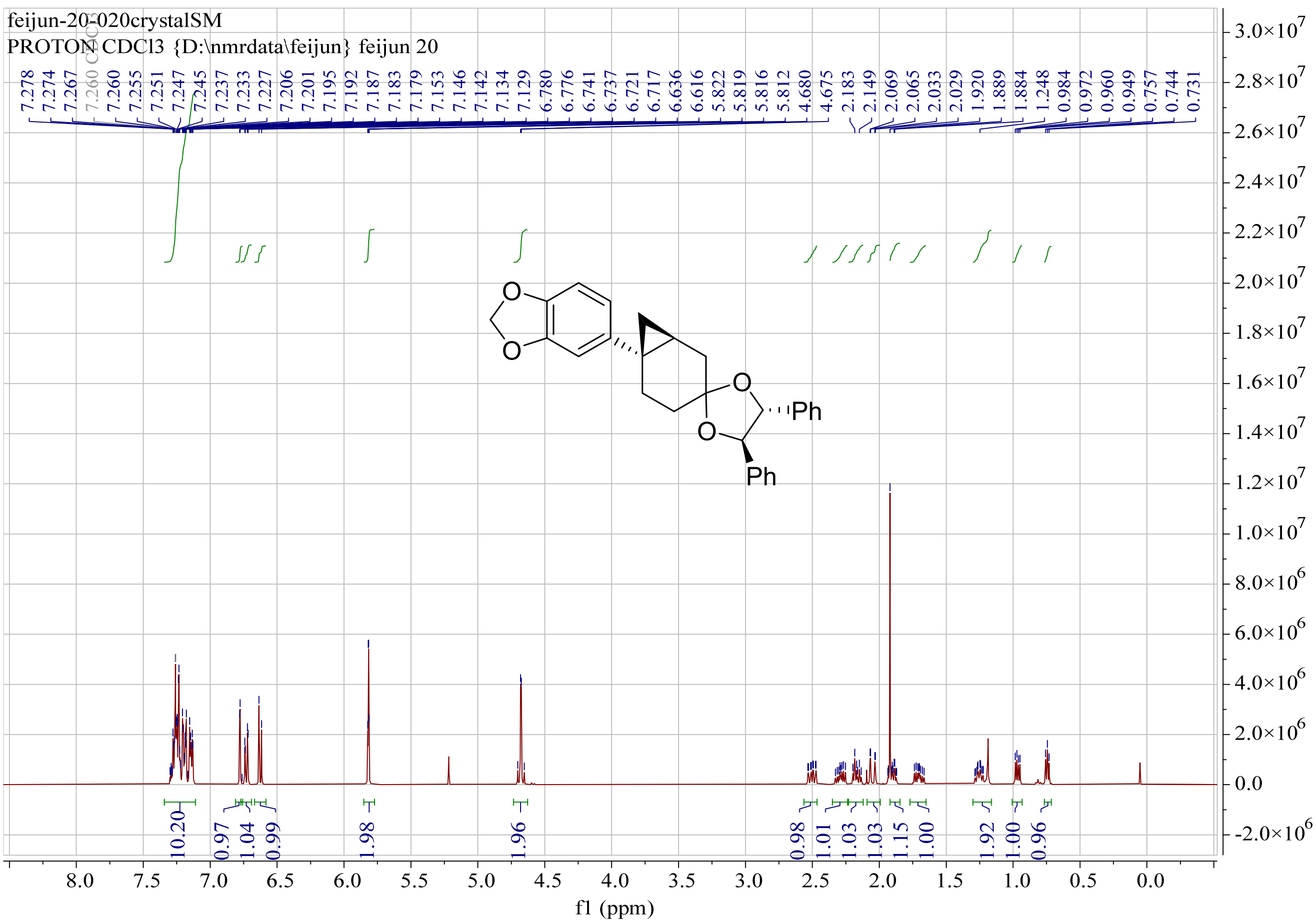

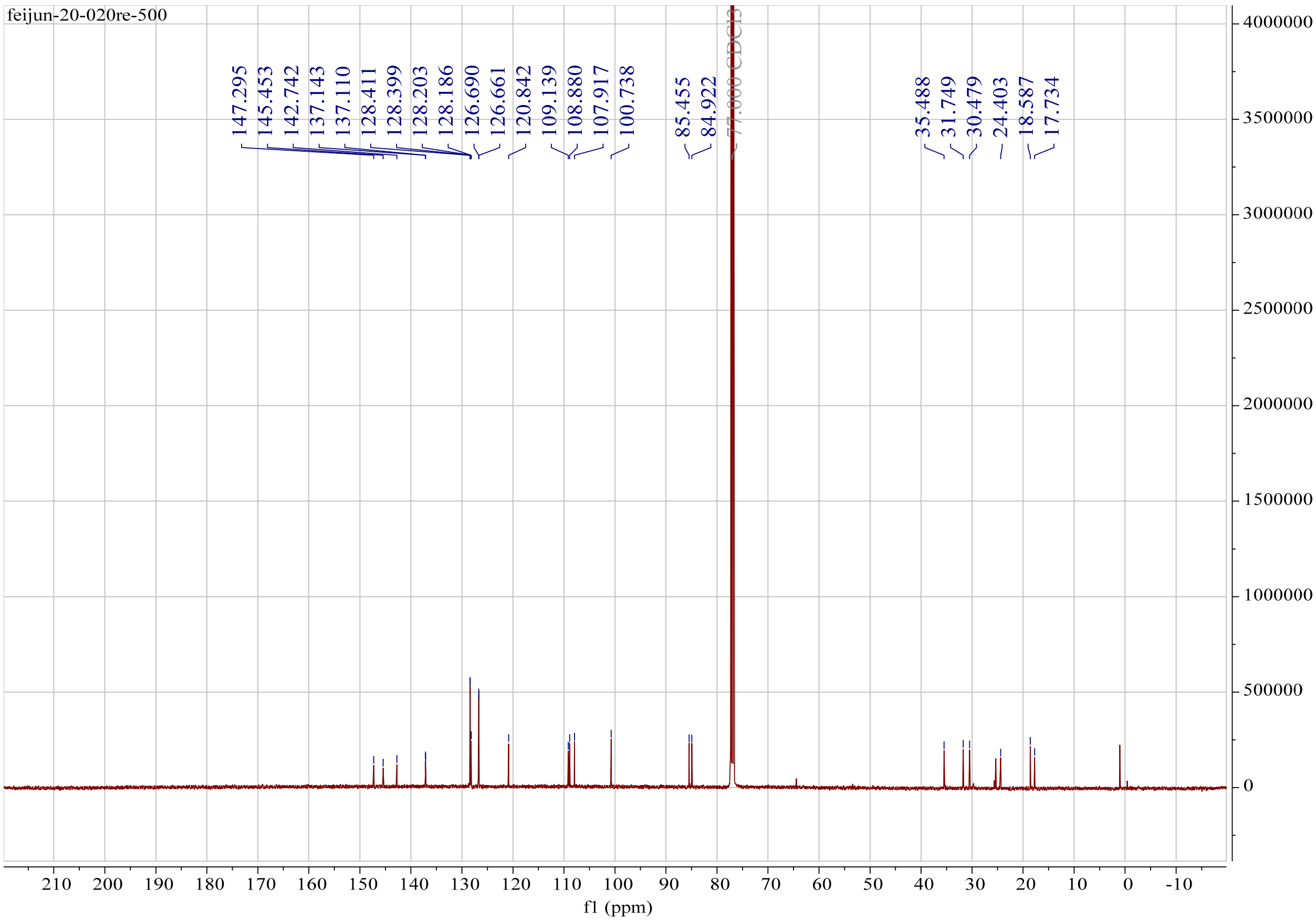

SFC separation of (1*S*,4'*R*,5'*R*,6*R*)-6-(Benzo[*d*][1,3]dioxol-5-yl)-4',5'-diphenylspiro[bicyclo[4.1.0]heptane-3,2'-[1,3]dioxolane] (+)-**10** and (1*R*,4'*S*,5'*S*,6*S*)-6-(benzo[*d*][1,3]dioxol-5-yl)-4',5'-diphenylspiro[bicyclo[4.1.0]heptane-3,2'-[1,3]dioxolane] (-)-**10**. Conditions: Chiralpak IC column, flow rate = 3.0 mL/min; 35 ºC; CO_2/*i*-PrOH = 95/5. Integration of chromatogram shows 1:1 ratio of separated enantiomers (rt = 5.68 and 7.28 minutes).

**Sample name:**	wfj-20-020-rac1		
**Data file:**	wfj-20-020-rac1SFC IC Iso 5% IPA 10min.amx20260505 120427.dx	**Operator:**	SYSTEM (SYSTEM)
**Instrument:**	SFC - HPLC	**Injection date:**	2026-05-05 12:18:43-04:00
**Inj. volume:**	2.000 µL	**Location:**	5
**Acq. method:**	SFC IC Iso 5% IPA 10min.amx	**Type:**	Sample
**Processing method:**	GC_LC Area Percent_DefaultMethod.pmx	**Calib level:**	
		**Sample amount:**	0.00
**Manually modified:**	Manual Integration	**ISTD amounts:**	None

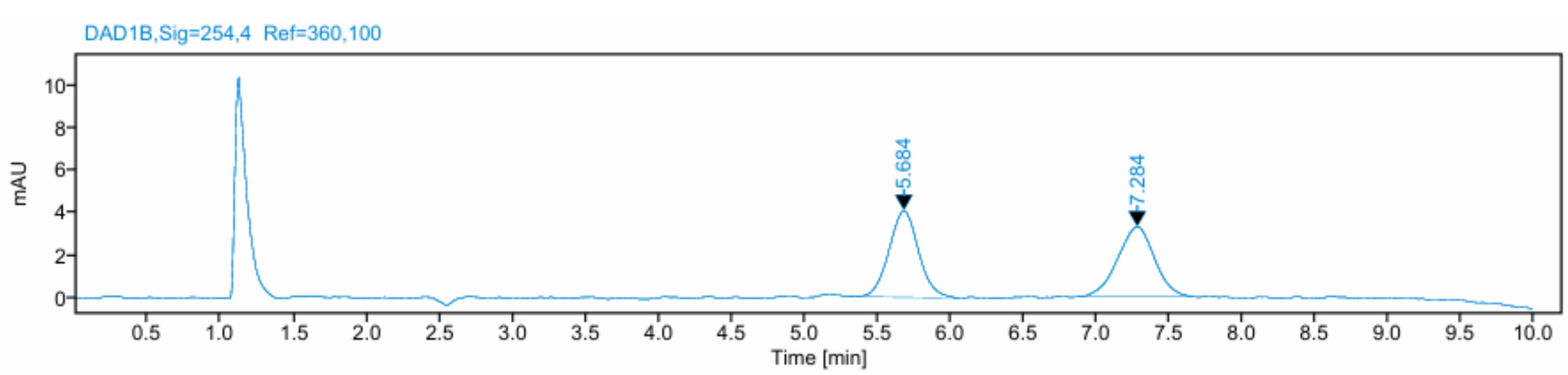

Signal: DAD1B,Sig=254,4 Ref=360,100

RT [min]	Type	Width [min]	Area	Height	Area%	Name
5.684	BB	0.7456	57.6946	4.0981	49.7373	
7.284	MM m	0.7813	58.3040	3.3015	50.2627	
		Sum	115.9986			

SFC analysis of (1*S*,4'*R*,5'*R*,6*R*)-6-(Benzo[*d*][1,3]dioxol-5-yl)-4',5'-diphenylspiro[bicyclo[4.1.0]heptane-3,2'-[1,3]dioxolane] (+)-**10**. Conditions: Chiralpak IC column, flow rate = 3.0 mL/min; 35 ºC; CO_2/*i*-PrOH = 95/5. Integration of chromatogram shows almost exclusively a single enantiomer (ratio = 99.5:0.5) of separated enantiomers (rt = 5.72 for major peak).

**Sample name:**	wfj-20-020-eere		
**Data file:**	wfj-20-020-eereSFC IC Iso 5% IPA 10min.amx20260505 121658.dx	**Operator:**	SYSTEM (SYSTEM)
**Instrument:**	SFC - HPLC	**Injection date:**	2026-05-05 12:46:18-04:00
**Inj. volume:**	2.000 µL	**Location:**	1
**Acq. method:**	SFC IC Iso 5% IPA 10min.amx	**Type:**	Sample
**Processing method:**	GC_LC Area Percent_DefaultMethod.pmx	**Calib level:**	
		**Sample amount:**	0.00
**Manually modified:**	Manual Integration	**ISTD amounts:**	None

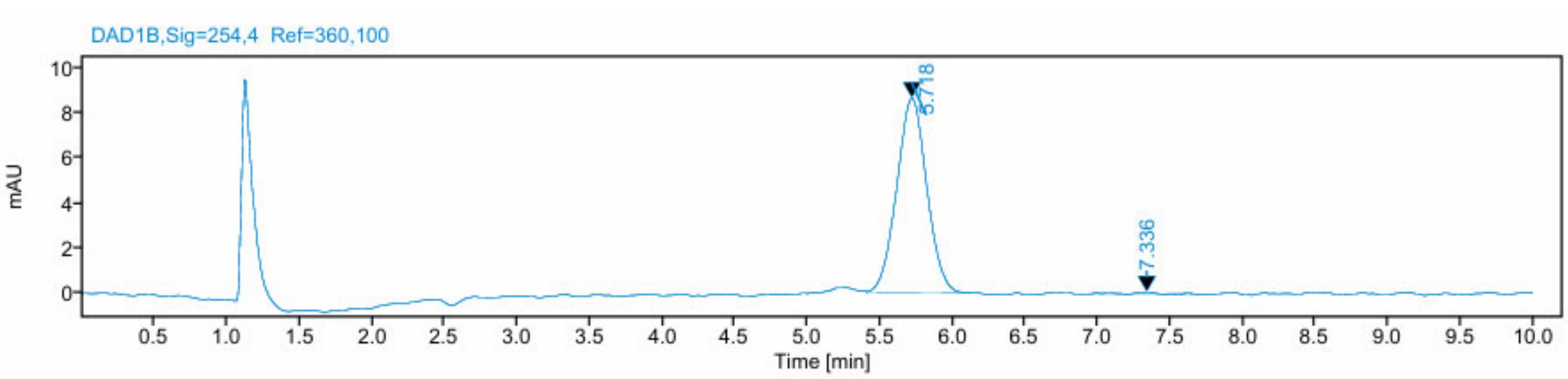

Signal: DAD1B,Sig=254,4 Ref=360,100

RT [min]	Type	Width [min]	Area	Height	Area%	Name
5.718	VM m	0.7135	125.0317	8.6533	99.5547	
7.336	MM m	0.7016	0.5593	0.0800	0.4453	
		Sum	125.5909			

1-(6-((1*S*,3*R*,6*R*)-Benzo[*d*][1,3]dioxol-5-yl)bicyclo[4.1.0]heptan-3-yl)-4-(2-bromo-5-chlorobenzyl)piperazine **BMS Compound A** (isomer 2). ^{1}H (400 MHz, $CDCl_3$), ^{13}C (126 MHz, $CDCl_3$).

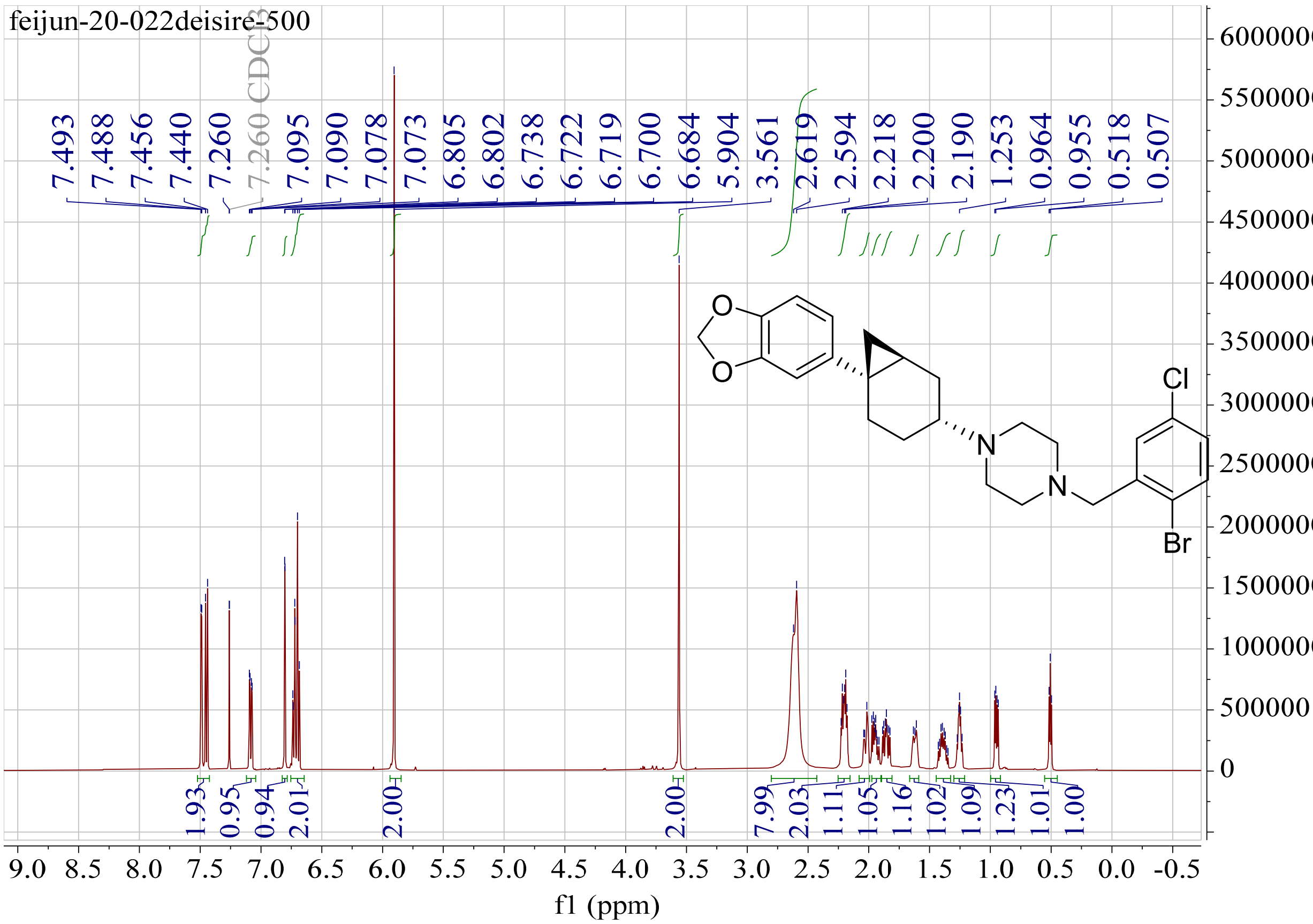
feijun-20-022deisire-500
7.493
7.488
7.456
7.440
7.260
7.260 CDCl3
7.095
7.090
7.078
7.073
6.805
6.802
6.738
6.722
6.719
6.700
6.684
5.904
3.561
2.619
2.594
2.218
2.200
2.190
1.253
0.964
0.955
0.518
0.507
1.93
0.95
0.94
2.01
2.00
2.00
7.99
2.03
1.11
1.05
1.16
1.02
1.09
1.23
1.01
1.00
O
O
N
N
Cl
Br
f1 (ppm)

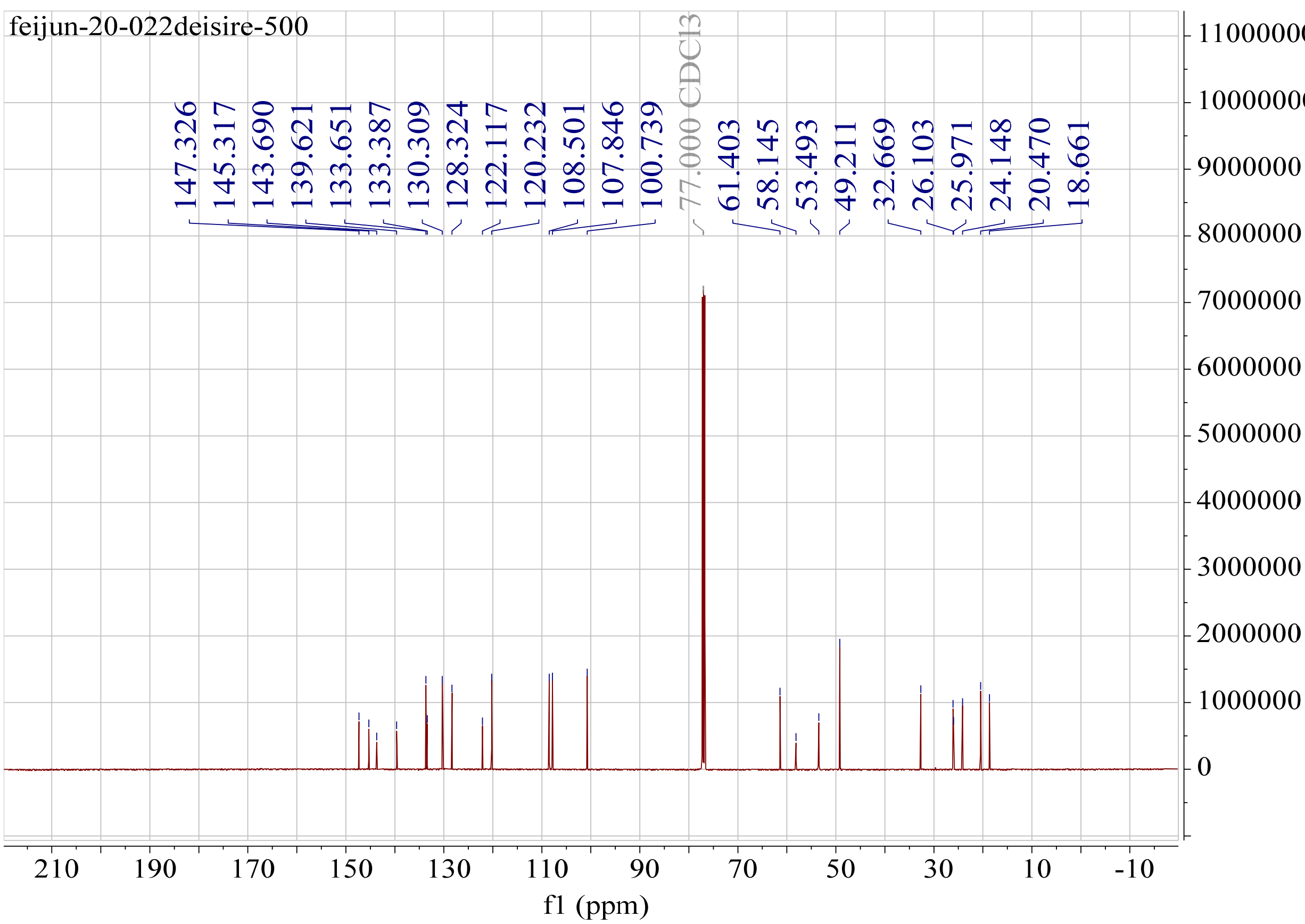
feijun-20-022deisire-500
147.326
145.317
143.690
139.621
133.651
133.387
130.309
128.324
122.117
120.232
108.501
107.846
100.739
77.000 CDCl3
61.403
58.145
53.493
49.211
32.669
26.103
25.971
24.148
20.470
18.661
f1 (ppm)

SFC separation of BMS Compound A (isomers 1, 2, 3 and 4): Conditions: Chiralpak IC column, flow rate = 2.0 mL/min; 35 ºC; CO_2/*i*-PrOH with 0.1% dimethylethylamine (DMEA): 80/20. Integration of chromatogram shows 4.9:4.9:33.4:56.8 ratio of separated stereoisomers (rt = 9.09, 10.05,12.34 and 12.80 minutes). These conditions provide clean separation of isomers 1 and 2 from each other and from their diastereomers (isomers 3 and 4), however nonideal separation of isomers 3 and 4 from each other (see below for complementary conditions to cleanly separate isomers 3 and 4 from each other).

**Sample name:**	wfj-20-004		
**Data file:**	wfj-20-004SFC IC Iso 20% IPA DMEA 20min.amx20260420 154023.dx	**Operator:**	SYSTEM (SYSTEM)
**Instrument:**	SFC - HPLC	**Injection date:**	2026-04-20 16:05:05-04:00
**Inj. volume:**	2.000 µL	**Location:**	1
**Acq. method:**	SFC IC Iso 20% IPA DMEA 20min.amx	**Type:**	Sample
**Processing method:**	GC_LC Area Percent_DefaultMethod.pmx	**Calib level:**	
		**Sample amount:**	0.00
**Manually modified:**	Manual Integration	**ISTD amounts:**	None

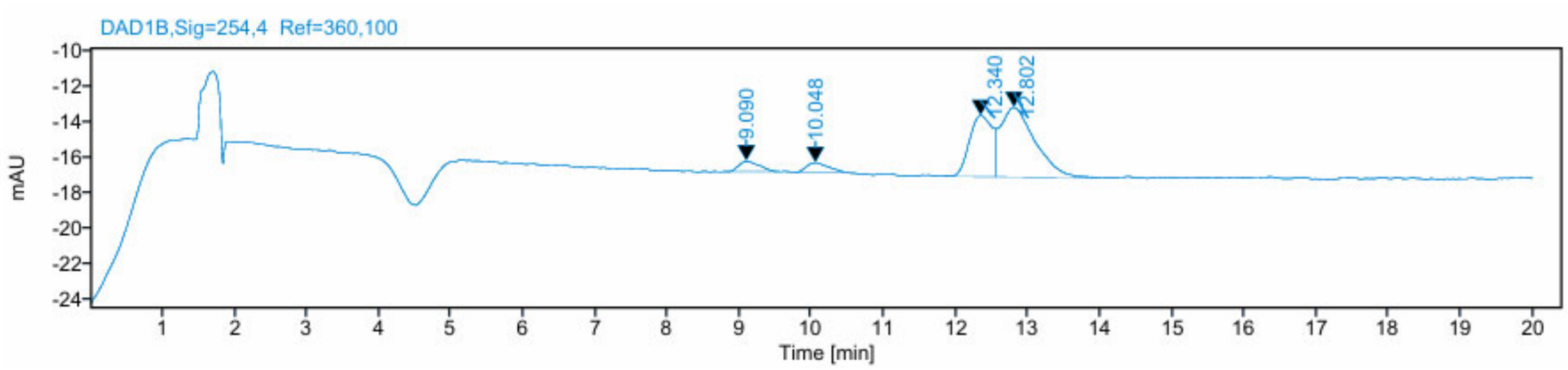

Signal: DAD1B,Sig=254,4 Ref=360,100

RT [min]	Type	Width [min]	Area	Height	Area%	Name
9.090	MM m	0.6689	11.2298	0.5906	4.8947	
10.048	MM m	0.7180	11.2534	0.5470	4.9050	
12.340	BV	0.7050	76.6688	3.4516	33.4173	
12.802	VB	1.3750	130.2765	3.9287	56.7830	
		**Sum**	**229.4285**			

SFC analysis of BMS Compound A (isomer 2). Conditions: Chiralpak IC column, flow rate = 2.0 mL/min; 35 °C; CO_2/*i*-PrOH with 0.1% dimethylethylamine (DMEA): 80/20. Integration of chromatogram shows almost exclusively a single enantiomer (ratio = 99.1:0.9) of separated stereoisomers (rt = 10.11 for major peak).

**Sample name:**	20-022-2		
**Data file:**	20-022-2SFC IC Iso 20% IPA DMEA 20min.amx20260512 162038.dx	**Operator:**	SYSTEM (SYSTEM)
**Instrument:**	SFC - HPLC	**Injection date:**	2026-05-12 16:56:39-04:00
**Inj. volume:**	2.000 µL	**Location:**	2
**Acq. method:**	SFC IC Iso 20% IPA DMEA 20min.amx	**Type:**	Sample
**Processing method:**	GC_LC Area Percent_DefaultMethod.pmx	**Calib level:**	
		**Sample amount:**	0.00
**Manually modified:**	Manual Integration	**ISTD amounts:**	None

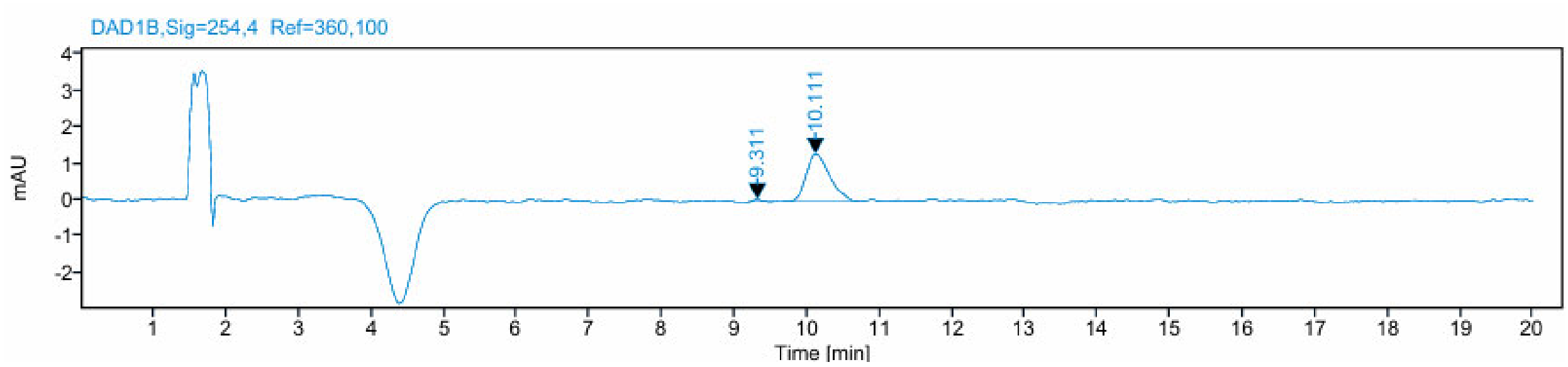

Signal: DAD1B,Sig=254,4 Ref=360,100

RT [min]	Type	Width [min]	Area	Height	Area%	Name
9.311	MM m	0.3471	0.2714	0.0557	0.9143	
10.111	MM m	0.9358	29.4088	1.3011	99.0857	
		**Sum**	**29.6801**			

1-(6-((1*S*,3*S*,6*R*)-Benzo[*d*][1,3]dioxol-5-yl)bicyclo[4.1.0]heptan-3-yl)-4-(2-bromo-5-chlorobenzyl)piperazine **BMS Compound A** (isomer 4). ^{1}H (400 MHz, $CDCl_3$), ^{13}C (126 MHz, $CDCl_3$).

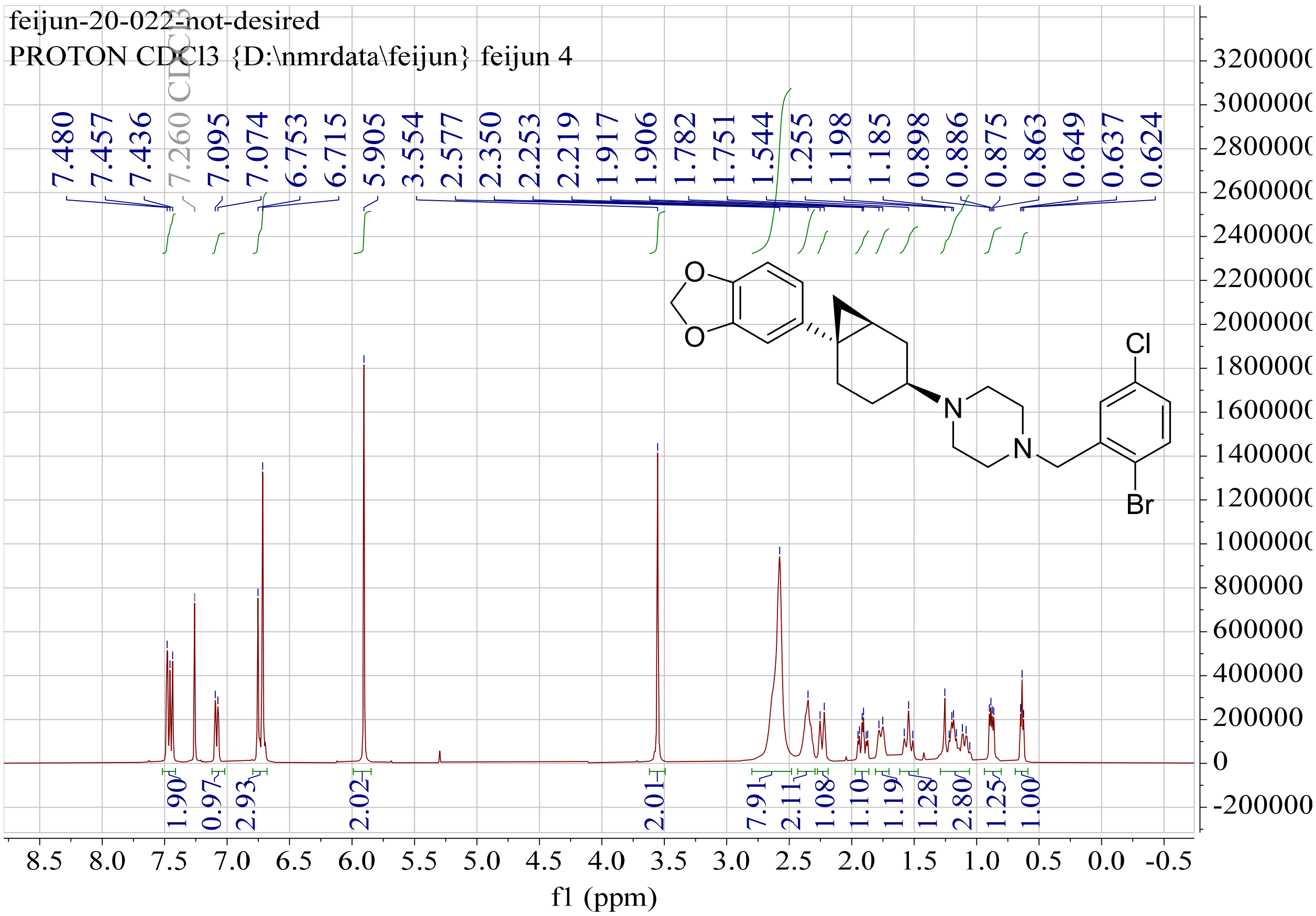
feijun-20-022-not-desired
PROTON CDCl3 {D:\nmrdata\feijun} feijun 4
7.480 7.457 7.436 7.260 CDCl3 7.095 7.074 6.753 6.715 5.905 3.554 2.577 2.350 2.253 2.219 1.917 1.906 1.782 1.751 1.544 1.255 1.198 1.185 0.898 0.886 0.875 0.863 0.649 0.637 0.624
1.90 0.97 2.93 2.02 2.01 7.91 2.11 1.08 1.10 1.19 1.28 2.80 1.25 1.00
O
O
Cl
N
N
Br
f1 (ppm)

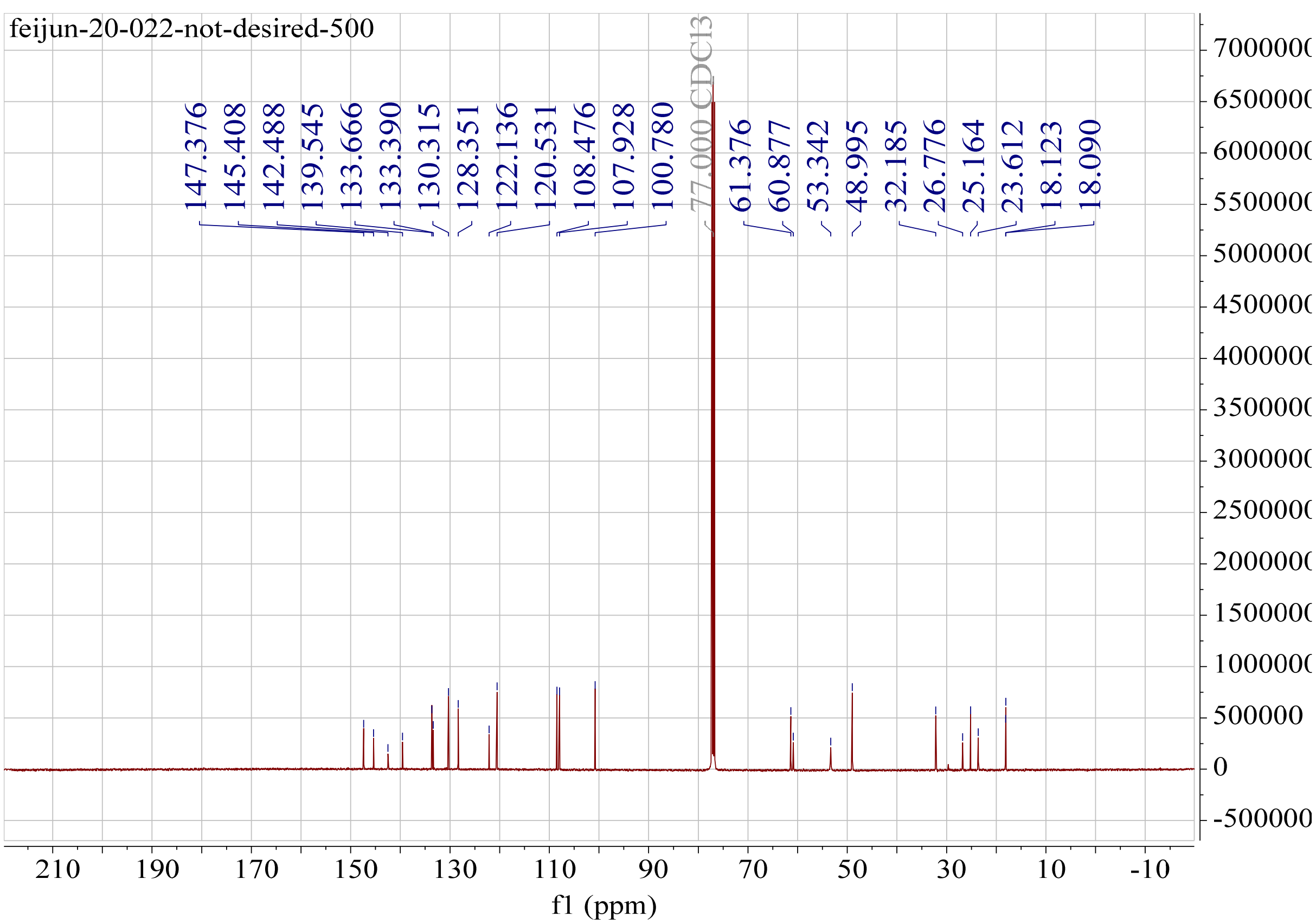
feijun-20-022-not-desired-500
147.376 145.408 142.488 139.545 133.666 133.390 130.315 128.351 122.136 120.531 108.476 107.928 100.780 77.000 CDCl3 61.376 60.877 53.342 48.995 32.185 26.776 25.164 23.612 18.123 18.090
f1 (ppm)

SFC separation of BMS Compound A (isomers 1, 2, 3 and 4): Conditions: Chiralpak IG column, flow rate = 2.5 mL/min, 35 °C, CO_2/*i*-PrOH with 0.1% dimethylethylamine (DMEA): 80/20. Integration of chromatogram shows ND:ND:49.9:50.1 ratio of separated stereoisomers (rt = 9.37, 9.37,13.61 and 16.47 minutes). These conditions provide clean separation of isomers 3 and 4 from each other and from their diastereomers (isomers 1 and 2), however no separation of isomers 1 and 2 from each other (see above for complementary conditions to cleanly separate isomers 1 and 2 from each other).

**Sample name:**	20-004		
**Data file:**	20-004SFC IG Iso 20% IPA-DMEA 20min.amx20260512 131743.dx	**Operator:**	SYSTEM (SYSTEM)
**Instrument:**	SFC - HPLC	**Injection date:**	2026-05-12 14:54:33-04:00
**Inj. volume:**	2.000 µL	**Location:**	3
**Acq. method:**	SFC IG Iso 20% IPA-DMEA 20min.amx	**Type:**	Sample
**Processing method:**	GC_LC Area Percent_DefaultMethod.pmx	**Calib level:**	
		**Sample amount:**	0.00
**Manually modified:**	Manual Integration	**ISTD amounts:**	None

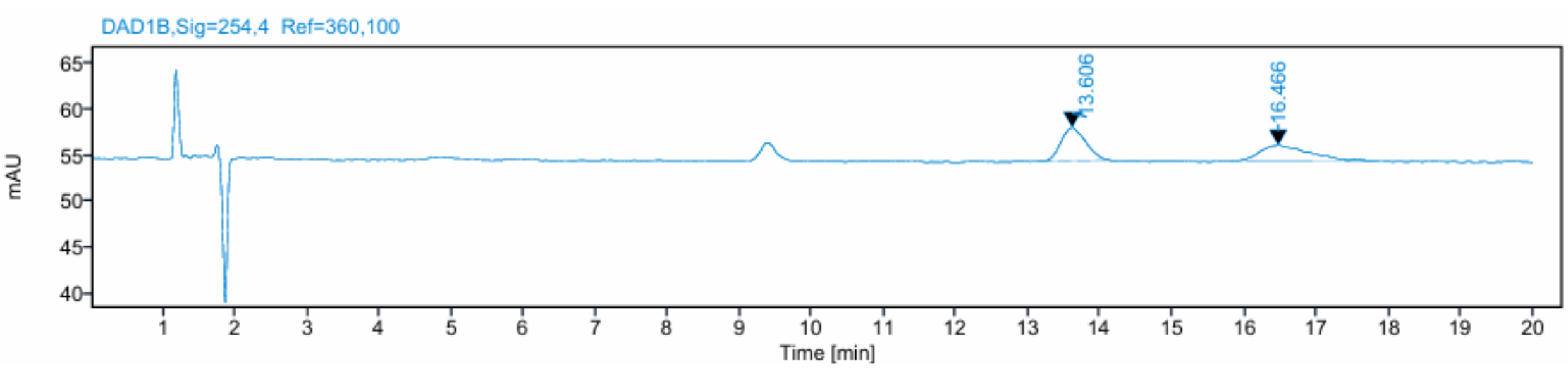

Signal: DAD1B,Sig=254,4 Ref=360,100

RT [min]	Type	Width [min]	Area	Height	Area%	Name
13.606	BM m	1.0301	86.2081	3.5775	49.9023	
16.466	BM m	2.0985	86.5458	1.7167	50.0977	
		**Sum**	**172.7540**			

SFC analysis of BMS Compound A (isomer 4). Conditions: Chiralpak IG column, flow rate = 2.5 mL/min; 35 °C; CO_2/*i*-PrOH with 0.1% dimethylethylamine (DMEA): 80/20. Integration of chromatogram shows almost exclusively a single enantiomer (ratio = 1.1:98.9) of separated stereoisomers (rt = 16.46 for major peak).

**Sample name:**	20-022-073re		
**Data file:**	20-022-073reSFC IG Iso 20% IPA-DMEA 20min.amx20260512 151525.dx	**Operator:**	SYSTEM (SYSTEM)
**Instrument:**	SFC - HPLC	**Injection date:**	2026-05-12 15:21:11-04:00
**Inj. volume:**	4.000 µL	**Location:**	5
**Acq. method:**	SFC IG Iso 20% IPA-DMEA 20min.amx	**Type:**	Sample
**Processing method:**	GC_LC Area Percent_DefaultMethod.pmx	**Calib level:**	
		**Sample amount:**	0.00
**Manually modified:**	Manual Integration	**ISTD amounts:**	None

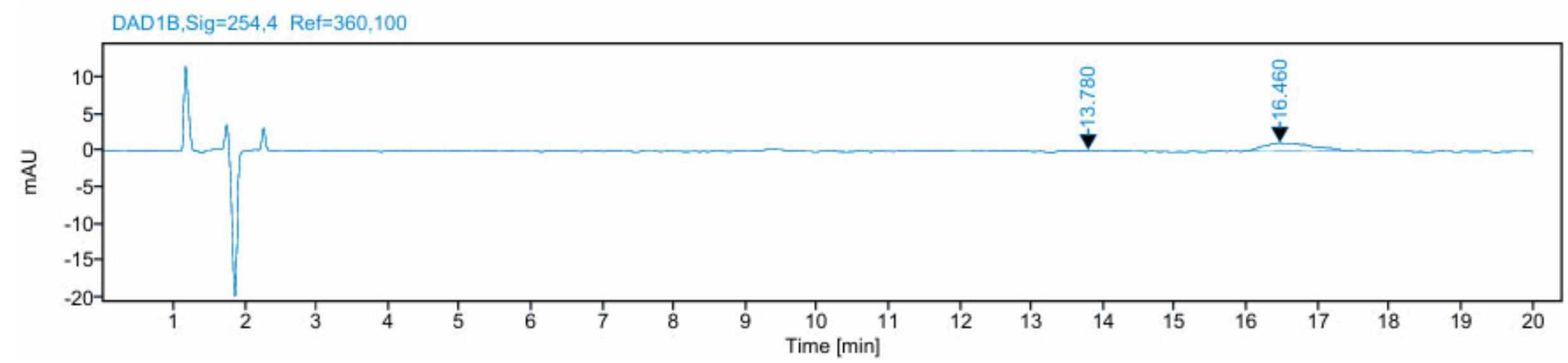

Signal: DAD1B,Sig=254,4 Ref=360,100

RT [min]	Type	Width [min]	Area	Height	Area%	Name
13.780	MM m	0.9102	0.5298	0.1467	1.0544	
16.460	MM m	1.4072	49.7189	1.1125	98.9456	
		**Sum**	**50.2487**			

Chiral HPLC Separation Report (analytical HPLC analysis of isolated isomers)

**Preparative Chromatography Conditions:**		
**Column:**	CHIRALPAK ADH	30x250mm
**Eluent:**	98/2/0.1 Hex/EtOH/DEA	
**Temperature °C:**	RT	
**UV Detection, nm:**	280	
**Flow Rate (ml/min):**	40	

RESULTS OF SEPARATION:

Diastereomer #	Mass (mg)	Purity	Yield*
1	44.5	97.8%	46.3%
2	40.9	91.2%	43.0%
3	67.4	98.6%	49.1%
4	68.6	93.9%	50.0%

* - Yield based on a ratio seen in starting material

Diastereomers #1, #2, #3, and #4 are the first, second, third, and fourth eluting peaks from the CHIRALPAK ADH column respectively.

```
Data File C:\CHEM32\2\DATA\PCT23026\PCT23026 2024-01-04 10-20-14\104-23026-RACE.D
Sample Name: Racemate

    =====================================================================
    Acq. Operator   : DPaicu                        Seq. Line :   1
    Acq. Instrument : HPLC 1100 Unit 2               Location : Vial 1
    Injection Date  : 1/4/2024 10:26:19 AM                Inj :   1
                                                   Inj Volume : 5.0 µl
    Different Inj Volume from Sequence !     Actual Inj Volume : 1.0 µl
    Acq. Method     : C:\CHEM32\2\DATA\PCT23026\PCT23026 2024-01-04 10-20-14\PCT23026.M
    Last changed    : 1/4/2024 10:16:05 AM by WSoder
    Analysis Method : C:\CHEM32\2\DATA\PCT23026\PCT23026 2024-01-04 10-20-14\104-23026-RACE.D\DA.M (
                      PCT23026.M, From Data File)
    Last changed    : 1/9/2024 1:09:00 PM by DPaicu
                      (modified after loading)
    Method Info     : PCT23026
                      ADH, 4.6x250mm
                      Hex-EtOH-DEA 98-2-0.1


    Sample Info     : PCT23026
                      UNC/NIH, 500mg
                      BMS-JC-015
                      Racemate


    Additional Info : Peak(s) manually integrated
```

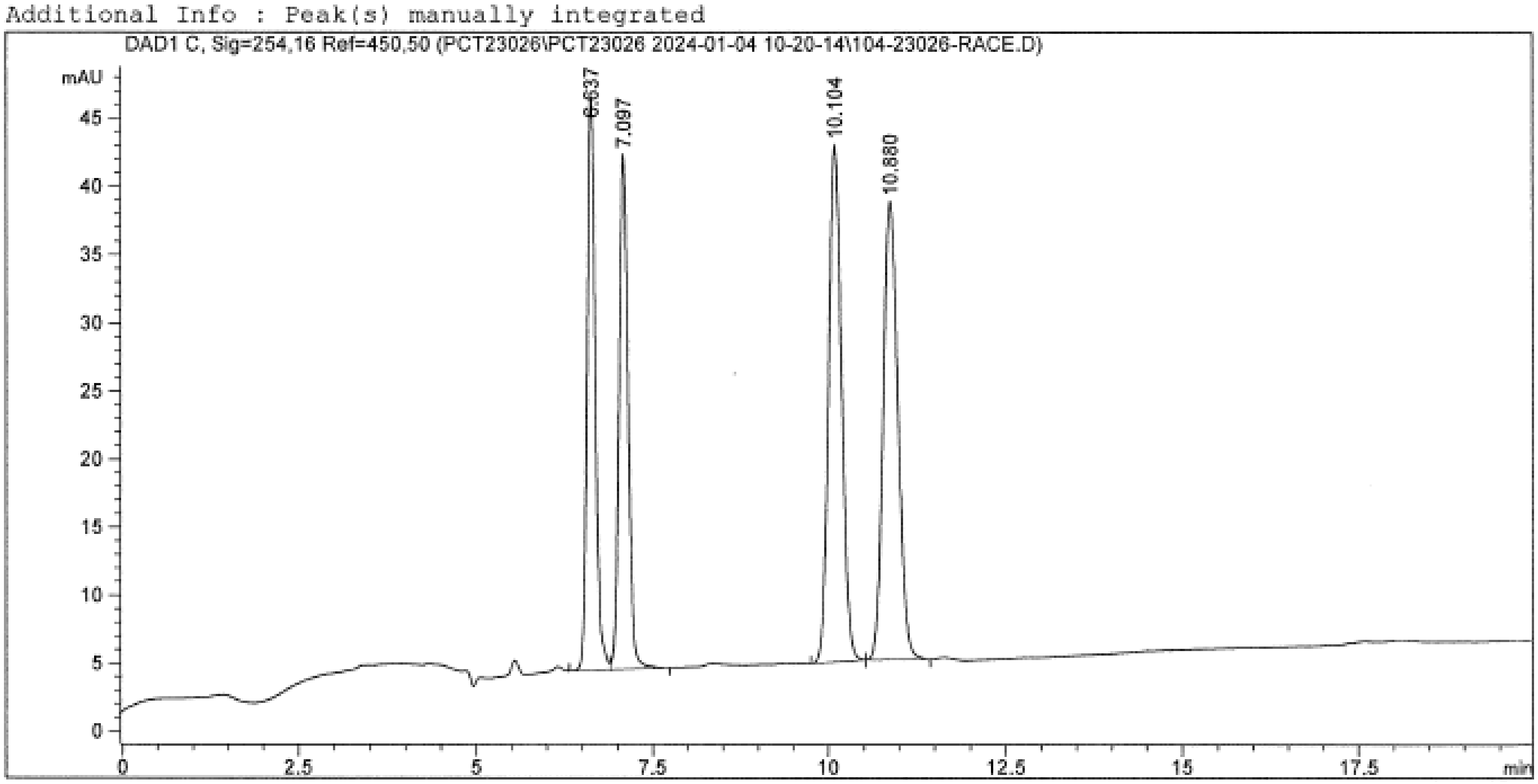


```
    =====================================================================
                             Area Percent Report
    =====================================================================

    Sorted By             :      Signal
    Calib. Data Modified  :      1/9/2024 1:08:46 PM
    Multiplier:                   :      1.0000
    Dilution:                     :      1.0000
    Do not use Multiplier & Dilution Factor with ISTDs
```

Data File C:\CHEM32\2\DATA\PCT23026\PCT23026 2024-01-04 10-20-14\104-23026-RACE.D
Sample Name: Racemate

Signal 1: DAD1 C, Sig=254,16 Ref=450,50

Peak #	RetTime [min]	Type	Width [min]	Area [mAU*s]	Area %	Name
1	6.637	BV	0.1249	341.92853	20.6315	Diastereomer #1
2	7.097	VB	0.1385	338.55005	20.4276	Diastereomer #2
3	10.104	BB	0.2023	488.64426	29.4841	Diastereomer #3
4	10.880	BB	0.2275	488.19226	29.4568	Diastereomer #4

Totals : 1657.31509

*** End of Report ***

```
Data File C:\CHEM32\2\DATA\PCT23026\PCT23026 2024-01-09 09-37-27\104-23026-F1.D
Sample Name: F1

=====================================================================
Acq. Operator   : DPaicu                         Seq. Line :   5
Acq. Instrument : HPLC 1100 Unit 2                Location : Vial 1
Injection Date  : 1/9/2024 10:56:39 AM                 Inj :   1
                                                Inj Volume : 5.0 µl
Different Inj Volume from Sequence !     Actual Inj Volume : 10.0 µl
Acq. Method     : C:\CHEM32\2\DATA\PCT23026\PCT23026 2024-01-09 09-37-27\PCT23026.M
Last changed    : 1/5/2024 12:48:21 PM by DPaicu
Analysis Method : C:\CHEM32\2\DATA\PCT23026\PCT23026 2024-01-04 12-57-56\PCT23026.M
Last changed    : 1/9/2024 1:32:13 PM by DPaicu
                  (modified after loading)
Method Info     : PCT23026
                  ADH, 4.6x250mm
                  Hex-EtOH-DEA 98-2-0.1


Sample Info     : PCT23026
                  UNC/NIH, 500mg
                  BMS-JC-015


Additional Info : Peak(s) manually integrated
```

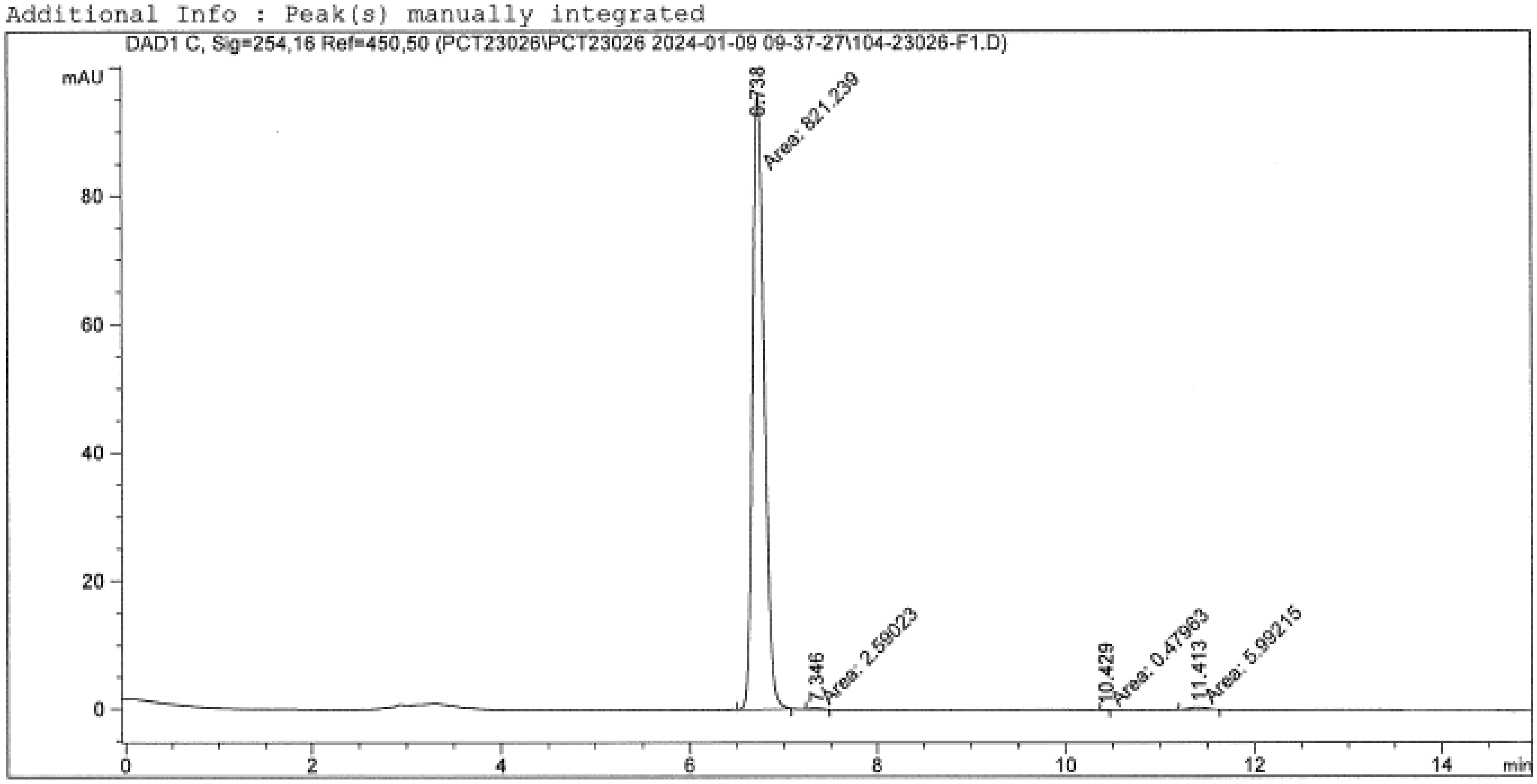


```
=====================================================================
                         Area Percent Report
=====================================================================

Sorted By              :      Signal
Calib. Data Modified   :      1/9/2024 1:32:13 PM
Multiplier:                    :      1.0000
Dilution:                      :      1.0000
Do not use Multiplier & Dilution Factor with ISTDs
```

Data File C:\CHEM32\2\DATA\PCT23026\PCT23026 2024-01-09 09-37-27\104-23026-F1.D
Sample Name: F1

Signal 1: DAD1 C, Sig=254,16 Ref=450,50

Peak #	RetTime [min]	Type	Width [min]	Area [mAU*s]	Area %	Name
1	6.738	MM	0.1430	821.23859	98.9086	Diastereomer #1
2	7.346	MM	0.1647	2.59023	0.3120	Diastereomer #2
3	10.429	MM	0.0925	4.79630e-1	0.0578	Diastereomer #3
4	11.413	MM	0.2503	5.99215	0.7217	Diastereomer #4

Totals : 830.30060

*** End of Report ***

```
Data File C:\CHEM32\2\DATA\PCT23026\PCT23026 2024-01-09 09-37-27\104-23026-F2.D
Sample Name: F2

=====================================================================
Acq. Operator   : DPaicu                          Seq. Line :   6
Acq. Instrument : HPLC 1100 Unit 2                 Location : Vial 2
Injection Date  : 1/9/2024 11:13:02 AM                  Inj :   1
                                                Inj Volume : 5.0 µl
Different Inj Volume from Sequence !     Actual Inj Volume : 10.0 µl
Acq. Method     : C:\CHEM32\2\DATA\PCT23026\PCT23026 2024-01-09 09-37-27\PCT23026.M
Last changed    : 1/5/2024 12:48:21 PM by DPaicu
Analysis Method : C:\CHEM32\2\DATA\PCT23026\PCT23026 2024-01-04 12-57-56\PCT23026.M
Last changed    : 1/9/2024 1:33:12 PM by DPaicu
                  (modified after loading)
Method Info     : PCT23026
                  ADH, 4.6x250mm
                  Hex-EtOH-DEA 98-2-0.1


Sample Info     : PCT23026
                  UNC/NIH, 500mg
                  BMS-JC-015


Additional Info : Peak(s) manually integrated
```

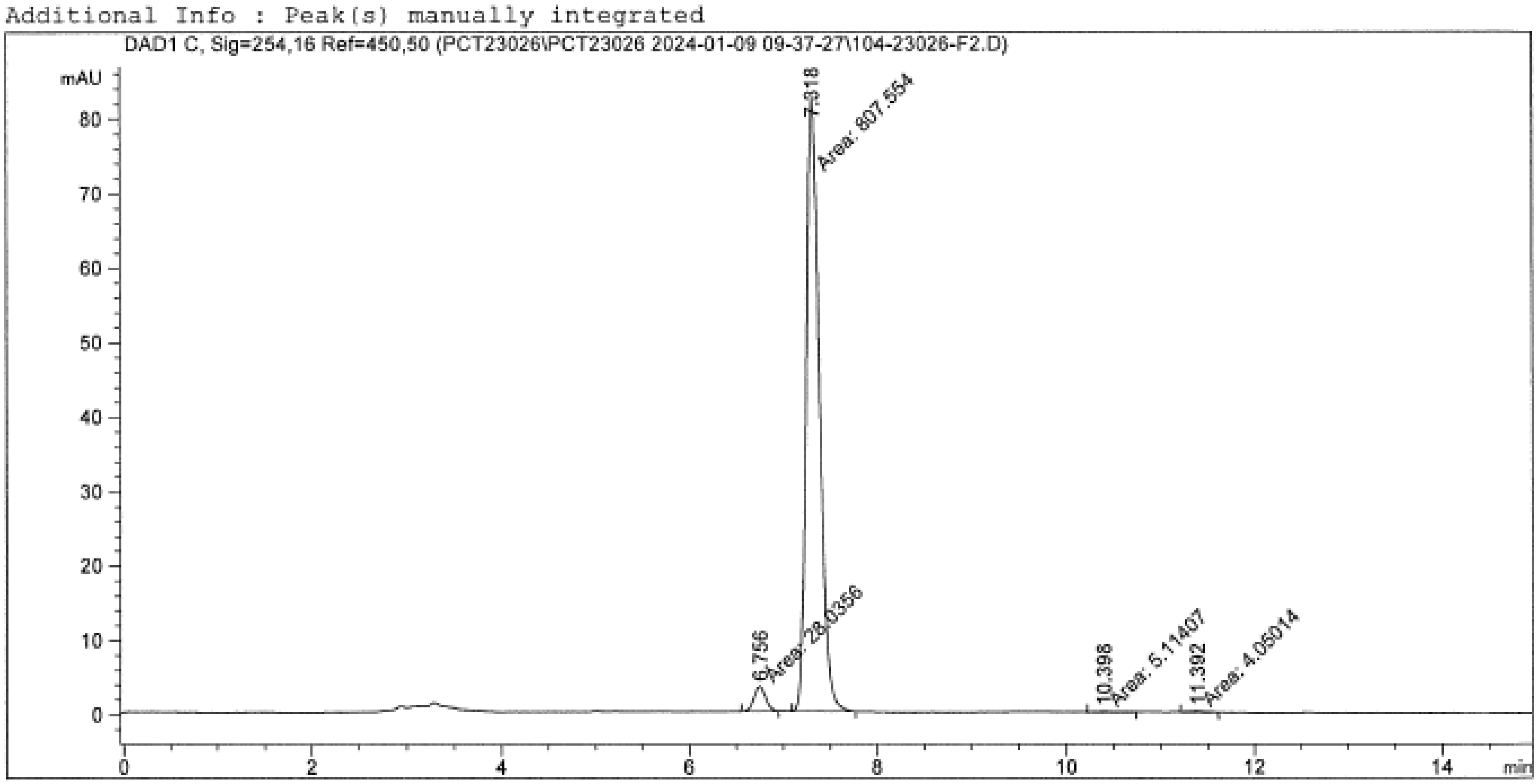


```
=====================================================================
                         Area Percent Report
=====================================================================

Sorted By             :      Signal
Calib. Data Modified  :      1/9/2024 1:33:12 PM
Multiplier:                   :      1.0000
Dilution:                     :      1.0000
Do not use Multiplier & Dilution Factor with ISTDs
```

Data File C:\CHEM32\2\DATA\PCT23026\PCT23026 2024-01-09 09-37-27\104-23026-F2.D
Sample Name: F2

Signal 1: DAD1 C, Sig=254,16 Ref=450,50

Peak #	RetTime [min]	Type	Width [min]	Area [mAU*s]	Area %	Name
1	6.756	MM	0.1418	28.03562	3.3188	Diastereomer #1
2	7.318	MM	0.1633	807.55383	95.5964	Diastereomer #2
3	10.398	MM	0.3938	5.11407	0.6054	Diastereomer #3
4	11.392	MM	0.2760	4.05014	0.4794	Diastereomer #4

Totals : 844.75366

*** End of Report ***

Data File C:\CHEM32\2\DATA\PCT23026\PCT23026 2024-01-09 12-05-23\104-23026-F3 FINAL.D
Sample Name: F3

===

Acq. Operator : DPaicu Seq. Line : 1
Acq. Instrument : HPLC 1100 Unit 2 Location : Vial 3
Injection Date : 1/9/2024 12:16:41 PM Inj : 1
Inj Volume : 5.0 µl
Different Inj Volume from Sequence ! Actual Inj Volume : 20.0 µl
Acq. Method : C:\CHEM32\2\DATA\PCT23026\PCT23026 2024-01-09 12-05-23\PCT23026.M
Last changed : 1/5/2024 12:48:21 PM by DPaicu
Analysis Method : C:\CHEM32\2\DATA\PCT23026\PCT23026 2024-01-04 12-57-56\PCT23026.M
Last changed : 1/9/2024 1:34:51 PM by DPaicu
(modified after loading)
Method Info : PCT23026
ADH, 4.6x250mm
Hex-EtOH-DEA 98-2-0.1

Sample Info : PCT23026
UNC/NIH, 500mg
BMS-JC-015

Additional Info : Peak(s) manually integrated

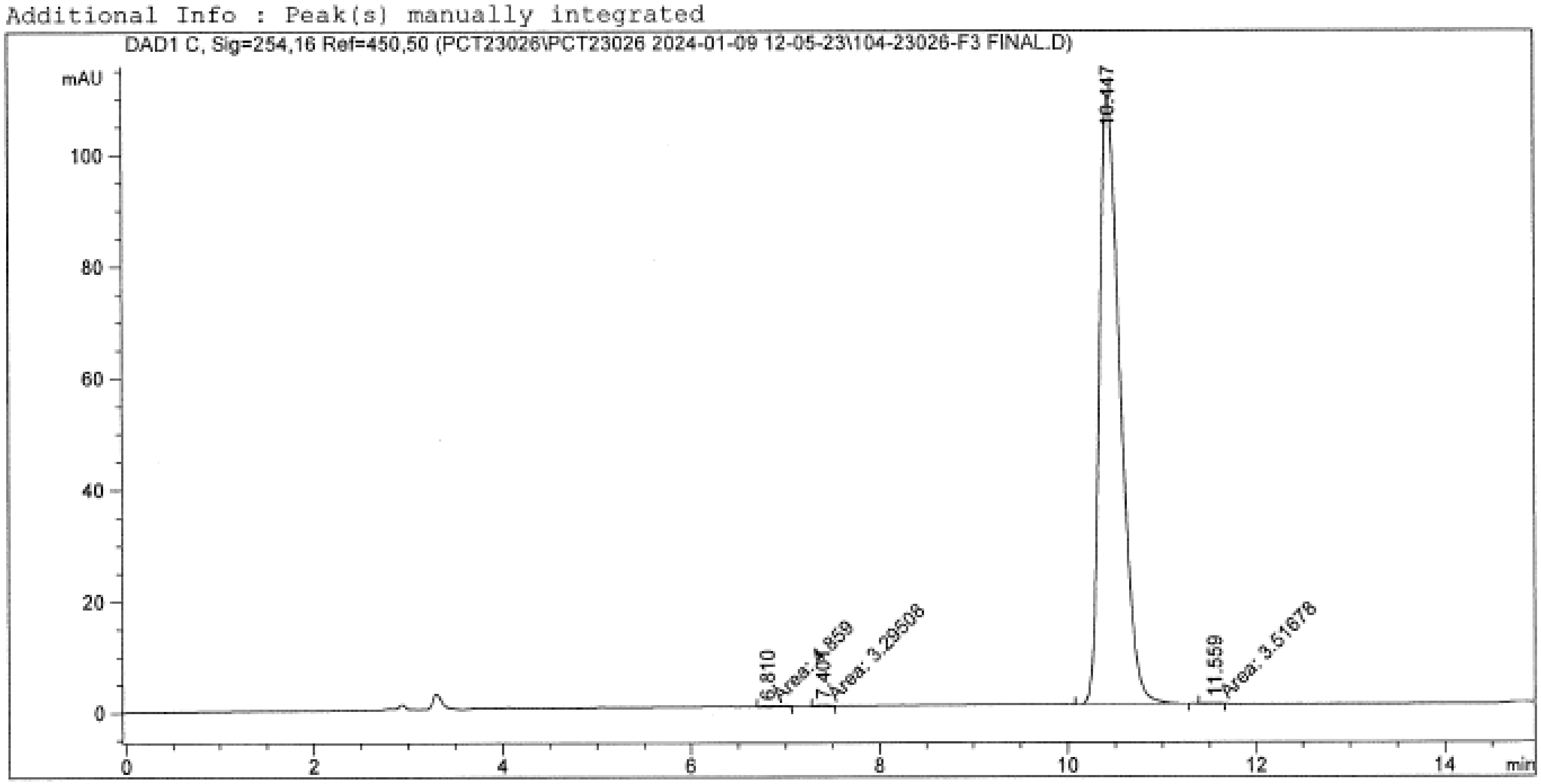

===
Area Percent Report
===

Sorted By : Signal
Calib. Data Modified : 1/9/2024 1:34:51 PM
Multiplier: : 1.0000
Dilution: : 1.0000
Do not use Multiplier & Dilution Factor with ISTDs

Data File C:\CHEM32\2\DATA\PCT23026\PCT23026 2024-01-09 12-05-23\104-23026-F3 FINAL.D
Sample Name: F3

Signal 1: DAD1 C, Sig=254,16 Ref=450,50

Peak #	RetTime [min]	Type	Width [min]	Area [mAU*s]	Area %	Name
1	6.810	MM	0.2846	4.85900	0.2889	Diastereomer #1
2	7.401	MM	0.1909	3.29508	0.1959	Diastereomer #2
3	10.447	BB	0.2365	1670.08508	99.3060	Diastereomer #3
4	11.559	MM	0.2492	3.51678	0.2091	Diastereomer #4

Totals : 1681.75594

*** End of Report ***

Data File C:\CHEM32\2\DATA\PCT23026\PCT23026 2024-01-09 09-37-27\104-23026-F4 FINAL.D
Sample Name: F4

```
=====================================================================
Acq. Operator   : DPaicu                          Seq. Line :   4
Acq. Instrument : HPLC 1100 Unit 2                 Location : Vial 4
Injection Date  : 1/9/2024 10:40:16 AM                  Inj :   1
                                                Inj Volume : 5.0 µl
Different Inj Volume from Sequence !     Actual Inj Volume : 10.0 µl
Acq. Method     : C:\CHEM32\2\DATA\PCT23026\PCT23026 2024-01-09 09-37-27\PCT23026.M
Last changed    : 1/5/2024 12:48:21 PM by DPaicu
Analysis Method : C:\CHEM32\2\DATA\PCT23026\PCT23026 2024-01-09 09-37-27\104-23026-F4 FINAL.D\
                  DA.M (PCT23026.M, From Data File)
Last changed    : 1/9/2024 1:28:00 PM by DPaicu
Method Info     : PCT23026
                  ADH, 5um, 4.6x250mm
                  Hex-EtOH-DEA 98-2-0.1
```

Additional Info : Peak(s) manually integrated

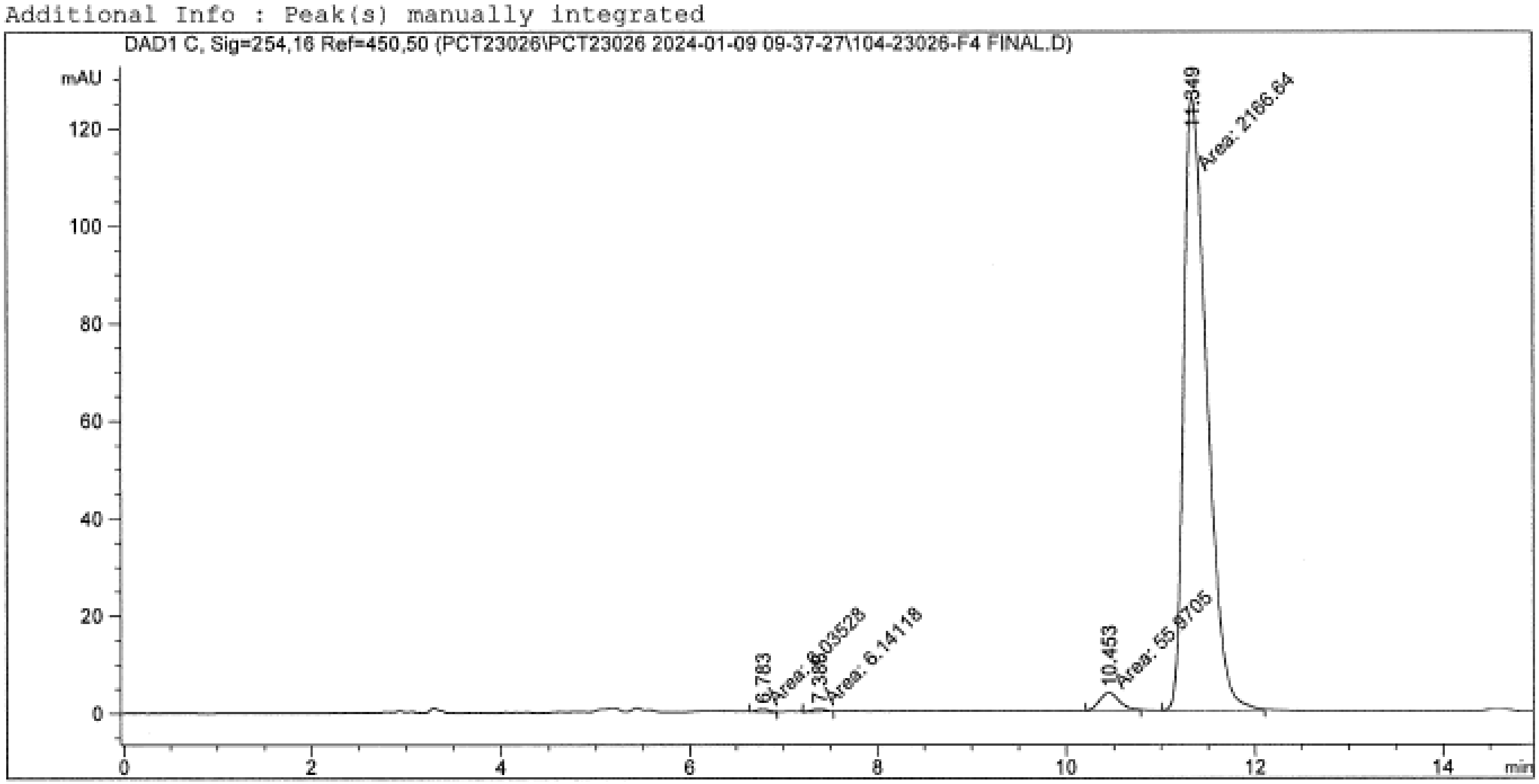


```
=====================================================================
                        Area Percent Report
=====================================================================

Sorted By             :      Signal
Calib. Data Modified  :      1/9/2024 1:26:40 PM
Multiplier:                   :      1.0000
Dilution:                     :      1.0000
Do not use Multiplier & Dilution Factor with ISTDs


Signal 1: DAD1 C, Sig=254,16 Ref=450,50
```

Peak #	RetTime [min]	Type	Width [min]	Area [mAU*s]	Area %	Name
1	6.783	MM	0.1649	6.03528	0.2701	Diastereomer #1
2	7.386	MM	0.1800	6.14118	0.2748	Diastereomer #2

```
Data File C:\CHEM32\2\DATA\PCT23026\PCT23026 2024-01-09 09-37-27\104-23026-F4 FINAL.D
Sample Name: F4

     Peak RetTime  Type   Width     Area      Area    Name
       #   [min]          [min]   [mAU*s]      %
     ----|-------|-------|-------|----------|--------|---------------------
        3  10.453 MM      0.2513   55.97045   2.5045 Diastereomer #3
        4  11.349 MM      0.2868 2166.64355  96.9506 Diastereomer #4

     Totals :                    2234.79047

     ==================================================================
                                *** End of Report ***
```

Figure S1. Concentration–response curves of BMS Compound A isomers for potentiating dopamine-stimulated β-arrestin recruitment to the D1R. The DiscoveRx Pathhunter β-arrestin recruitment assays were conducted as described in the Experimental Section. Dopamine concentration-response curves were performed in the absence or presence of the indicated concentrations of each isomer. The lowest concentration in each curve was performed in the absence of dopamine and used to define the baseline response (0%). The dopamine E_{max} in the absence of PAMs was used to define the maximal response (100%) in each experiment. The data are representative of three independent experiments. The tables below each graph provide the mean curve parameters across the three experiments. Using the DA EC_{50} fold-shift as the most relevant variable, each isomer exhibits a maximal effect between a concentration of 20–30 mM.

Supplemental Figure 1.

A

BMS Isomer 1

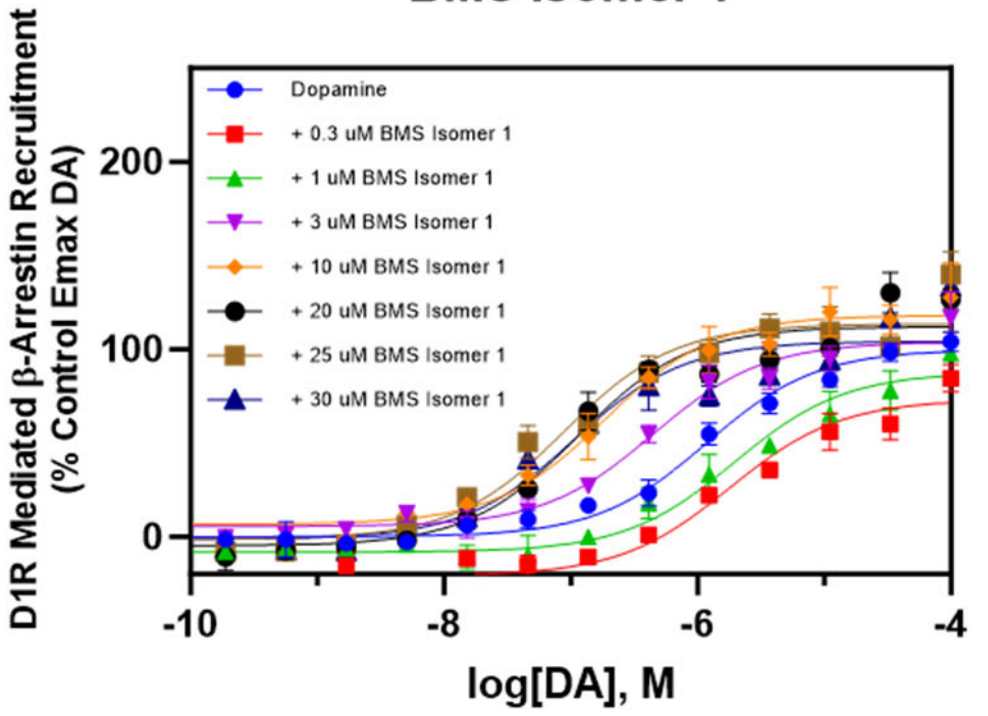

Condition	EC_{50} (nM)	Fold-Shift	Emax (% DA)
DA	1184 ± 43.5	1.0	100%
0.3 μM	1947 ± 341	0.64 ± 0.08	73.9 ± 3.44%
1 μM	2357 ± 901	0.79 ± 0.41	89.5 ± 4.16%
3 μM	476 ± 69.1	2.61 ± 0.41	104 ± 6.08%
10 μM	310 ± 69.1	7.43 ± 3.67	121 ± 7.77%
20 μM	119 ± 24.3	10.9 ± 2.30	112 ± 2.86%
25 μM	88.4 ± 13.2	14.1 ± 2.28	113 ± 5.78%
30 μM	172 ± 88.7	11.2 ± 4.38	107 ± 7.26%

B

BMS Isomer 2

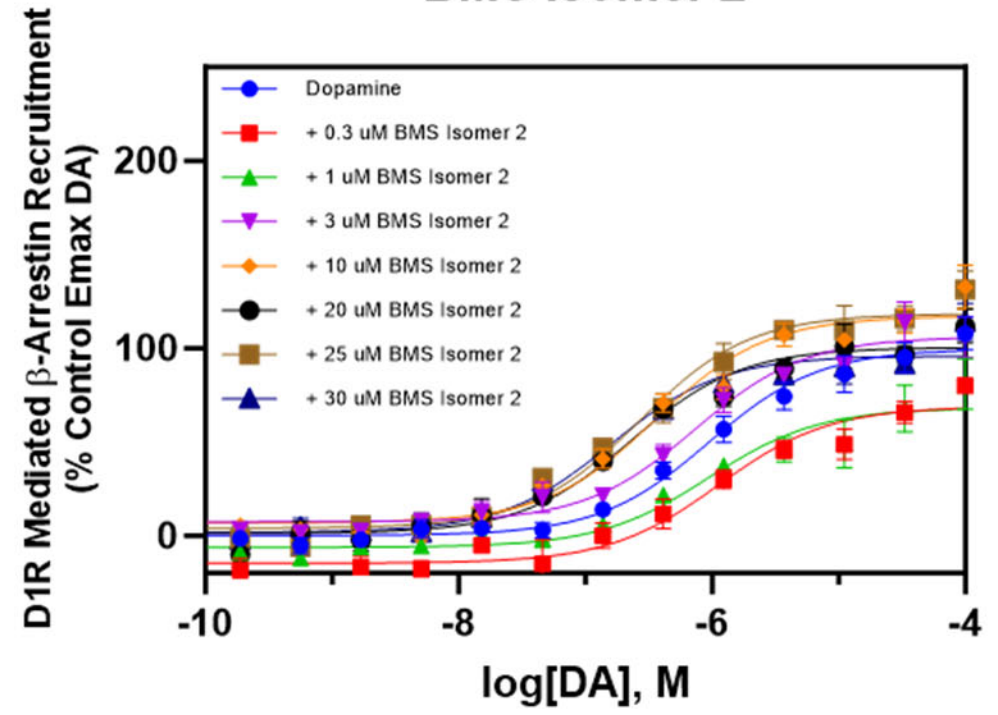

Condition	EC_{50} (nM)	Fold-Shift	Emax (% DA)
DA	1017 ± 256	1.0	100%
0.3 μM	1130 ± 5.69	0.90 ± 0.22	68.9 ± 4.84%
1 μM	1160 ± 502	1.76 ± 1.03	69.7 ± 15.0%
3 μM	771 ± 191	1.37 ± 0.21	106 ± 4.95%
10 μM	363 ± 74.9	3.18 ± 1.04	117 ± 4.28%
20 μM	228 ± 8.77	4.39 ± 0.95	100 ± 2.07%
25 μM	274 ± 57.5	4.20 ± 1.31	119 ± 7.47%
30 μM	161 ± 10.6	6.50 ± 1.86	96.1 ± 4.62%

C

BMS Isomer 3

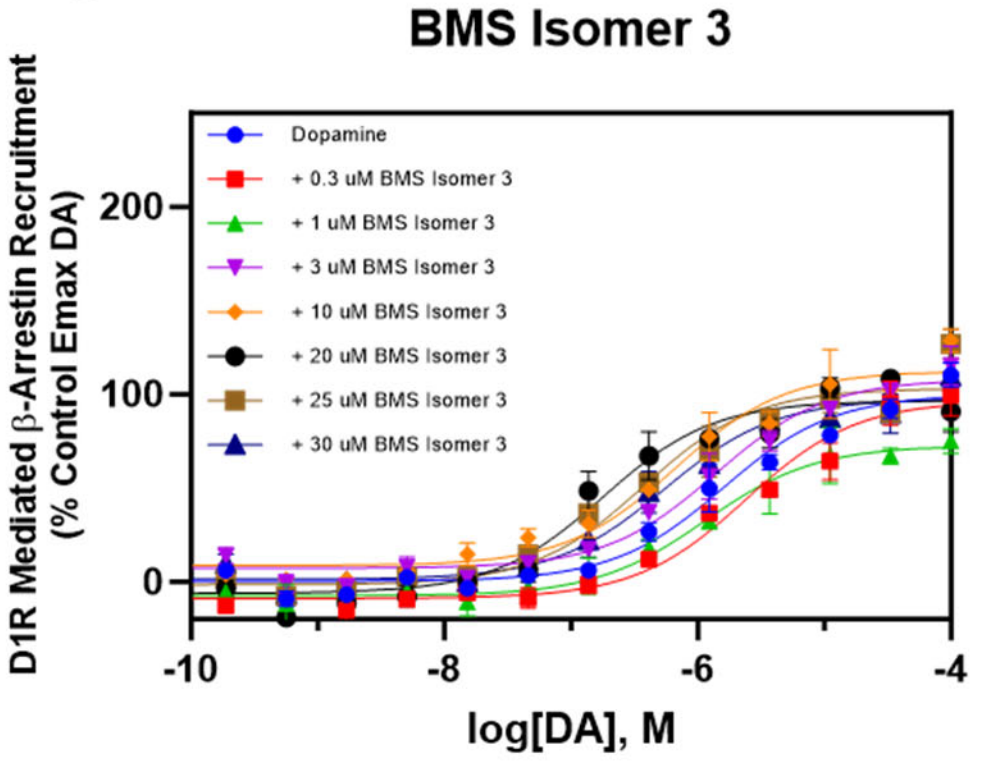

Condition	EC_{50} (nM)	Fold-Shift	Emax (% DA)
DA	1744 ± 638	1.0	100%
0.3 μM	2811 ± 718	0.72 ± 0.26	99.0 ± 7.97%
1 μM	1126 ± 119	1.47 ± 0.38	72.5 ± 9.44%
3 μM	1452 ± 205	1.14 ± 0.27	108 ± 4.13%
10 μM	681 ± 92.4	2.72 ± 1.04	113 ± 3.24%
20 μM	236 ± 93.1	14.5 ± 10.8	98.3 ± 6.44%
25 μM	421 ± 82.4	5.29 ± 3.00	104 ± 2.98%
30 μM	690 ± 372	5.25 ± 3.31	98.5 ± 3.12%

D

BMS Isomer 4

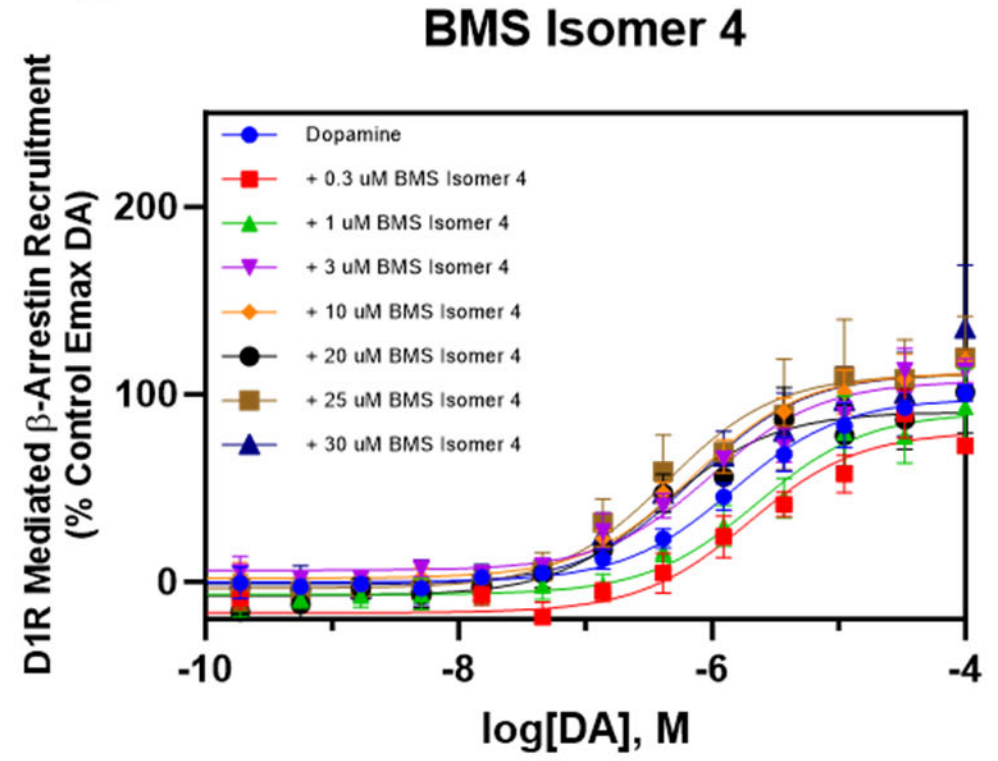

Condition	EC_{50} (nM)	Fold-Shift	Emax (% DA)
DA	2092 ± 862	1.0	100%
0.3 uM	2046 ± 477	1.05 ± 0.32	78.0 ± 5.73%
1 uM	1894 ± 631	1.17 ± 0.29	76.1 ± 8.00%
3 uM	980 ± 326	2.13 ± 0.36	97.9 ± 3.76%
10 uM	553 ± 232	6.90 ± 3.83	111 ± 8.75%
20 uM	266 ± 102	9.25 ± 2.99	85.1 ± 13.2%
25 uM	368 ± 164	7.64 ± 3.12	100 ± 13.0%
30 uM	520 ± 284	6.15 ± 2.82	96.0 ± 11.4%

Table S1. Crystal data and structure refinement for BMS Compound A (isomer 1).

Empirical formula	C25 H30 Br1 Cl3 N2 O2	
Formula weight	576.79	
Crystal color, shape, size	colorless plate, 0.150 x 0.050 x 0.010 mm^3	
Temperature	150 K	
Wavelength	1.54180 Å	
Crystal system, space group	Monoclinic, P 1 21 1	
Unit cell dimensions	a = 11.8545(3) Å	α= 90°.
	b = 6.9877(2) Å	β= 107.5080(10)°.
	c = 15.8499(4) Å	γ = 90°.
Volume	1252.12(6) $Å^3$	
Z	2	
Density (calculated)	1.530 Mg/m^3	
Absorption coefficient	5.389 mm^{-1}	
F(000)	592	
Data collection		
Diffractometer	Bruker D8 VENTURE, Bruker	
Theta range for data collection	2.923 to 70.130°.	
Index ranges	-14<=h<=14, -8<=k<=8, -19<=l<=19	
Reflections collected	19418	
Independent reflections	4730 [R(int) = 0.035]	
Observed Reflections	4618	
Completeness to theta = 70.130°	99.9 %	
Solution and Refinement		
Absorption correction	Semi-empirical from equivalents	
Max. and min. transmission	0.95 and 0.76	
Solution	Charge flipping	
Refinement method	Full-matrix least-squares on F^2	
Weighting scheme	$w = [\sigma^2 Fo^2 + AP^2 + BP]^{-1}$, with	
	$P = (Fo^2 + 2 Fc^2)/3$, A = 0.038 , B = 0.940	
Data / restraints / parameters	4706 / 11 / 307	
Goodness-of-fit on F^2	1.0130	
Final R indices [I>2sigma(I)]	R1 = 0.0276, wR2 = 0.0709	
R indices (all data)	R1 = 0.0281, wR2 = 0.0713	
Absolute structure parameter	-0.015(13)	
Largest diff. peak and hole	0.57 and -0.29 e.$Å^{-3}$	

Table S1. Crystal data and structure refinement for BMS Compound A (isomer 1).

Empirical formula	C25 H29 Br1 Cl2 N2 O2	
Formula weight	540.33	
Crystal color, shape, size	colorless plat, 0.040 x 0.040 x 0.010 mm^3	
Temperature	150 K	
Wavelength	1.54180 Å	
Crystal system, space group	Monoclinic, P 1 21 1	
Unit cell dimensions	a = 11.3024(6) Å	α= 90°.
	b = 5.9715(3) Å	β= 91.392(3)°.
	c = 35.6515(18) Å	γ = 90°.
Volume	2405.5(2) $Å^3$	
Z	4	
Density (calculated)	1.492 Mg/m^3	
Absorption coefficient	4.573 mm^{-1}	
F(000)	1112	
Data collection		
Diffractometer	Bruker D8 VENTURE, Bruker	
Theta range for data collection	3.720 to 70.141°.	
Index ranges	-13<=h<=13, -7<=k<=6, -43<=l<=43	
Reflections collected	81105	
Independent reflections	8998 [R(int) = 0.059]	
Observed Reflections	8192	
Completeness to theta = 70.141°	99.7 %	
Solution and Refinement		
Absorption correction	Semi-empirical from equivalents	
Max. and min. transmission	0.96 and 0.83	
Solution	Intrinsic phasing methods	
Refinement method	Full-matrix least-squares on F^2	
Weighting scheme	$w = [\sigma^2 Fo^2 + AP^2 + BP]^{-1}$, with	
	$P = (Fo^2 + 2 Fc^2)/3$, A =0.105 , B = 10.570	
Data / restraints / parameters	8959 / 106 / 586	
Goodness-of-fit on F^2	1.0040	
Final R indices [I>2sigma(I)]	R1 = 0.0737, wR2 = 0.2020	
R indices (all data)	R1 = 0.0776, wR2 = 0.2056	
Absolute structure parameter	0.326(12)	
Largest diff. peak and hole	0.89 and -0.49 e.$Å^{-3}$	
